# Single-Molecule Conductance of Neutral Closed-Shell and Open-Shell Diradical Indenofluorenes

**DOI:** 10.1021/jacs.4c13551

**Published:** 2024-10-18

**Authors:** Raquel Casares, Sandra Rodríguez-González, Álvaro Martínez-Pinel, Irene R. Márquez, M. Teresa González, Cristina Díaz, Fernando Martín, Juan M. Cuerva, Edmund Leary, Alba Millán

**Affiliations:** † Departamento de Química Orgánica, Unidad de Excelencia de Química Aplicada a Biomedicina y Medioambiente (UEQ), C. U. Fuentenueva, Universidad de Granada, Granada 18071, Spain; ‡ Departamento de Química Física Aplicada, Universidad Autónoma de Madrid, Madrid 28049, Spain; § Centro de Instrumentación Científica, Universidad de Granada, Granada 18071, Spain; ∥ Fundación IMDEA Nanociencia, Madrid 28049, Spain; ⊥ Departamento de Química Física, Facultad de Ciencias Químicas, Universidad Complutense de Madrid, Madrid 28040, Spain; # Departamento de Química, Módulo 13, Universidad Autónoma de Madrid, Madrid 28049, Spain

## Abstract

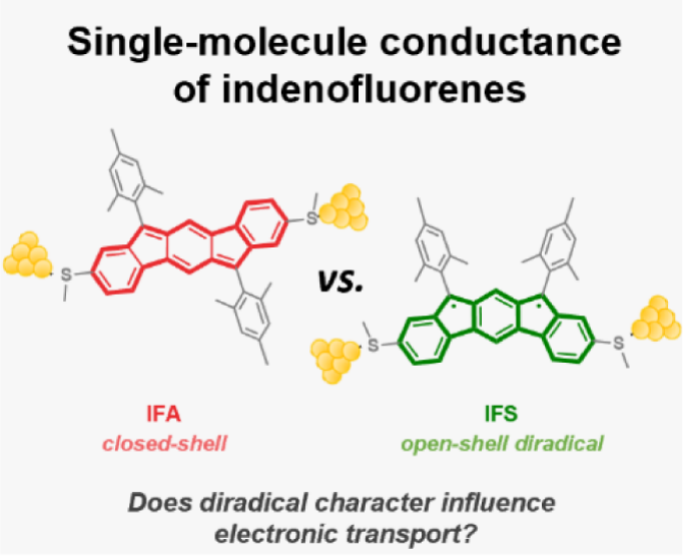

Organic diradicals are highly promising candidates as future components in molecular electronic and spintronic devices because of their low spin–orbit coupling. To advance toward final circuit realizations, a thorough knowledge of the behavior of diradicals within a single-molecule junction framework is imperative. In this work, we have measured for the first time the single-molecule conductance of a neutral open-shell diradical compound, a [2,1-*b*] isomer of indenofluorene (IF). Our results reveal that the conductance of the [2,1-*b*] isomer is about 1 order of magnitude higher than that of the corresponding closed-shell regioisomer [1,2-*b*] IF. This is significant, as it fundamentally demonstrates the possibility of forming stable single-molecule junctions using neutral diradical compounds which are also highly conducting. This opens up a new approach to the development of externally addressable spintronic devices operable at room temperature.

## Introduction

Organic radicals are highly appealing building blocks for creating novel molecular spintronic devices due to the typically low spin–orbit coupling and hyperfine interactions.^1^ To reach this ultimate goal, one has first to understand their behavior within the testbed environment of single-molecule junctions, which allow for a reliable characterization of the transport properties of individual molecules.^2^ Various compounds that display stable and localized monoradical character have been studied by means of either scanning tunneling microscope-break junction (STM-BJ) or mechanically controllable break-junction (MCBJ) techniques.^3−8^ Recently, several cationic compounds, with more than one radical center, have also been explored.^9^ A key question in these studies is whether the molecules retain their particular electronic structure inside the junction, which cannot be easily determined from room-temperature measurements. Assuming that the electronic structure indeed persists in the junction, does it actually have a significant contribution to the electronic transport?

Another important point for everyday applications in molecular spintronics is that devices should ideally operate at close to room temperature and, if possible, under ambient conditions.^10^ Monoradicals have limited scope in this regard since a switchable magnetic behavior, such as spin flips, is only possible at low temperatures.^11^ In contrast, diradicals can be designed with magnetically active triplet states, thermally accessible close to room temperature.^12^ Switching processes between singlet and triplet states can then be induced by external stimuli (e.g., via temperature or applied magnetic fields).^13^ This possibility allows for new spintronic devices, such as bistable molecular-based memories, which are closer to those required in actual devices.^14^ Due to their inherent instability, however, and challenging synthesis and measurement, single-molecule conductance studies of neutral open-shell organic diradicals at room temperature have not been reported so far.^15^

**Figure 1:**
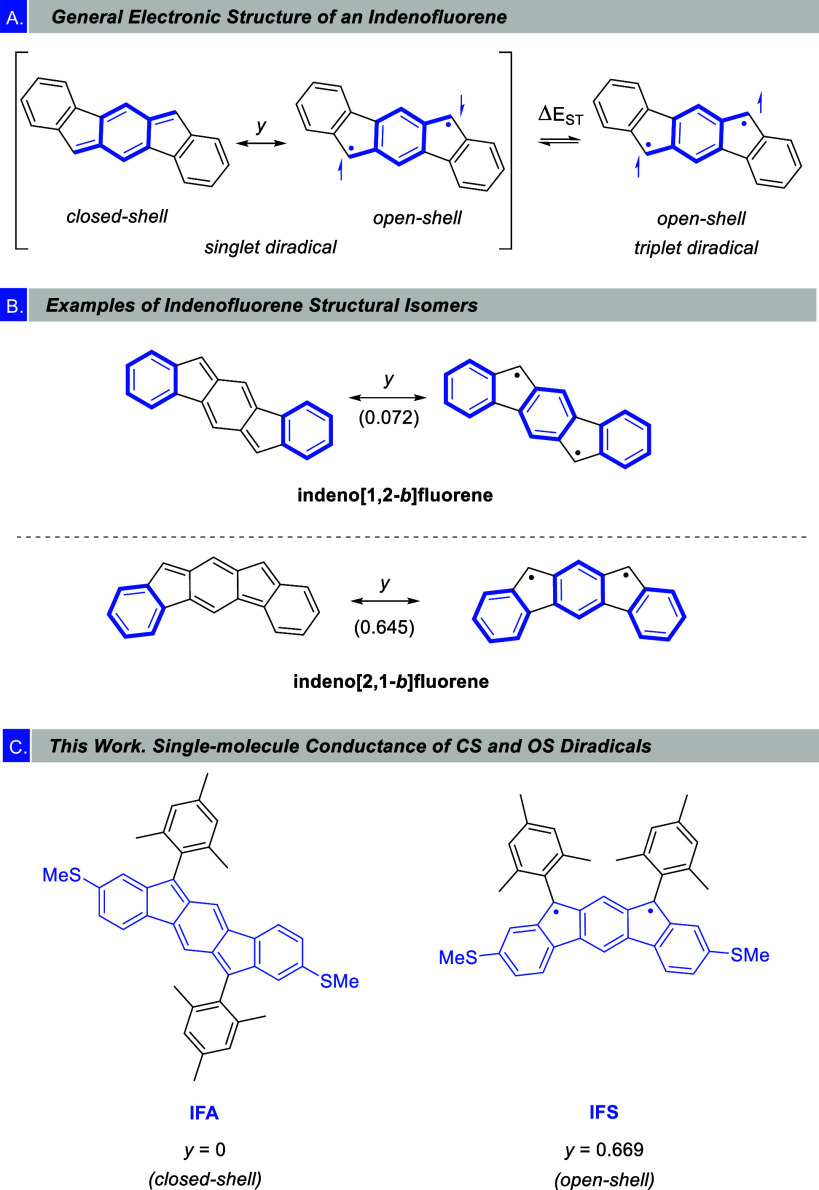
(A) *p*-Quinodimethane resonance form of indeno[1,2-*b*]fluorene. (B) Examples of two of the five possible structural isomers of the indenofluorene core and their diradical character indexes (*y*). Clar sextets are highlighted in blue. Diradical character index values were taken from reference^18^. (C) Our target molecules for single-molecule conductance studies. *y* values obtained in this work (see Results and Discussion).

In this context, indenofluorene (IF) scaffolds have been extensively studied over the past decade, not only to unravel their fundamental properties, but also to evaluate possible application in organic optoelectronics.^16^ IF is formally considered the Hückel antiaromatic analogue of pentacene, bearing alternating five- and six-membered rings and, thus, 20 π-electrons in its polycyclic conjugated core. Its structure has a pro-aromatic central quinodimethane unit (QDM), which can provide diradical character (Figure 1A), and its electronic structure in the ground state is described as a mixture of the Kekulé closed-shell (CS) and non-Kekulé open-shell (OS) configurations. The contribution of the OS configuration to the overall structure is given by the diradical character index, *y* (0 (CS) ≤ *y* ≤ 1 (OS)).^17^ Depending on the relative fusion pattern of the rings, different regioisomers are possible, covering a wide range of diradical character indexes.^18^ High *y* values can be rationalized by considering the gain in the number of Clar sextets from the CS configuration (1 sextet) to the OS forms (3 sextets) (Figure 1B, bottom) and correlates with the thermal accessibility of the triplet (T) state. The synthesis of the five possible IF structural isomers has previously been accomplished in solution by inclusion of either bulky units or spin-delocalizing groups on the reactive bridging carbons of the five-membered rings.^19−23^ Thus, their structural, optoelectronic and magnetic properties have been studied in detail through kinetic or thermodynamic stabilization of the molecule.^24^ In contrast, no studies on the single-molecule conductance across any type of IF core have been reported.^20e,25^ We have, therefore, investigated the behavior of formally antiaromatic IFs in a molecular junction environment operating under ambient conditions.

**Figure 2:**
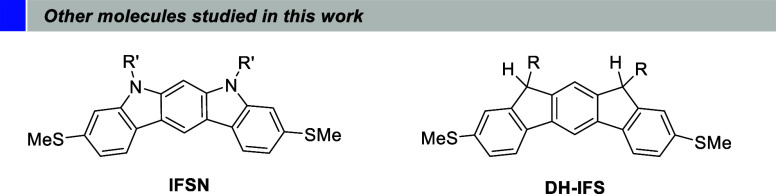
Structures of other molecules studied in this work. R = 2,4,6-trimethylphenyl; R’= *n*-hexyl.

Aiming to shed some light on the relationship between the diradical index and the conductance, we have synthesized two IF regioisomers: indeno[1,2-*b*]fluorene (**IFA**) and indeno[2,1-*b*]fluorene isomer (**IFS**) (Figure 1C, where A and S stand for the relative orientation of the apical carbon of the five-membered ring, *anti* and *syn* respectively), which display opposite diradical character, zero for the former while the latter has a significant nonzero value. Each molecule bears two thiomethyl groups (−SMe) as anchor groups located *meta* to the bridging carbon atoms of the five-membered rings. Also, 2,4,6-trimethylphenyl (mesityl, Mes) groups were introduced at the reactive positions of the five-membered rings to enhance molecular stability. Additionally, we prepared two structurally related analogues to **IFS**, but with no diradical character: the indolocarbazole **IFSN** and the dihydroindenofluorene **DH-IFS** (Figure 2). We then used the scanning tunneling break junction (STM-BJ) technique,^26^ operating under ambient conditions, to form single-molecule junctions and evaluate the molecular conductance. We were able to detect clear and well-defined single-molecule signals from all the studied compounds, and we observed different conductance values for each IF regioisomer. Our results show that the conductance of the diradical **IFS** is much higher than that found for the purely CS **IFA** and the other analogues (**IFNS** and **DH-IFS**). In order to corroborate the experimental results and gain a more fundamental understanding of why this occurs, we have performed gas-phase and single-molecule junction transmission simulations based on density functional theory (DFT), and nonequilibrium Green’s function (NEGF) formalism coupled to DFT, respectively.

## Results and Discussion

**Scheme 1:**
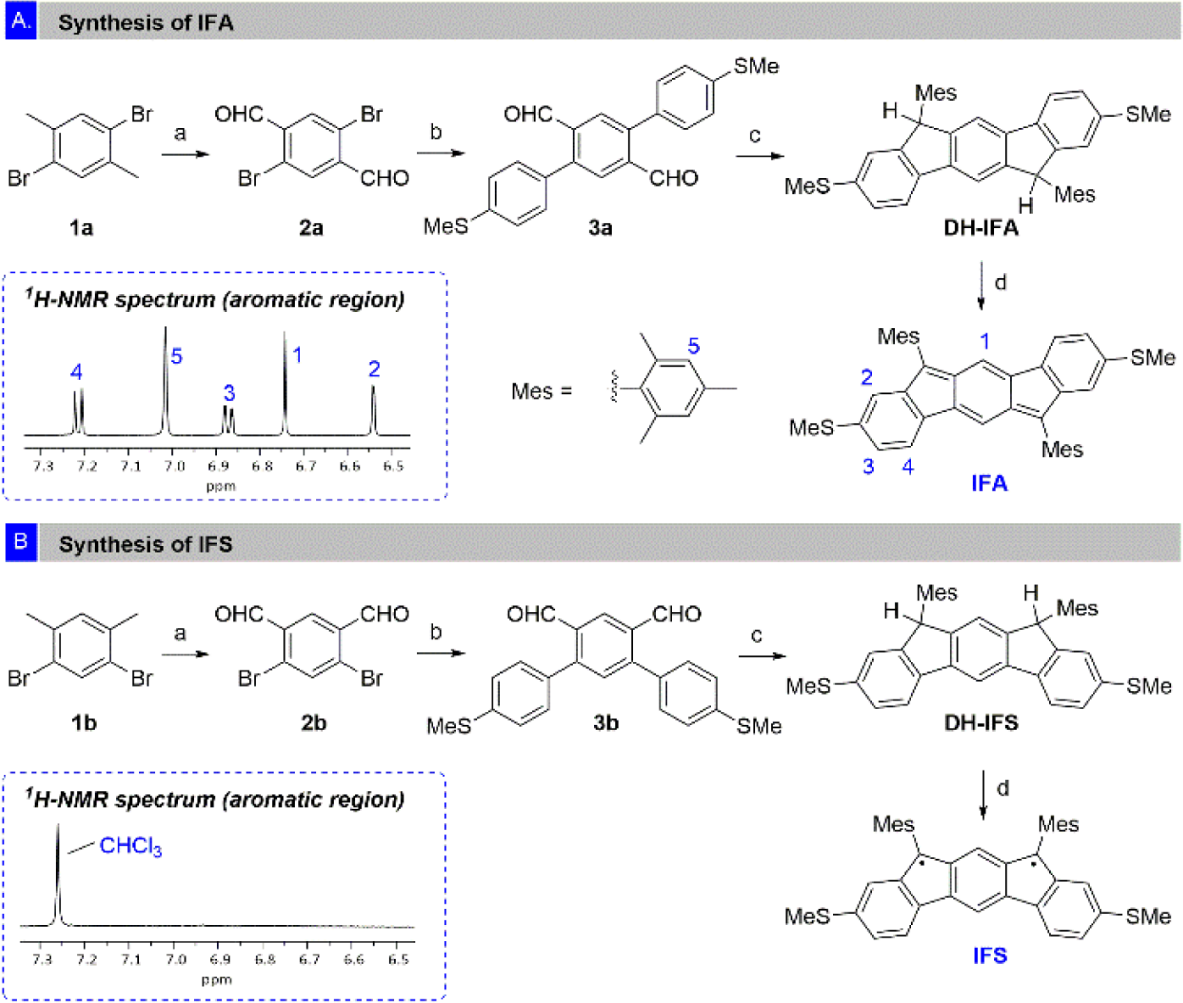
(A) Synthesis of IFA (Inset: Aromatic Region of the ^1^H NMR Spectrum of IFA in CD_2_Cl_2_); (B) Synthesis of IFS (Inset: Aromatic Region of the ^1^H NMR Spectrum of IFS in CDCl_3_) Reaction conditions: (a) (i) CrO_3_, Ac_2_O, AcOH, H_2_SO_4_, 10–15°C, 18 h; (ii) EtOH, H_2_SO_4_, H_2_O, 85°C, 3 h; (b) 4-thiomethylphenyl boronic acid, Pd(OAc)_2_, Bu_4_NBr, K_2_CO_3_, toluene, H_2_O, 70°C, 18 h; (c) (i) MesMgBr, THF, 25°C, 1 h; (ii) BF_3_·OEt_2_, DCM, 25°C, 30 min; (d) DDQ, toluene, 70°C, 3–6 h. See SectionS2 for further details.

We first focus on the preparation and the study of **IFA** and **IFS**. To synthesize both compounds, we followed the reaction sequences shown in the Scheme 1. Starting from the corresponding bromoarenes **1a**/**1b**, we obtained the dialdehydes **2a**/**2b** by selective benzylic oxidation of the methyl groups. Subsequently palladium-catalyzed Suzuki reactions were carried out to give compounds **3a**/**3b**. The corresponding boronic acid bears the SMe-anchor groups necessary for the single-molecule studies. Following this, nucleophilic addition of MesMgBr to the corresponding aldehydes **3a**/**3b**, followed by Friedel–Crafts reactions gave the dihydroindenofluorenes **DH-IFA**/**DH-IFS**. Each compound was obtained as a mixture of *syn-anti* diastereoisomers (referred to the relative orientation of the Mes groups), which were not separated as it is inconsequential for the outcome of the next reaction. Finally, after oxidative dehydrogenation, using 2,3-dichloro-5,6-dicyano-benzoquinone (DDQ), the corresponding compounds **IFA** and **IFS** were obtained.

**IFA** showed well-defined ^1^H- and ^13^C NMR spectra, confirming its closed-shell configuration (Scheme 1A, inset). In addition, **IFA** was crystallized from hexanes/DCM mixtures, and its structure analyzed by X-ray diffraction (Section S5). As expected, this molecule features the distinct *p*-QDM unit within the core, with alternating long (1.436 and 1.470 Å) and short (1.380 and 1.361 Å) C–C bond lengths, in good agreement with the reported structure without -SMe groups.^20c^ On the other hand, **IFS** was NMR silent at room temperature (Scheme 1B, inset), which is consistent with an open-shell configuration in which the paramagnetic triplet state is thermally accessible. This was corroborated by electron paramagnetic resonance (EPR) in which we could observe the signal for the Δm_s_= ± 2 transition characteristic of the triplet state (Figure S27). These results are in line with the observations reported by Shimizu et al.^22a^ for the structure without anchoring groups. Finally, for **IFS**, the exact mass and isotopic distribution pattern of the peak, corresponding to the [M]^+^ species in the high-resolution mass spectrum, fits very well with the theoretical data (Figure S25). We also checked the general stability of each compound. **IFA** was stable for months under ambient conditions. In the case of the open-shell diradical **IFS**, the compound was reasonably stable up to 1 week when kept under an argon atmosphere at −20 °C. In terms of its stability in solution under ambient conditions, we found its half-life to be ∼38 h. This lifetime decreases down to ∼1 h if the sample is further manipulated (i.e., evaporation/redissolution cycles). Remarkably, and despite its inherent instability, this lifetime proved to be enough to perform BJ experiments. In agreement with these experimental results, our (U)DFT calculations predict that the electronic ground state of **IFA** corresponds to a singlet closed-shell (CS) structure, whereas a singlet open-shell (OS) structure is the most energetically stable configuration for **IFS**, with a low-lying triplet state at only 1.10 kcal/mol (0.048 eV) (Table S2). For a comprehensive description about the computational methods employed and related results, please refer to the Supporting Information.

As the diradicaloid character is related to the singlet–triplet energy gap (Δ*E*_S-T_) and, concomitantly, to the HOMO–LUMO gap (*E*_gap_), optoelectronic properties of both compounds have also been examined (see Section S7 and S8). The UV–Vis absorption for **IFA** (Figure S28) showed a low-energy band with an absorption maximum at 521 nm, which corresponds to an optical gap (*E*_gap_) of 2.23 eV, similar to that observed for the molecule without -SMe groups previously reported (*E*_gap_ = 2.29 eV).^20c^ In the case of **IFS** (Figure S29), we observed a band centered at λ_max_ = 653 nm and a very weak band starting at ∼930 nm spreading until the detection limit of our spectrophotometer (∼1100 nm) (Figure S30).^27^ From the cyclic voltammetry we estimated an electrochemical HOMO–LUMO gap of 1.89 eV for **IFA** and 1.15 eV for **IFS**. Likewise, DFT calculations predict a HOMO–LUMO gap of 2.34 eV in **IFA**, and a much lower gap of 1.23 eV for **IFS.** The same trend is observed for Δ*E*_S-T_, the value for **IFS** being more than 15 kcal/mol (0.76 eV) lower than for **IFA** (see Section S10.1). This open shell diradicaloid behavior of **IFS** is related to the electronic density distribution within their singly occupied molecular orbitals (SOMOs), which exhibits distinct α and β distributions, characteristic of the disjointed nature (Figure S45), and to a spin density distribution mainly located on the bridging carbon atoms of the five membered rings. The density coincides with the protected radical centers, spreading over the external phenyl rings in the OS, and on the central phenyl ring in the T state (Figure S46). This spin distribution explains the larger instability observed for this isomer due to spin delocalization onto unprotected positions. Finally, we have quantitatively evaluated the open-shell character by using the single-determinant (U)DFT scheme. For that we calculated the diradical character index (*y*), a theoretical parameter that gives an idea of the contribution of the OS configuration to the overall structure (see Figure 1a).^28^ In the case of **IFA**, a value of *y* = 0 was determined, while **IFS** shows a considerable amount of diradical character (*y* = 0.669), confirming the fact that, in the ground state, the CS structure of **IFA** is the most stable one, whereas a mixture of CS and OS configurations best describes the electronic structure of **IFS**.

Single-molecule conductance experiments were carried out using a home-built STM following the STM-BJ methodology, in air under ambient conditions.^26^ The experiment consists of driving a gold tip in and out of a gold surface covered with target molecules while monitoring the conductance (*G*) as a function of distance (*z*). During retraction, a nanoscale gold bridge forms between the two electrodes, which breaks leaving a small gap into which molecules can bind. If no molecule binds, the tunneling current between the separating electrodes decreases exponentially. When a molecule binds, however, a plateau in *G* is seen as the electrodes are separated (examples given in Figure S34). Further details on the procedure can be found in Section S9. We recorded thousands of *G-z* traces (at least 3 independent runs with between 8000 and 20 000 traces per run) which were analyzed with an automated algorithm to separate the plateau-containing traces from those with only the tunneling background. 1D and 2D histograms were built accordingly, in which peaks (1D) or clouds (2D) form in the regions where plateaus repeatedly appear, allowing us to extract the characteristic molecular conductance values and stretching lengths.

**Figure 3:**
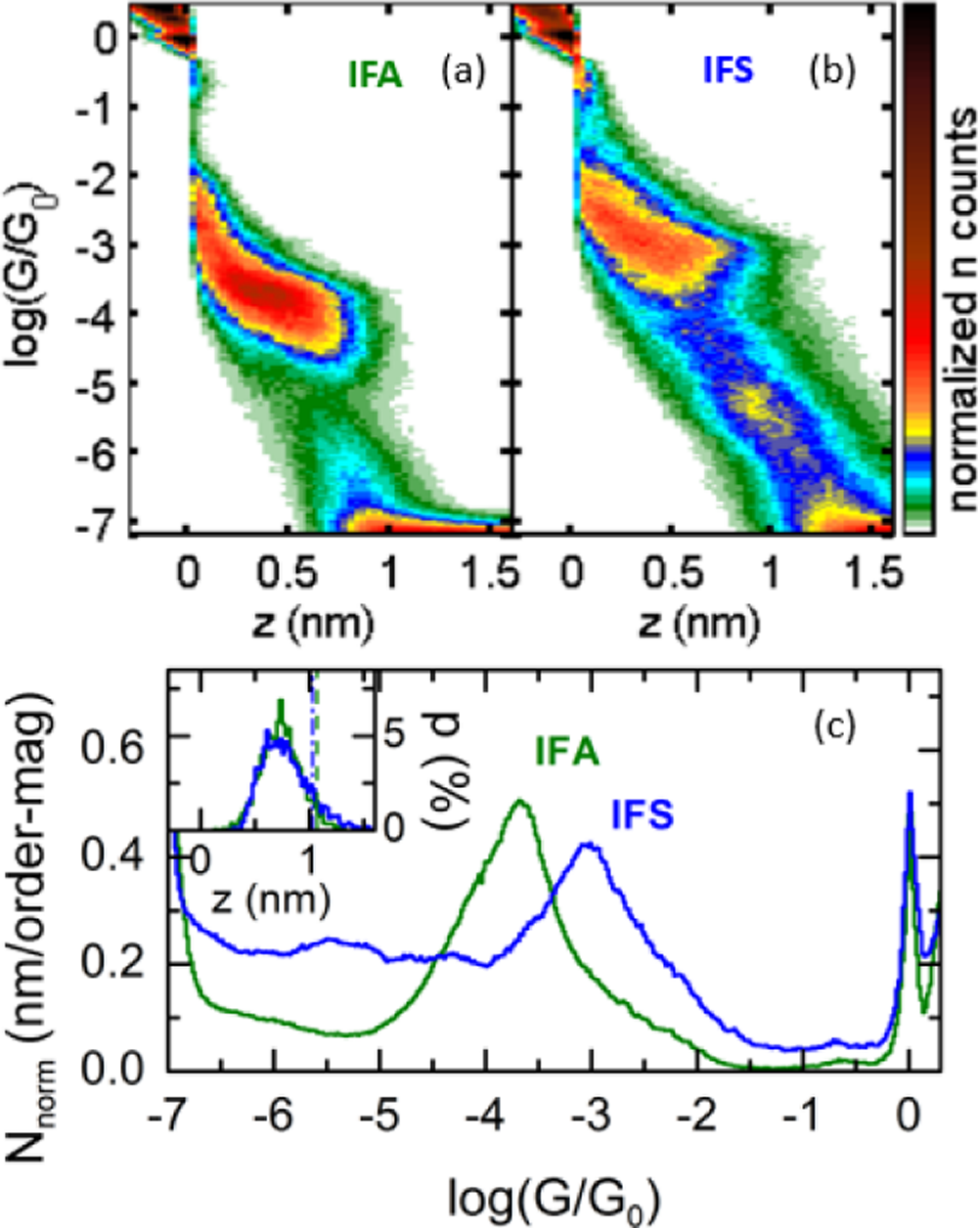
(a) 2D-histogram of **IFA** built from all traces presenting plateaus; (b) 2D-histogram of **IFS** built from all traces presenting plateaus; (c) 1D-histograms of **IFA** and **IFS**. Inset: plateau length distributions, vertical dashed lines indicate the theoretical maximum plateau length expected for **IFA** (green) and **IFS** (blue). For both compounds, the histograms were built with above 3300 traces.

The single-molecule conductance results of **IFA** and **IFS** are shown in Figure 3. For **IFA**, we identified plateaus in 19% of the *G-z* traces, which were therefore used in the analysis. The 2D histogram in Figure 3a displays a pronounced and clear plateau region between approximately log(*G*/*G*_*0*_)= −3 and −4.5. By fitting a Gaussian to the conductance peak in the 1D histogram (green trace, Figure 3c), we determined the most-probable conductance of **IFA** to be log(*G*/*G*_*0*_)= −3.8, with a half width at half-maximum (HWHM) of 0.4. **IFA** is structurally similar to a dihydroindenofluorene bearing SMe groups (**DHIF-SMe**) which has been previously studied in single-molecule experiments.^29^**DHIF-SMe** is very similar to the **IFA** precursor **DH-IFA**, but having H instead of Mes groups in its structure. The reported conductance of **DHIF-SMe** is log(*G*/*G*_*0*_)= −3.1,^29^ which is higher than that of **IFA** (see also Table S1). For **DHIF-SMe**, it is possible to draw resonance structures connecting the two S atoms using curly arrows (Figure S35b), which, according to the methodology described by O’Driscoll and Bryce (curly arrow rules, CARs) suggests that transport takes place via constructive quantum interference (CQI).^30^ The *p*-quinodimethane at the core of **IFA**, on the other hand, leads to a lack of direct conjugation (Figure S35a, top), which should give rise to destructive quantum interference (DQI)^31,−33^ and provides a reason for the observed lower conductance. This also confirms the negligible presence of the diradical resonance form of **IFA** (Figure S35a, down), since in OS **IFA** it would, in principle, be possible to have direct conjugation between S atoms due to the presence of three Clar sextets rather than two. It is interesting to note, however, that despite this conjugation break, the conductance of **IFA** is not dramatically lower than **DHIF-SMe**.

We next discuss the single-molecule conductance results of **IFS**. The experiments were challenging due to the inherent instability of this diradical under ambient conditions. In order to avoid significant decomposition during the measurements, we performed the break-junction experiments using substrates exposed to freshly synthesized **IFS**. In this way, we found a highly reproducible signal, with plateaus in 25–45% of the recorded traces in different experimental runs. The 2D histogram (Figure 3b) showed a prominent peak centered at log(*G*/*G*_0_)= −3.0 with a HWHM of 0.4. In the Supporting Information we give details about the minority low conductance signals contributing to the histogram below log(*G*/*G*_0_)= −4, which most probably results from partial decomposition of **IFS**. The evolution of the STM-BJ plateaus of **IFS** over time is shown in addition in Figure S42. The most probable conductance of **IFS** can be compared to that of CS **IFA**, the former being about 1 order of magnitude higher than the latter. More precisely, we observe an increase in conductance of approximately 800% with respect to **IFA**. Meanwhile, the plateau length distributions for both compounds overlap, as shown in the inset of Figure 3c. As is typical for -SMe terminated compounds,^34^ the tail of their distributions extends up to the theoretical molecular length (corrected for the initial jump-out-of-contact, JOOC) shown by vertical dashed lines in the figure. This length was obtained from the S–S length of each molecule subtracting a JOOC distance of 0.4 nm, typical of gold contacts. Therefore, these results confirmed the S–S anchoring in both compounds.

In addition, we can compare **IFS** with **DHIF-SMe,** previously studied in the literature.^29^ Both **IFS** and **DHIF-SMe** display similarly high conductances around log(*G*/*G*_0_)= −3. Both are planar compounds with three six-membered rings. However, in **DHIF-SMe** each ring is formally an aromatic phenyl and the SMe groups are connected *para* to one another. On the other hand, **IFS** has a diradical character of *y* = 0.669, meaning that it can be thought of a mixture of resonance isomers (as depicted in Figure 1B, bottom). Neither resonance form leads to direct formal conjugation between the SMe anchor groups (Figure S36a). Crucially, however, **IFS** exhibits two key differences: one, a very small HOMO–LUMO gap, and two, the existence of resonance structures connecting the radicals located on the bridging carbon atoms with the SMe groups (Figure S36b). It has been shown that compounds with anchor groups formally out of conjugation with each other can, nevertheless, lead to a high conductance when side groups or heteroatoms are placed at specific positions along the backbone.^31a,32c,35,36^ O’Driscoll and Bryce showed that, if it is possible to draw resonance structures connecting these groups with the anchor groups (or more specifically two acceptor groups placed at the sites of the original anchor groups), then the energetic position of the destructive interference, which would otherwise dominate at the center of the HOMO–LUMO gap, can be shifted to higher/lower energies (shifted-DQI, s-DQI).^30^ Typically, this has a positive influence on the conductance.^37^ Here, we suggest that the radicals in **IFS** may play a similar role in alleviating DQI, which, based on its main resonance isomer, may otherwise be expected to dominate the transport behavior.

To further check if the diradical nature of **IFS** is involved in the high conductance of the molecule, we prepared a structurally related closed-shell compound, **IFSN** (the synthesis of **IFSN** is shown in Scheme S3). This compound has the same ring-fusion mode as **IFS**, with the anchor groups located in the same relative positions, but the apical carbon atoms of the five-membered rings have been replaced by nitrogen atoms (Figure 2, left). In this way we have a purely aromatic compound (22 π-electrons), that formally could be described as a reduced version of **IFS**. Additionally, we also measured compound **DH-IFS**, the synthetic precursor of **IFS**, which contains an sp^3^ saturated carbon at the bridge locations (Figure 2, right).

**Figure 4:**
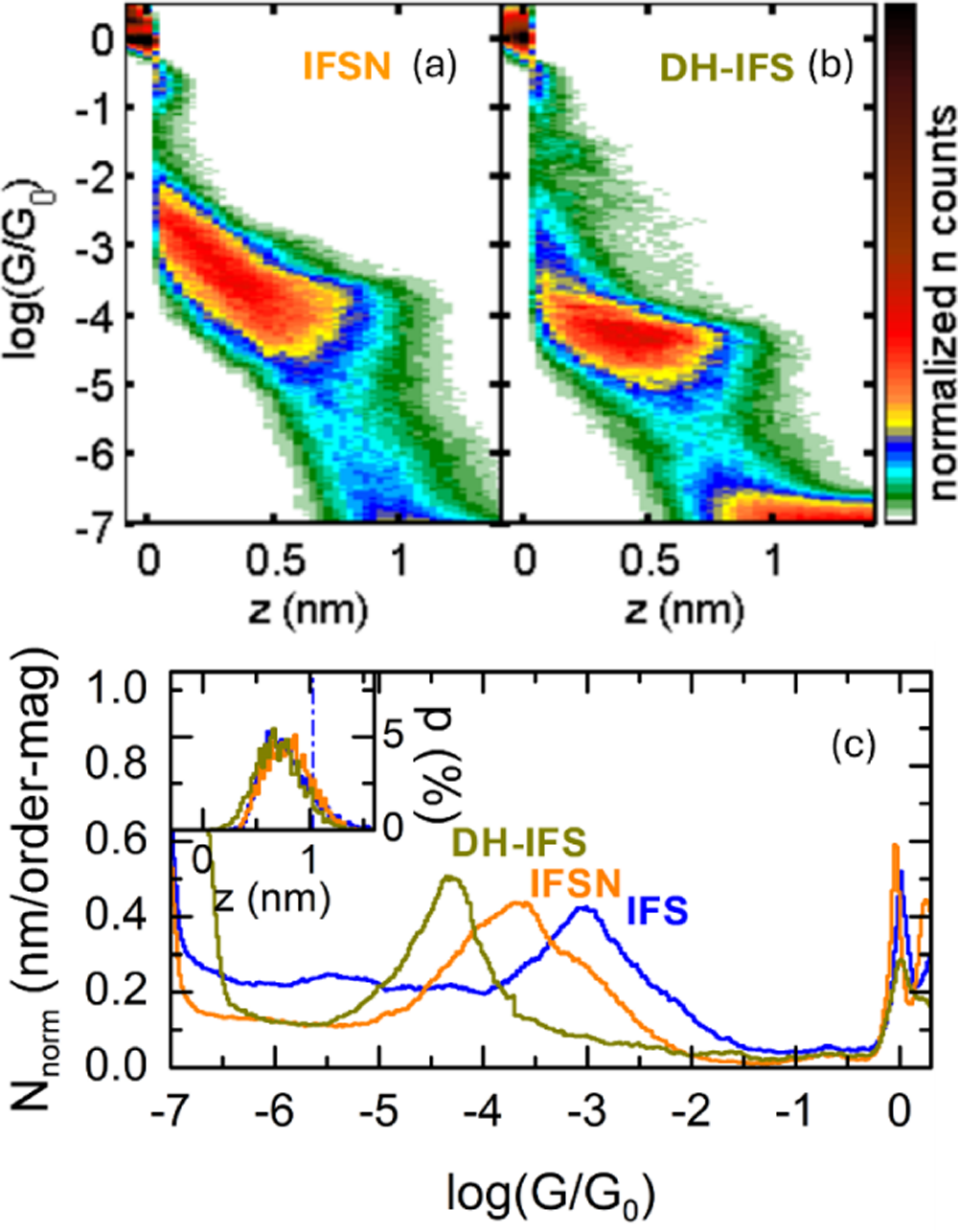
(a) 2D-histogram of **IFSN** built from all traces presenting plateaus; (b) 2D-histogram of **DH-IFS** built from all traces presenting plateaus; (c) 1D-histograms of **IFSN**, **DH-IFS**, and **IFS**. Inset: plateau length distributions, vertical dashed lines indicate the theoretically expected maximum plateau length.

The break-junction results of **IFSN** and **DH-IFS** are shown in Figure 4. Plateaus were identified in 30% and 27% of the *G-z* traces, respectively. The shape of the 2D histograms for **IFSN** (Figure 4a) and **DH-IFS** (Figure 4b) are very similar to that of **IFS**, with the distributions tapering to a point. This can be interpreted as an indication that similar binding geometries occur for the three compounds, which reinforces the idea that the conductance differences arise from the core of the molecules and not from different binding configurations. By fitting a Gaussian to the conductance peak in the 1D histograms (Figure 4c), the most probable conductance of **IFSN** is found to be log(*G*/*G*_*0*_)= −3.7, with a HWHM of 0.6. This value is in the range of that found for **IFA** but, more importantly, is close to 1 order of magnitude lower than that for the structurally related **IFS** (also shown in Figure 4c). In **IFSN** there is no path that directly connects both anchor groups according to CARs (see Figure S37a) which means, *a priori*, DQI should dominate around the Fermi level. Although, the presence of heteroatoms can, as previously mentioned, alleviate DQI (Figure S37b), the nitrogen lone pair in **IFSN** is not very well conjugated into the π-system, and so we expect this effect to be somewhat diminished. The more delocalized radicals in **IFS** better couple to the π-system which, we suggest, leads to a stronger shifted-DQI effect and thus higher conductance. The results for **DH-IFS**, a molecule with no heteroatoms or radicals in the polycyclic structure, further support our hypothesis. As can be seen in Figure 4b, **DH-IFS** displays a single conductance group at log(*G/G*_0_)= −4.3 with a HWHM of 0.4. This value is much lower than that for **IFNS** and **IFS** (approximately half an order of magnitude and 1 order of magnitude, respectively), due to the lack of a conjugation pathway connecting both anchor groups (Figure S38). Therefore, the trend in conductance values observed for this set of molecules (log(*G/G*_*0*_): **IFS** > **IFSN** > **DH-IFS**) is consistent with both lone pairs and unpaired electrons being able to alleviate the DQI that would otherwise dominate the low-bias conductance. Further, it suggests the higher efficiency of unpaired electrons in doing so for this structural case, leading to higher conductance values.

To shed additional light on the experimental results, first-principles elastic transport simulations based on NEGF-DFT were carried out within the Landauer formalism,^38^ according to which, in the linear regime, the conductance is approximately given by the transmission at the Fermi level: *G*= *G*_*0*_ T(E_F_), with *G*_*0*_ being the quantum conductance. First, gold-molecule-gold junction geometries were optimized using the SIESTA code.^39^ To ensure an accurate comparison, the molecules were connected to gold with the same end-to-end arrangement in all cases (Figure S47). Then, the TranSIESTA package and the postprocessing TBTrans tool^40^ were employed to obtain the zero-bias electron transmission probability function, T(E). Due to the diradical nature of **IFS**, spin-polarized calculations were performed on the different spin states, whereas spin-unpolarized were carried out on **IFA, IFSN** and **DH-IFS**. See Section S10, for a comprehensive description of the whole computational procedure.

**Figure 5:**
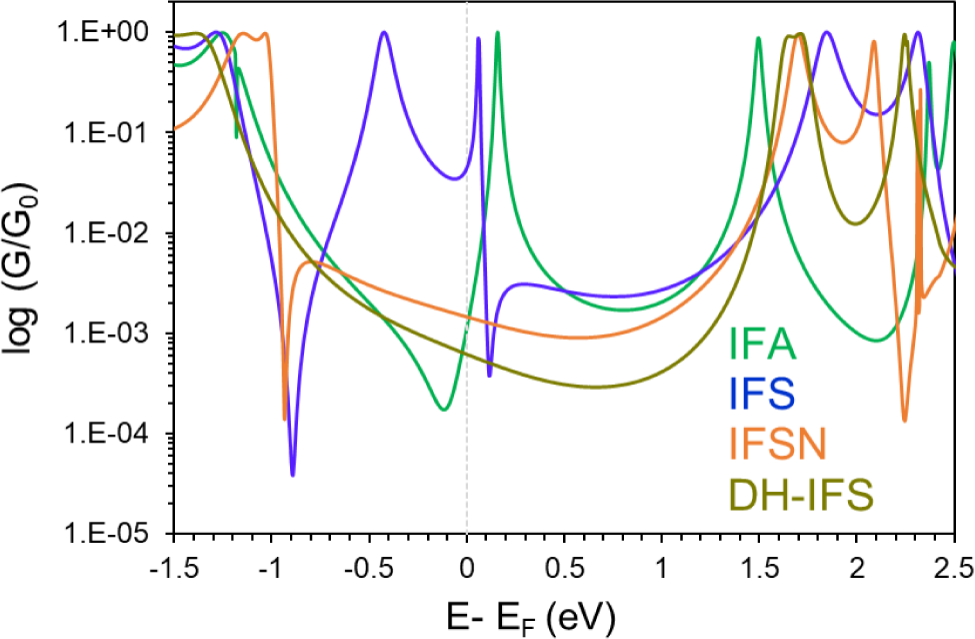
Zero-bias transmission spectrum for IFA (green), IFSN (orange), DH-IFS-*syn* (olive), and the averaged spin-up and spin-down transmission coefficient of IFS OS (blue).

Figure 5 presents a comparison of the total transmission spectra for the electronic ground state of **IFA**, **IFS**, **IFSN** and **DH-IFS-syn**, as estimated by DFT. These refer, respectively, to the transmission of CS **IFA**, **IFSN** and **DH-IFS-syn**, and to the average spin-up and spin-down transmissions (T^σ^(E)) of OS **IFS**. Given its proximity in energy to the OS ground electronic state (as estimated theoretically), the transmission function for **IFS** in the T state was also computed (see Table S2). Spin-dependent and total-transmission coefficients for OS, and T states are shown in Figure S48. It should be noted that the total transmission curves for open-shell configurations are associated with a distribution of spin densities analogous to those found for molecules in the gas phase, as can be confirmed by comparison between Figures S46 and S49. Furthermore, the transmission curves of the two diastereoisomers (*syn*/*anti*) of **DH-IFS** are nearly indistinguishable, as detailed in Section S10.2.2.

As shown in Figure 5, the transmission is characterized by interference phenomena in the vicinity of the Fermi level (E_F_) in both **IFS** and **IFA** (see Section S10.2.1 for more details), leading to near-resonant transport via the tail of the LUMO. In contrast, the wider energy gap in **IFSN** and **DH-IFS**, results in a nonresonant transport mechanism, even though the tunneling distance is practically the same. The computed conductance values, in units of log (*G*/*G*_0_), are −2.94 for **IFA**, –1.35 for **IFS-OS**, –1.18 for **IFS-T**, –2.83 for **IFSN**, and −3.20 for **DH-IFS**. Therefore, the higher conductance of **IFS** compared to **IFA**, **IFSN** and **DH-IFS**, is independent of **IFS** being in the OS or T states, and is likely related to its smaller energy gap as a consequence of its open-shell character.

To better understand the role of the unpaired electrons in conductance, a description of the interference signals is required. The final transmission in **IFA** is influenced by a destructive antiresonance dip close to E_F_ (−0.12 eV), associated with the lack of direct conjugation between anchors, as detailed in the experimental section (see Section S10.2.1 for a detailed description). Conversely, **IFS-OS** exhibits a Fano-resonance adjacent to E_F_ with a width of only 0.1 eV, along with a sharp antiresonance located too far from E_F_ to efficiently contribute to the conductance. Additionally, **IFSN** displays a DQI feature situated close to the HOMO energy which, considering its structure, is consistent with the idea of s-DQI based on the CARs. This supports the experimental conclusion that unpaired electrons from radicals are more effective in alleviating DQI within the HOMO–LUMO gap than the lone pairs of nitrogen. Finally, no strong DQI feature was found in the transmission spectrum of **DH-IFS**. Although, based on curly arrow principles, we would expect destructive quantum interference to dominate inside the HOMO–LUMO gap, the absence of such interference may be explained by the existence of a lower-lying sigma channel that dominates the conductance, as previously reported for shorter molecules.^41^

Overall, our theoretical results qualitatively align with the experimentally observed trends, providing a solid basis for their interpretation. Given, however, the low first oxidation wave of **IFS** (Figure S33), one cannot completely discard that the diradical was oxidized during the break-junction experiment, giving rise to other open-shell species. We have explored this possibility by further calculating the transmission spectrum of the **IFS** radical cation (details in Section S10.2.3). This structure, as for the neutral diradical, shows a similarly high transmission (log (*G*/*G*_*0*_)= −1.71) with respect to **IFA**, **IFSN** and **DH-IFS**. However, the radical cation would itself be a reactive species due to its positive charge which, in the absence of a polarizable medium, would most likely lead to further reactions with, for example, the ambient water present on the surface. This would thus lead to species similar to **DH-IFS**, which, as mentioned above, may account for low-conductance plateaus in the signal of **IFS** (see Section S9.4). Other compounds with low oxidation potentials, such as porphyrins, also tend to form charged states that are stable for at most a few seconds, and typically a lot less, resulting in strong conductance fluctuations on the time scale of the plateau measurement.^42^ For **IFS**, the plateaus do not exhibit this type of behavior, thus suggesting that any oxidation occurring in the break junction results in the formation of other neutral species with much lower conductance than the diradical. This reinforces our interpretation that the neutral diradical (in resonance with the quinoid configuration) is responsible for the high conductance observed for **IFS**.

## Conclusions

We report the first single-molecule conductance measurements of a neutral open-shell diradical based on an indeno[2,1-*b*]fluorene core (**IFS**). Using a combination of the STM-BJ technique along with NEGF-DFT theoretical simulations, we show that the presence of unpaired electrons facilitates conductance across **IFS** compared to that of the closed-shell indeno[1,2-*b*]fluorene isomer **IFA**. For **IFA**, the measured conductance of log *G*/*G*_*o*_ = −3.8 is related to the widening of the energy gap and to destructive interference resulting from the lack of direct conjugation between the thiomethyl anchor groups. On the other hand, the open-shell **IFS** displays a significantly higher conductance of log *G*/*G*_*o*_ = −3.0, despite none of the formal resonance structures having direct conjugation between the anchor groups. The high conductance can be, at least partially, attributed to its small HOMO–LUMO gap. Further, we suggest that the lack of DQI in the HOMO–LUMO gap, which would be predicted based on simple “curly arrow” rules, can be understood in terms of the efficient coupling of the radicals with the π-system, which, as we have shown for compounds containing heteroatoms, alleviates the potential appearance of DQI. Comparison with the structurally related aromatic **IFSN** along with the CS dihydro precursor to **IFS** (**DH-IFS**) supports this idea. **DH-IFS** has the lowest conductance, probably dominated by transport via the σ bond channel. **IFSN**, on the other hand, sits between **DH-IFS** and **IFS**. The nitrogen lone pairs lie out of plane with the π-system and the DQI is alleviated, but less efficiently than in **IFS**.

Our calculations support the break-junction results by matching the trend in conductance found at the Fermi level. An antiresonance occurs within the HOMO–LUMO gap for **IFA**, in line with the lack of direct S–S conjugation. On the contrary, no antiresonance is found for **IFS** within its HOMO–LUMO gap. Instead, we find a Fano resonance at the position of the LUMO. Due to the much smaller overall energy gap, the calculated transmission is, therefore, larger than for **IFA**.

This work showcases the behavior of an open-shell diradical when incorporated to single-molecule junctions and the remarkable difference in conductance with respect to other compounds in the IF family. We hope that these results, which demonstrate that open-shell species can form stable molecular junctions under ambient conditions, may encourage the investigation of single-molecule magnetism at room temperature, particularly, via singlet–triplet excitation.

## Supporting Information Available

Synthetic procedures, NMR spectra, high-resolution mass spectra, UV–vis spectra, voltammograms, EPR spectrum, STM-BJ experimental details and results, theoretical calculation methods and results (PDF)
