# Supplementary material for: Single-Molecule Conductance of Neutral Closed-Shell and Open-Shell Diradical Indenofluorenes
Source: J Am Chem Soc. 2024 Oct 18;146(43):29977–86. doi: 10.1021/jacs.4c13551 (PMC11528439; doi:10.1021/jacs.4c13551)
Supplement: Supplementary file 1 — ja4c13551_si_001.pdf [file ja4c13551_si_001.pdf]

SUPPORTING INFORMATION

Single-Molecule Conductance of Neutral Closed-Shell and Open-Shell Diradical Indenofluorenes

Raquel Casares,[a,†] Sandra Rodríguez-González,[b,†]* Álvaro Martínez-Pinel,[a‡] Irene R. Márquez,[a,c,‡] M. Teresa González,[d,‡] Cristina Díaz,[e] Fernando Martín,[d,f] Juan M. Cuerva,[a]* Edmund Leary,[d]* Alba Millán[a]*

[a] Departamento de Química Orgánica, Unidad de Excelencia de Química Aplicada a Biomedicina y Medioambiente (UEQ), C. U. Fuentenueva, Universidad de Granada, 18071 Granada, Spain.

[b] Departamento de Química Física Aplicada, Universidad Autónoma de Madrid, 28049 Madrid, Spain.

[c] Centro de Instrumentación Científica, Universidad de Granada, 18071 Granada, Spain.

[d] Fundación IMDEA Nanociencia, 28049 Madrid, Spain.

[e] Departamento de Química Física, Facultad de Ciencias Químicas, Universidad Complutense de Madrid, 28040 Madrid, Spain.

[f] Departamento de Química, Módulo 13, Universidad Autónoma de Madrid, 28049 Madrid, Spain.

*emails: sandra.rodriguez@uam.es; jmcuerva@ugr.es; edmund.leary@imdea.org; amillan@ugr.es.

Table of Contents

1. **General details** S3
2. **Synthetic procedures for the preparation of IFA, IFS and IFSN** S3
3. **NMR spectra of new compounds** S9
4. **High-resolution mass spectra. Isotopic distribution** S21
5. **Single-crystal X-ray diffraction of IFA** S23
6. **EPR spectrum for IFS** S24
7. **UV–Vis spectra** S25
8. **Voltammograms** S27
9. **STM-BJ experiments** S28
   - 9.1. Molecules for comparison S30
   - 9.2. Depiction of curly-arrow rules (CAR) S31
   - 9.3. Low conductance signals and time evolution in the break junctions experiments of **IFS** S34
   - 9.4. Identification of the low G signal. Comparison with **DH-IFS** signal S36

10 **Theoretical calculation methods and results** S38
   - 10.1. Gas phase quantum chemical simulations S38
   - 10.2. First-principles electron transport calculations S40
   - 10.2.1 Description of the interference phenomena in the transmission functions of **IFA**, **IFS-OS** and **IFSN** S42
   - 10.2.2. Energy-resolved transmission spectra for **DH-IFS** diastereoisomers S43
   - 10.2.3. **IFS** radical cation energy-resolved transmission function S43
   - 10.3. Optimized geometries coordinates S45

11 **References** S60

1. General details

Unless otherwise stated, all reagents and solvents (DMF, DDQ, acetonitrile, CH_2Cl_2, EtOAc, Hexane, MeOH, toluene, Et_2O) were purchased from commercial sources and used without further purification. Anhydrous THF was freshly distilled over Na/benzophenone. 1,4-dibromo-2,5-dimethylbenzene (**1a**), 1,5-bromo-2,4- dimethylbenzene (**1b**) and 1,3-dibromobenzene (**5**) are commercially available. Flash column chromatography was carried out using silica gel (40-63 µm) or neutral alumina as the stationary phase. Analytical TLC was performed on aluminum sheets coated with silica gel with fluorescent indicator UV254 (Alugram SIL G/UV254, Mackerey-Nagel, Germany) and observed under UV light (254 nm) and/or stained with phosphomolybdic acid (5% methanol solution). In general, ^{1}H- and ^{13}C-NMR spectra were recorded on Bruker Avance Neo (400 MHz or 500 MHz) spectrometers at a constant temperature of 298 K. Chemical shifts are reported in ppm and referenced to residual solvent. Coupling constants (*J*) are reported in Hertz (Hz). Multiplicities are abbreviated as follow: s = singlet, br s = broad singlet, d = doublet, t =triplet m = multiplet, dd = doublet of doublets, td = triplet of doublets, ddd = doublet of doublet of doublets, dt = doublet of triplets. Assignment of the ^{13}C-NMR multiplicities was accomplished by DEPT techniques. HRMS spectra were obtained using ESI-TOF or GC-EI techniques. IR-ATR spectra were recorded on a Perkin Elmer Spectrum Two IR Spectrometer.

2. Synthetic procedures for the preparation of IFA, IFS and IFSN

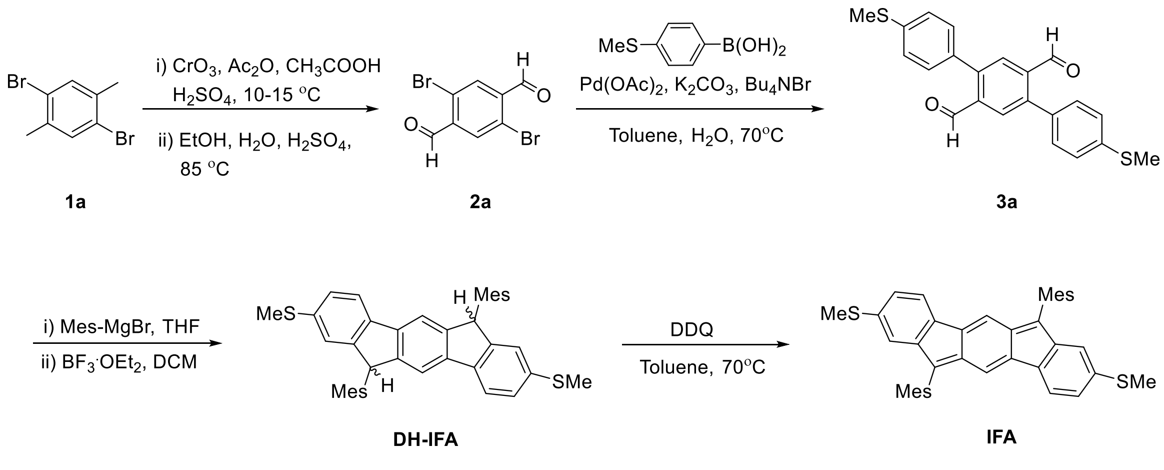

Scheme S1. Synthetic route for compound **IFA**

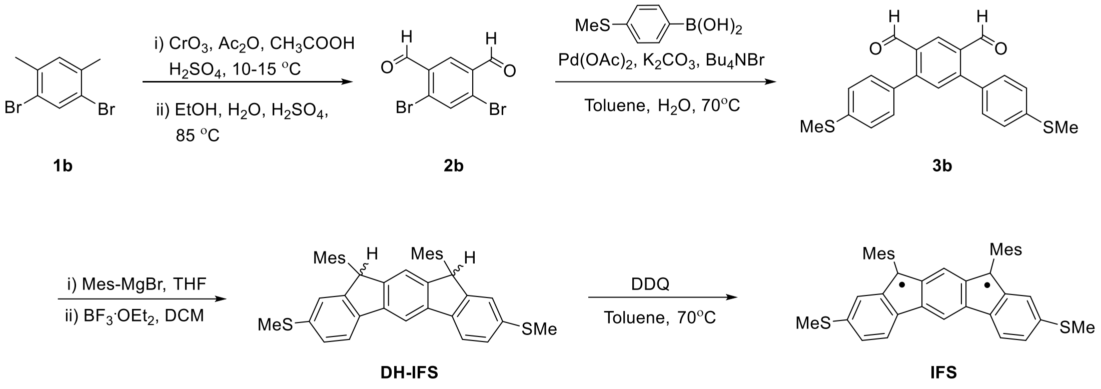

Scheme S2. Synthetic route for compound **IFS**

General procedure for the synthesis of compounds 2a and 2b (GP1)

The corresponding starting material **1a** or **1b** (1 g, 3.78 mmol, 1 equiv.) was dissolved in a mixture of acetic acid (5 mL), acetic anhydride (10 mL), and sulfuric acid (3.5 mL) with stirring and cooled to 5 °C. Chromium trioxide (0.78 g, 7.80 mmol, 2.05 equiv.) was added slowly (over 1.5 h) to keep the temperature of the mixture between 5 °C and 12 °C. Then the mixture was stirred overnight (10-15°C), poured into ice water and stirred for a further 3 h. The product was filtered off. Then the corresponding 2,5-dibromoterephthalaldehyde tetra-acetate or 2,5-dibromoterephthalaldehyde tetra-acetate was added to a mixture of ethanol (5 mL), water (5 mL), and sulfuric acid (0.5 mL), heated under reflux for 3h and cooled. The product was collected by filtration.

<u>Compound **2a**</u>: 2,5-dibromo-*p*-xylene was reacted according to GP1 to afford **2a** (0.44 g, 40% yield) as a white solid. **^{1}H NMR (500 MHz, $CDCl_3$)** δ (ppm): 10.34 (s, 2H), 8.15 (s, 2H). **^{13}C NMR (126 MHz, $CDCl_3$)** δ (ppm): 189.9 (CH), 137.5 (C), 135.1 (CH), 125.6 (C). **HRMS (ESI)** $C_8H_4O_2Br_2Na$ $[M+Na]^+$ calcd: 312.8653; found: 312.8700. Spectroscopic data agree with those previously reported.[S1]

<u>Compound **2b**</u>: 2,4-dibromo-*m*-xylene was reacted according to GP1 to afford **2b** (0.33 g, 30% yield) as a white solid. **^{1}H NMR (500 MHz, $CDCl_3$)** δ (ppm): 10.31 (s, 2H), 8.38 (s, 1H), 8.03 (s, 1H). **^{13}C NMR (126 MHz, $CDCl_3$)** δ (ppm) 189.5 (CH), 138.8 (CH), 133.2 (C), 132.0 (C), 131.1 (CH). **HRMS (GC-EI)** $C_8H_4O_2Br_2$ $[M]^+$ calcd: 289.8789; found: 289.8727. Spectroscopic data agree with those previously reported.[S2]

General procedure for the synthesis of compounds 3a and 3b (GP2)

Compound **2a** or **2b** (1 equiv.), phenylboronic acid (4 equiv.), Bu_4NBr (2 equiv.), $Pd(AcO)_2$ (0.08 equiv.) were dissolved in degassed dry toluene (0.2 M). In another flask K_2CO_3 (5 equiv.) was dissolved in H_2O and degassed for 10 min. The K_2CO_3 solution was added into the compound

solution and heated at 70 °C with constant stirring overnight. After the completion of reaction, the reaction mixture was washed with water (x2) to remove excess of boronic acid and the mixture was extracted with toluene (x2). The combined organic layers were dried over Na_2SO_4 and the solvent was evaporated. The crude was purified by flash column chromatography (SiO_2, CH_2Cl_2).

<u>Compound **3a**</u>: Compound **2a** (0.44 g, 1.26 mmol), the phenylboronic acid (0.84 g, 5 mmol), Bu_4NBr (0.80 g, 2.52 mmol), $Pd(OAc)_2$ (0.022 g, 0.1 mmol), K_2CO_3 (0.86 g, 6.3 mmol) and H_2O (6.1 mL) were reacted according to GP2 to afford **3a** (0.45 g, 80% yield) as a yellow solid. **^{1}H NMR (500 MHz, $CDCl_3$)** δ (ppm): 10.10 (s, 2H), 8.10 (s, 2H), 7.43-7.35 (m, 8H), 2.59 (s, 6H). **^{13}C NMR (126 MHz, $CDCl_3$)** δ (ppm): 191.5 (CH), 143.8 (C), 140.0 (C), 136.5 (C), 132.8 (C), 130.4 (CH), 130.2 (CH), 126.2 (CH), 15.3 (CH_3). **HRMS (ESI)** $C_{22}H_{18}O_2S_2Na$ $[M+Na]^+$ calcd: 401.0646; found: 401.0641.

<u>Compound **3b**</u>: Compound **2b** (0.33 g, 1.13 mmol), the phenylboronic acid (0.63 g, 4.48 mmol), Bu_4NBr (0.60 g, 1.89 mmol), $Pd(OAc)_2$ (0.016 g, 0.075 mmol), K_2CO_3 (0.64 g, 4.68 mmol) and H_2O (4.53 mL) were reacted according to GP2 to afford **3b** (0.38 g, 90 % yield) as a yellow solid. **^{1}H NMR (500 MHz, $CDCl_3$)** δ (ppm): 10.15 (s, 2H), 8.63 (s, 1H), 7.51 (s, 1H), 7.36 (app. s, 8H), 2.55 (s, 6H). **^{13}C NMR (126 MHz, $CDCl_3$)** δ (ppm): 191.0 (CH), 148.9 (C), 140.5 (C), 133.1 (CH), 132.9 (C), 132.8 (C), 130.2 (CH), 128.7 (CH), 126.1 (CH), 15.3 (CH_3). **HRMS (ESI)** $C_{22}H_{19}O_2S_2$ $[M+H]^+$ calcd: 379.0711; found: 379.0871.

General procedure for the synthesis of compounds DH-IFA and DH-IFS (GP3)

Under Ar, Mes-MgBr (1 M in diethylether) was diluted in deoxygenated THF (1.3 M) and cooled to 0 °C for 30 min. Once cold, the mixture was added dropwise to the corresponding compound **3a** or **3b** (1 equiv.) diluted in deoxygenated THF (0.13 M) and stirred at room temperature for 3 h. The reaction was quenched with water and the reaction mixture was extracted with CH_2Cl_2 (x2). The combined organic layers were dried over Na_2SO_4 and evaporated to dryness. The crude was then redissolved in CH_2Cl_2 (15 M) and degassed with Ar for 10 min. $BF_3 \cdot OEt_2$ (20 equiv.) was added dropwise and the mixture stirred at room temperature for 30 min. The solution was then extracted with CH_2Cl_2 (x2) and the combined organic layers were dried over Na_2SO_4 and evaporated to dryness. The crude was purified by column chromatography (SiO_2, CH_2Cl_2/Hexane, 1/1).

<u>Compound **DH-IFA**</u>: Compound **3a** (0.45 g, 1.18 mmol), Mes-MgBr (1.84 mL, 1.84 mmol), $BF_3 \cdot OEt_2$ (3 mL, 23.6 mmol) and CH_2Cl_2 (40 mL) were reacted according to GP3 to afford **DH-IFA** (0.39 g, 57 % yield) as an orange solid (2:1 mixture of isomers). Data of major isomer are reported. **^{1}H NMR (500 MHz, CD_2Cl_2)** δ (ppm): 7.65 (d, *J* = 8.0, 2H), 7.58 (s, 2H), 7.25 (d, *J* = 8.1 Hz, 2H), 7.10 (s, 4H), 6.69 (s, 2H), 5.57 (s, 2H), 2.75 (s, 6H), 2.46 (s, 6H), 2.31 (s, 6H), 1.19 (s, 3H), 1.13 (s, 3H). **^{13}C NMR (126 MHz, CD_2Cl_2)** δ (ppm): 149.0 (C), 146.9 (C), 140.6 (C), 138.9 (C), 138.4 (C), 138.2 (C), 137.9 (C), 136.9 (C), 134.5 (CH), 131.1 (CH), 129.4 (CH), 125.7 (CH), 122.9 (CH), 120.7 (CH),

116.0 (CH), 50.0 (CH), 22.2 (CH_3), 21.1 (CH_3), 19.1 (CH_3), 16.5(CH_3). **HRMS (ESI)** $C_{40}H_{38}S_2$ $[M]^+$ calcd: 582.2415; found: 582.2410.

Compound **DH-IFS**: Compound **3b** (0.38 g, 1 mmol), Mes-MgBr (1.55 mL, 1.55 mmol), $BF_3{\cdot}OEt_2$ (2.53 mL, 19.9 mmol) and CH_2Cl_2 (34 mL) were reacted according to GP3 to afford **DH-IFS** (0.39 g, 57 % yield) as a yellow solid (1:1 mixture of diasteroisomers). Data of one isomer is reported. **^{1}H NMR (500 MHz, $CDCl_3$)** δ (ppm): 8.18 (s, 1H), 7.83 (d, J = 8.0 Hz, 2H), 7.31 (app d, J = 8.0 Hz, 2H), 7.08 (s, 2H), 7.03 (s, 1H), 6.96 (s, 2H), 6.58 (s, 2H), 5.46 (s, 2H), 2.62 (s, 6H), 2.47 (s, 6H), 2.23 (s, 6H), 1.13 (s, 6H). Data for the mixture of isomers **^{13}C NMR (126 MHz, $CDCl_3$)** δ (ppm): 148.1 (C, both isomers), 146.9 (C, one isomer), 146.8 (C, other isomer), 139.7 (C, one isomer), 139.6 (C, other isomer), 138.5 (C, one isomer), 138.3 (C, other isomer), 137.88 (C, one isomer), 137.83 (C, other isomer), 137.6 (C, one isomer), 137.5 (C, other isomer), 137.1 (C, one isomer), 137.0 (C, other isomer), 136.22 (C, one isomer), 136.19 (C, other isomer), 133.8 (C, one isomer), 133.5 (C, other isomer), 130.6 (CH, one isomer), 130.5 (CH, other isomer), 128.9 (CH, one isomer), 128.8 (CH, other isomer), 125.5 (CH, one isomer), 125.4 (CH, other isomer), 122.8 (CH, one isomer), 122.7 (CH, other isomer), 120.0 (CH, both isomers), 111.1 (CH, one isomer), 110.8 (CH, other isomer), 49.5 (CH, both isomers), 21.9 (CH_3, one isomer), 21.7 (CH_3, other isomer), 20.85 (CH_3, one isomer), 20.81 (CH_3, other isomer), 18.9 (CH_3, one isomer), 18.7 (CH_3, other isomer), 16.5 (CH_3, one isomer), 16.4 (CH_3, other isomer). **HRMS (ESI)** $C_{40}H_{39}S_2$ $[M+H]^+$ calcd: 583.2417; found: 583.2409.

General procedure for the synthesis of compounds IFA and IFS (GP4)

Compound **DH-IFA** or **DH-IFS** was dissolved in degassed dry toluene (0.015 M) and warmed to 70 °C. In another flask, DDQ (2.0 equiv.) was dissolved in degassed dry toluene (0.7 M) and the mixture was added to the corresponding **IFA** or **IFS** solution dropwise. The reaction mixture was stirred at 70 °C until completion. Then, the solvent was evaporated under reduced pressure. The crude material was purified by flash column chromatography (neutral alumina, CH_2Cl_2/Hexane, 1/1, under Ar for **IFS**).

Compound **IFA**: Compound **DH-IFA** (0.39 g, 0.67 mmol), DDQ (0.30 g, 1.34 mmol) were reacted according to GP4 to afford **IFA** (0.155 g, 40 % yield) as a brown solid. In this case the reaction time was 6 h. **^{1}H NMR (500 MHz, CD_2Cl_2)** δ (ppm): 7.21 (d, J = 7.8 Hz, 2H), 7.02 (s, 4H), 6.87 (dd, J = 7.8, 1.8 Hz, 2H), 6.74 (s, 2H), 6.54 (d, J = 1.8 Hz, 2H), 2.37 (s, 12H), 2.15 (s, 12H). **^{13}C NMR (126 MHz, CD_2Cl_2)** δ (ppm): 145.8 (C), 145.7 (C), 139.7 (C), 139.2 (C), 138.5 (C), 137.7 (CH), 136.4 (C), 135.4 (C), 130.2 (C), 129.0 (CH), 125.5 (C), 121.4 (CH), 120.9 (CH), 119.1 (CH), 21.6 (CH_3), 20.8 (CH_3), 16.6 (CH_3). **HRMS (ESI)** $C_{40}H_{36}S_2$ $[M]^+$ calcd: 580.2258; found: 580.2269. **IR ν_{max} (neat)/cm^{-1}:** 2917, 2850, 1738, 1571, 1449, 1432, 1354, 1062, 948, 851, 815, 753, 695.

Compound **IFS**: Compound **DH-IFS** (0.25 g, 0.43 mmol), DDQ (0.20 g, 0.86 mmol) were reacted according to GP4 to afford **IFS** (0.152 g, 61 % yield) as a dark green solid. In this case the reaction

time was 3 h. Compound **IFS** is NMR silent. **HRMS (ESI)** $C_{40}H_{36}S_2$ $[M]^+$ calcd: 580.2258; found: 580.2260.

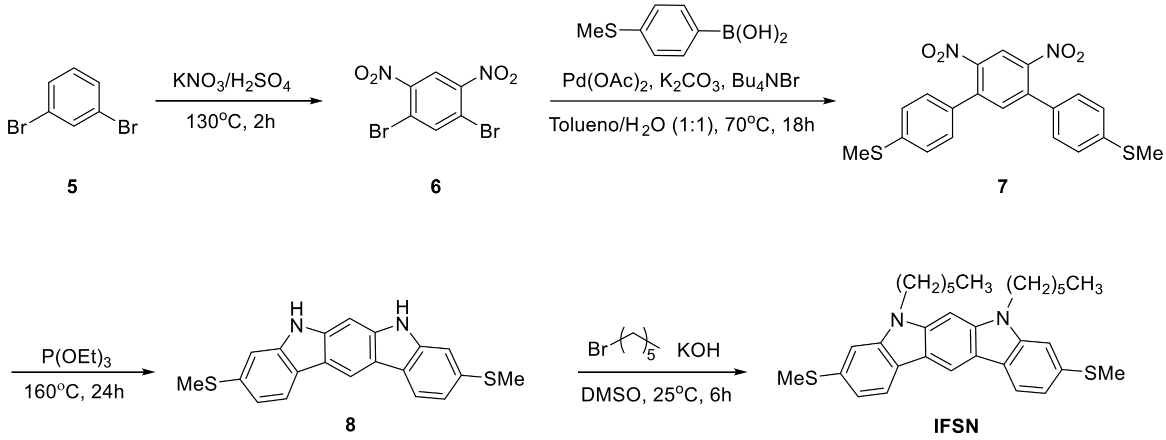

Scheme S3. Synthetic route for compound **IFSN**

Synthetic procedures for the preparation of compound IFSN

<u>Compound **6**</u>: 1,3-Dibromobenzene (**5**) (2.0 g, 8.48 mmol, 1 equiv.) was slowly added to a stirring mixture of concentrated sulfuric acid (6.5 mL, 1.3 M) and potassium nitrate (1.75 g, 17 mmol, 2 equiv.). After the addition was complete, the reaction was stirred at 130°C for 2h. The reaction mixture was then poured onto crushed ice. The yellow solid precipitate formed was collected by filtration, and washed several times with water to give **6** (2.42 g, 88%) as a bright yellow solid. **^{1}H NMR (500 MHz, $CDCl_3$)** δ(ppm): 8.45 (s, 1H), 8.23 (s, 1H). **^{13}C NMR (126 MHz, $CDCl_3$)** δ:141.4 (C), 122.9 (CH), 119.8 (CH), 118.4 (C). **HRMS (ESI)** $C_6H_2N_2O_4Br_2$ $[M]^+$ calcd: 322.8303; found: 322.8300.

<u>Compound **7**</u>: Compound **6** (2.42 g, 7.42 mmol, 1 equiv.), the phenylboronic acid (5 g, 29.70 mmol, 4 equiv.), Bu_4NBr (4.75 g, 14.84 mmol, 2 equiv.), $Pd(AcO)_2$ (0.13 g, 0.60 mmol, 0.08 equiv.) were dissolved in degassed dry toluene (0.8 M). In another flask K_2CO_3 (5.11 g, 37.10 mmol, 5 equiv.) was dissolved in H_2O and degassed for 10 min. The K_2CO_3 solution was added into the compound solution and heated at 70°C with constant stirring overnight. After the completion of reaction, the reaction mixture was washed with water (x2) to remove excess of boronic acid and the mixture was extracted with toluene (x2). The combined organic layers were dried over Na_2SO_4 and the solvent was evaporated. The crude was purified by flash column chromatography (SiO_2, CH_2Cl_2) to give **7** (2.44 g, 88%) as a yellow solid. **^{1}H NMR (500 MHz, $CDCl_3$)** δ(ppm): 8.44 (s, 1H), 7.54 (s, 1H), 7.34 (d, J = 8.3 Hz, 4H), 7.29 (d, J = 8.3 Hz, 4H). **^{13}C NMR (126 MHz, $CDCl_3$)** δ:147.0 (C), 141.1 (C), 139.7 (C), 135.5 (CH), 131.5 (C), 128.1 (CH), 126.3 (CH), 121.0 (CH), 15.2(CH_3). **HRMS (ESI)** $C_{20}H_{16}N_2O_4S_2$ $[M]^+$ calcd: 412.0551; found: 412.0536.

<u>Compound **8**:</u> Compound **7** (100 mg, 0.24 mmol, 1 equiv.) was placed in a flask and deoxygenated with argon. $P(OEt)_3$ (4.6 mL, 0.05 M) was then added and the mixture was left to reflux under

argon for 24h. After the completion of reaction, the reaction mixture was poured onto water ice and a yellow solid precipitated. The solid was filtered and carefully washed with water, DCM and hexane, and then dried to give **8** (35 mg, 42%) as a light yellow solid. **^{1}H NMR (500 MHz, DMSO-d$_6$)** δ(ppm): 11.04 (s, 2H), 8.70 (s, 1H), 8.06 (d, *J* = 8.1 Hz, 2H), 7.37 (s, 1H), 7.32 (s, 1H), 7.08 (d, *J* = 8.3 Hz, 2H), 2.57 (s, 3H). **^{13}C NMR (126 MHz, DMSO-d$_6$)** δ: 141.4 (C), 140.5 (C), 134.0 (C), 121.4 (C), 120.1 (CH), 117.9 (CH), 117.6 (C), 111.1 (CH), 108.5 (CH), 91.5 (CH), 16.3 (CH_3).

<u>Compound **IFSN**</u>: Compound **8** (30 mg, 0.086 mmol, 1 equiv.), KOH (32 mg, 0.86 mmol, 10 equiv.) and DMSO (0.2 mL, 0.4 M) were added to a previously deoxygenated flask. When **7** was completely dissolved, a solution of 1-bromohexane (0.08 mL, 0.86 mmol, 10 equiv.) in DMSO (0.14 mL) was added. The mixture was left at room temperature for 6h. After the completion of reaction, DCM was added and the mixture was washed with water (x2). The organic layer was dried over Na_2SO_4 and the solvent was evaporated. The crude was purified by flash column chromatography (neutral alumina, hexane: EtOAc, 8:2) to give **IFSN** (34 mg, 77%) as a white solid. **^{1}H NMR (500 MHz, CDCl$_3$)** δ(ppm): 8.62 (s, 1H), 8.06 (d, *J* = 6.4 Hz, 2H), 7.32 (s, 2H), 7.20 (dd, *J* = 7.5, 1.5 Hz, 2H), 7.10 (s, 1H), 4.32 (m, 4H), 2.62 (s, 6H), 1.93 (m, 4H), 1.45 (m, 4H), 1.26 (m, 8H), 0.88 (m, 6H). A good quality ^{13}C NMR spectrum could not be obtained due to sample decomposition during long acquisition time in the slightly acid $CDCl_3$ solution. **HRMS (ESI)** $C_{32}H_{40}N_2S_2$ $[M]^+$ calcd: 516.2633; found: 516.2645.

3. NMR spectra of new compounds

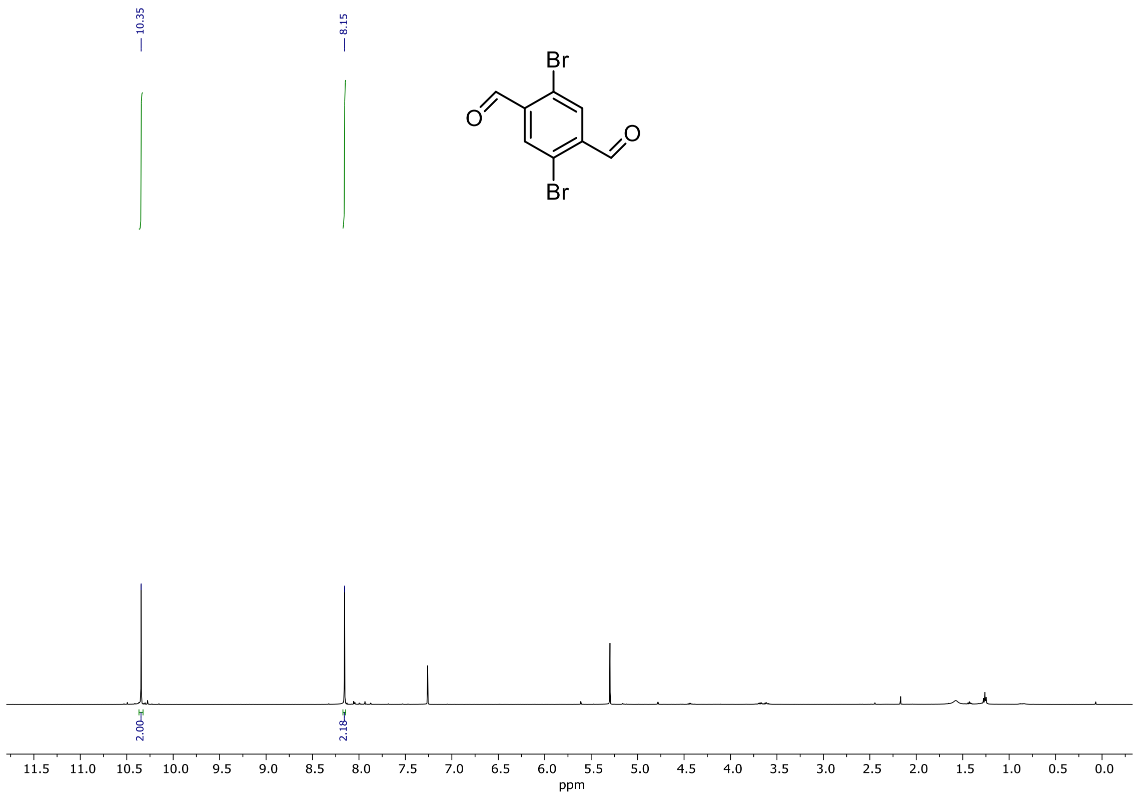

Figure S1. ^{1}H-NMR (500 MHz, $CDCl_3$) of compound **2a**

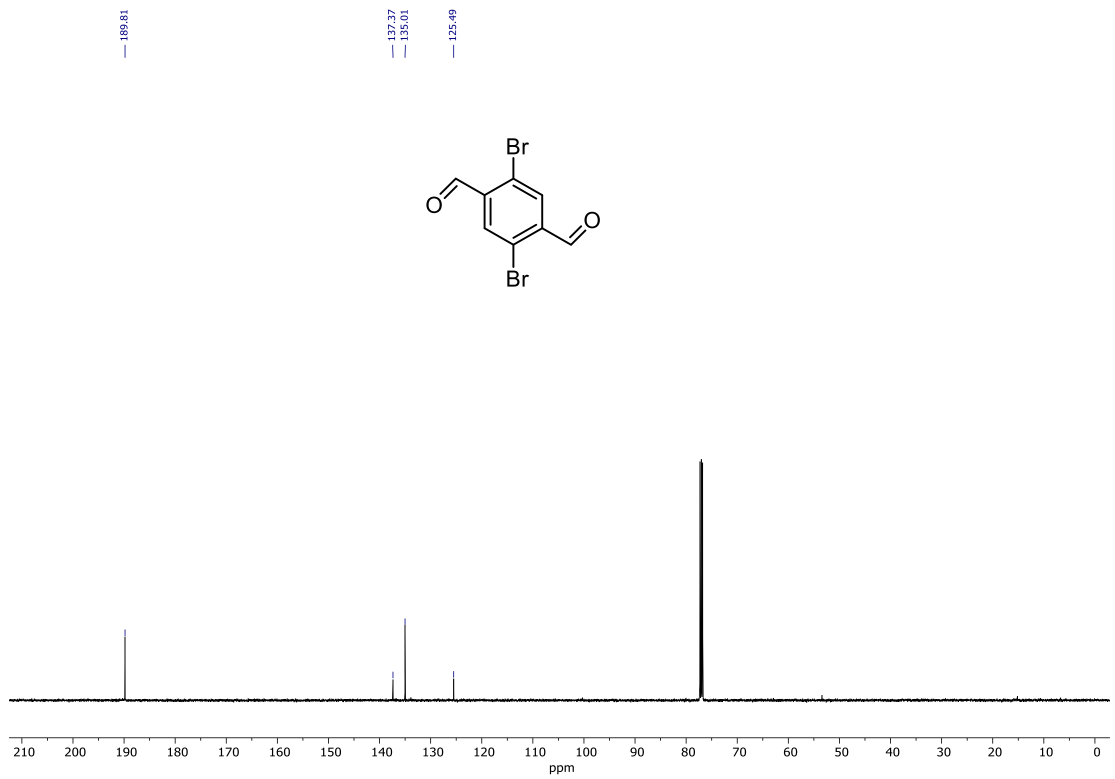

Figure S2. ^{13}C-NMR (126 MHz, $CDCl_3$) of compound **2a**

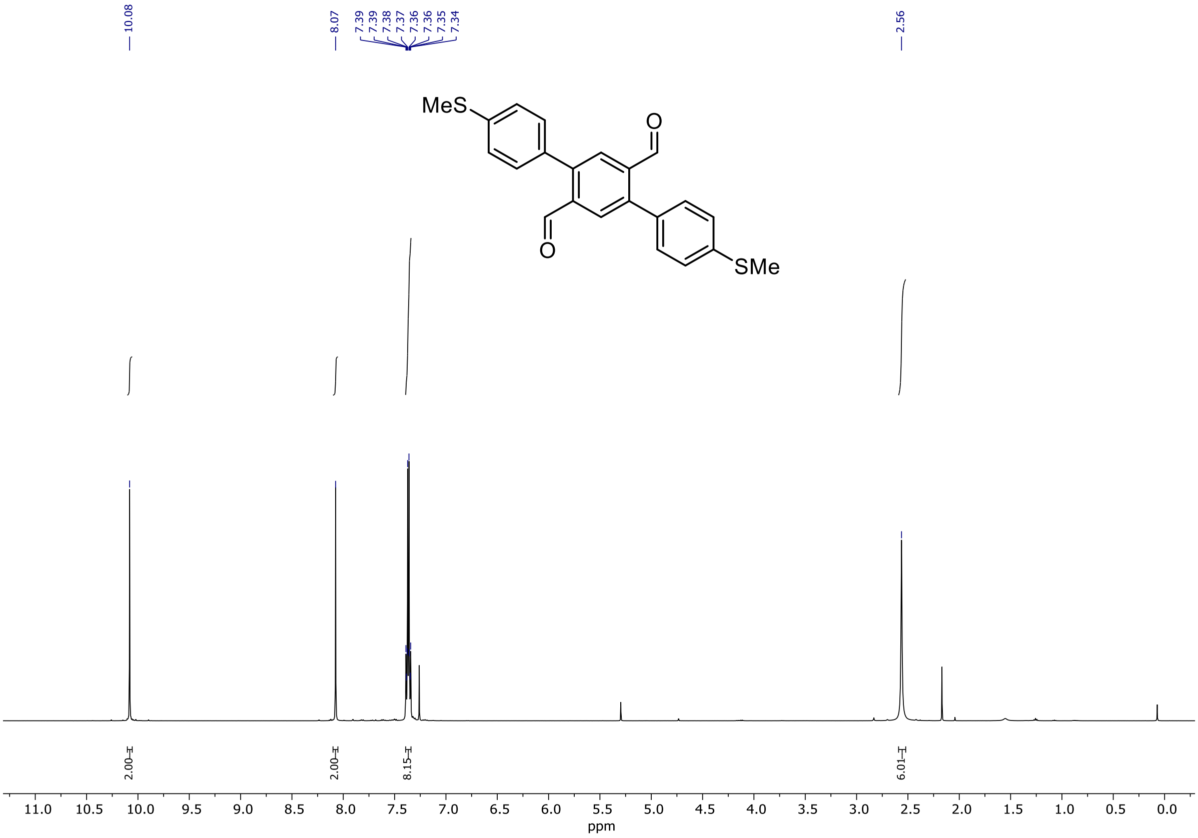

Figure S3. ¹H-NMR (500 MHz, $CDCl_3$) of compound **3**a

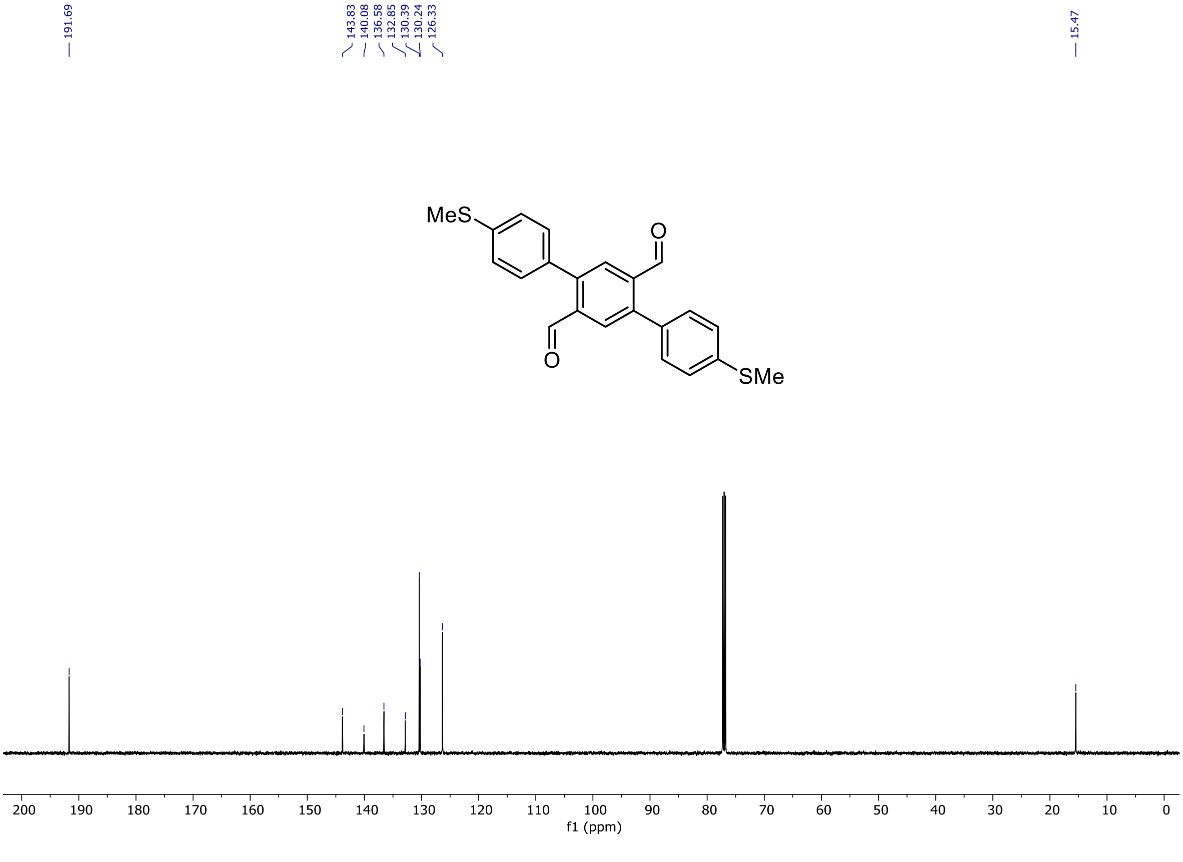

Figure S4. ¹H-NMR (500 MHz, $CDCl_3$) of compound 3**a**

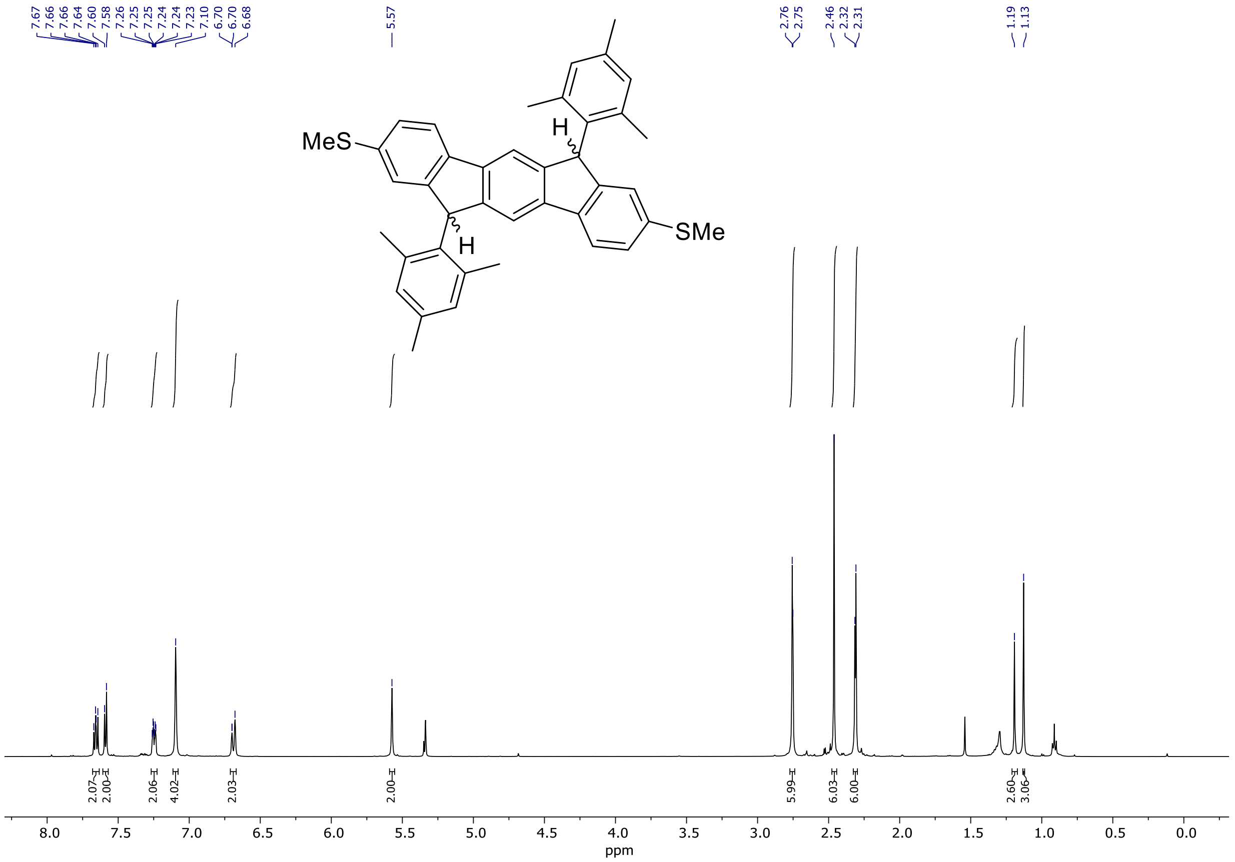

Figure S5. ^{1}H-NMR (500 MHz, CD_2Cl_2) of compound **DH-IFA**

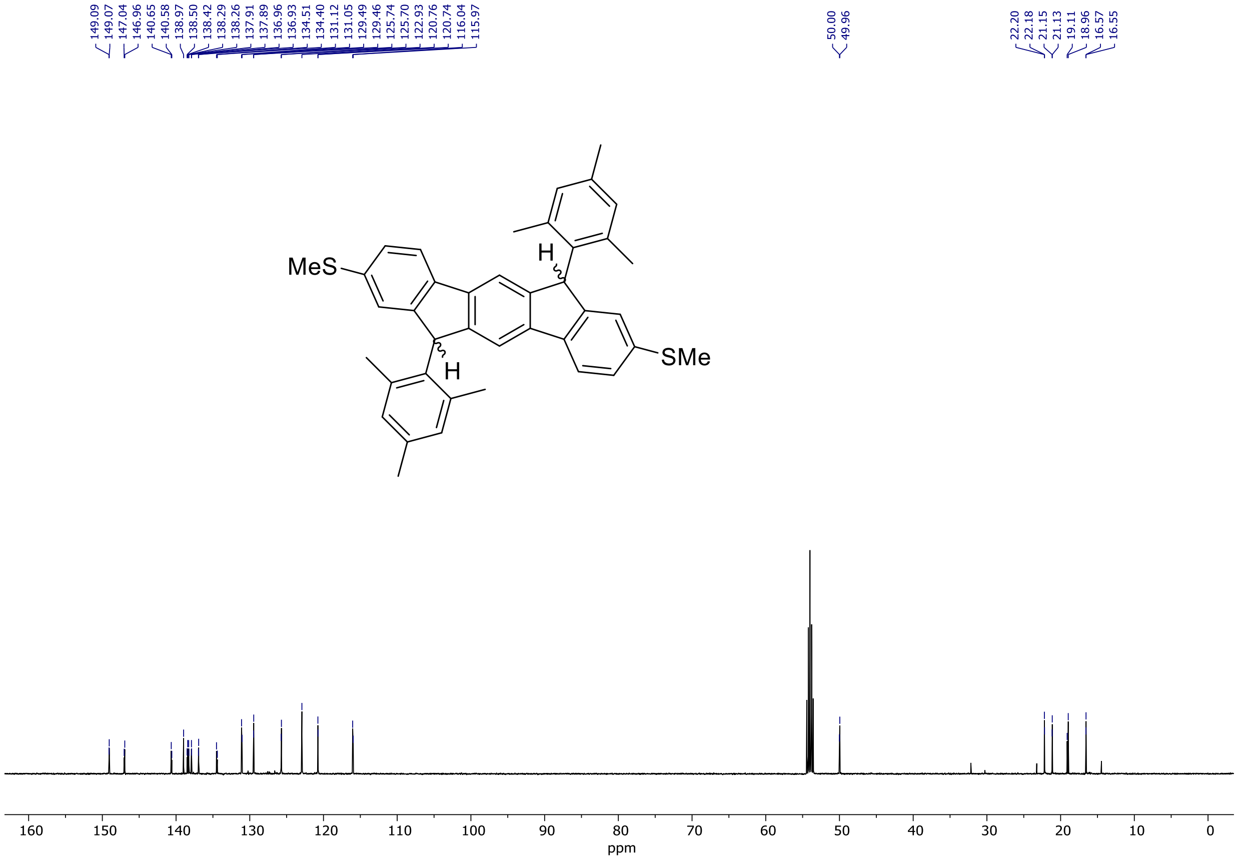

Figure S6. ^{13}C-NMR (126 MHz, CD_2Cl_2) of compound **DH-IFA**

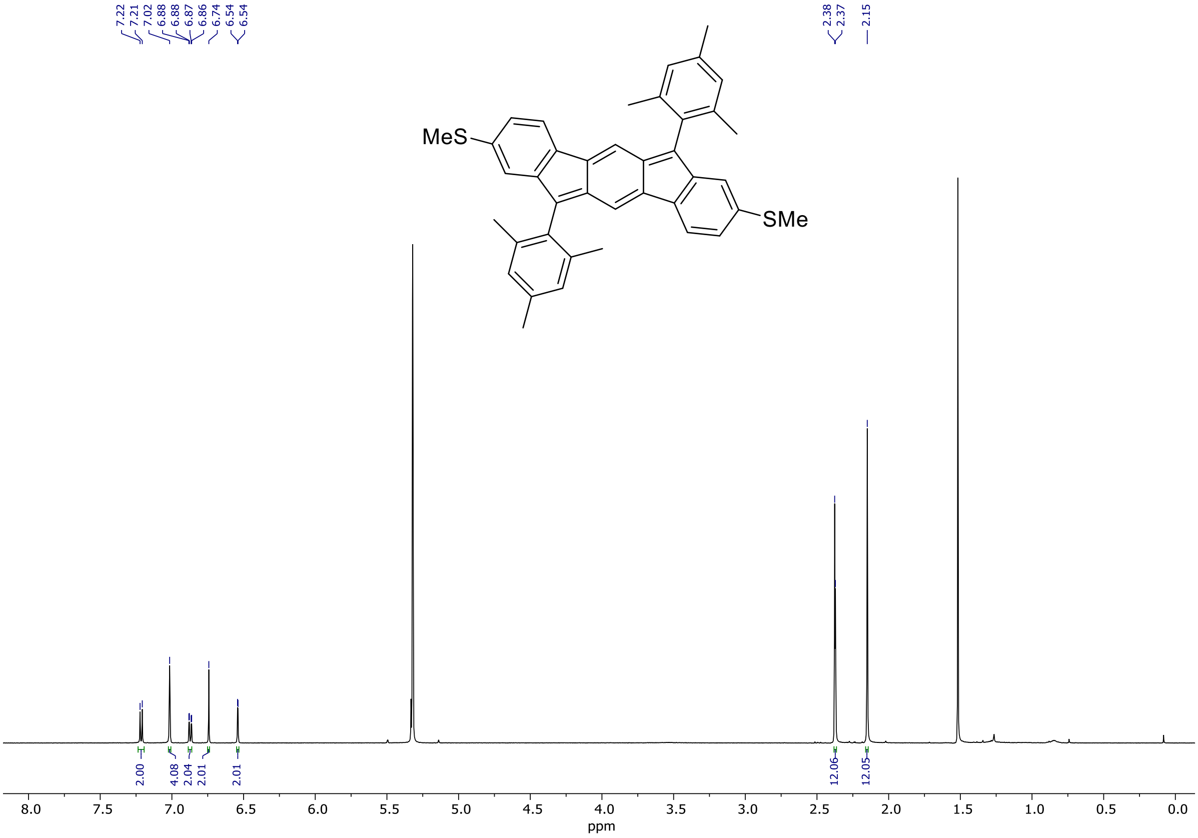

Figure S7. ^{1}H-NMR (500 MHz, CD_2Cl_2) of compound **IFA**

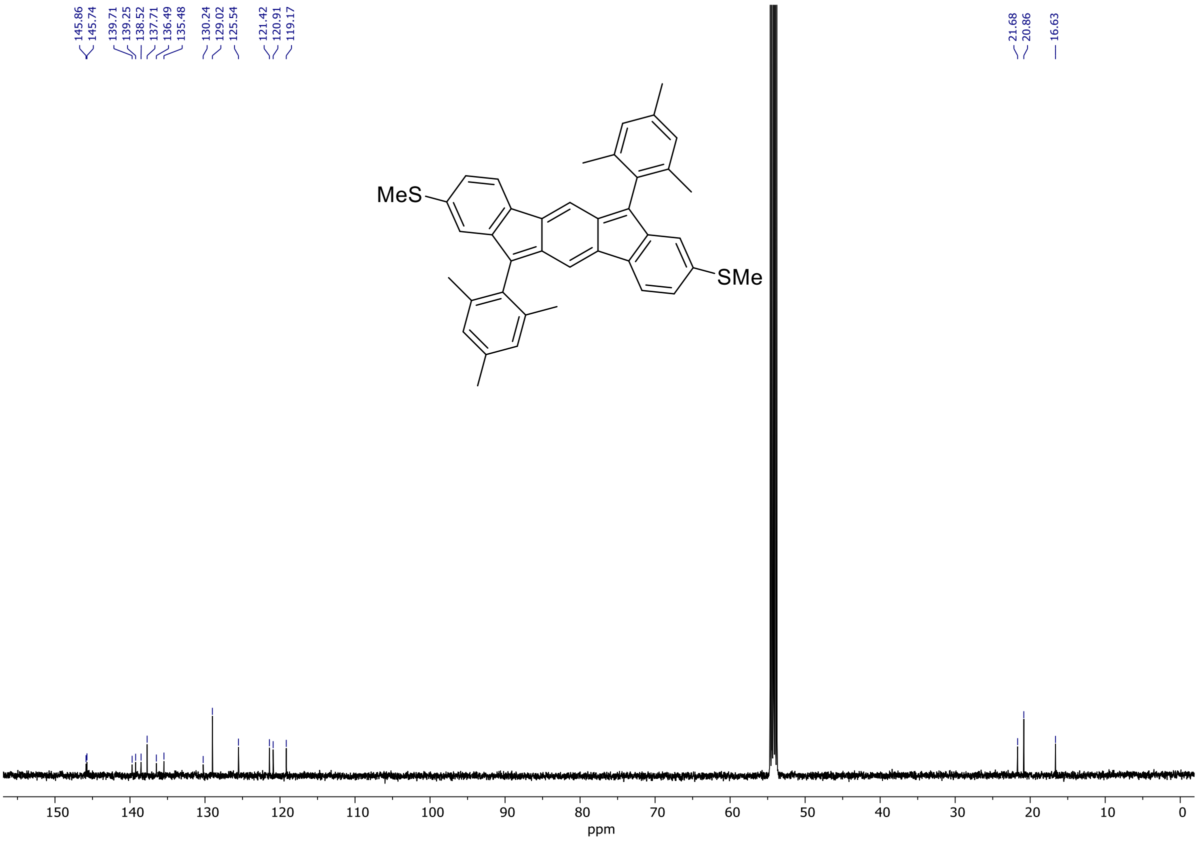

Figure S8. ^{13}C-NMR (126 MHz, CD_2Cl_2) of compound **IFA**

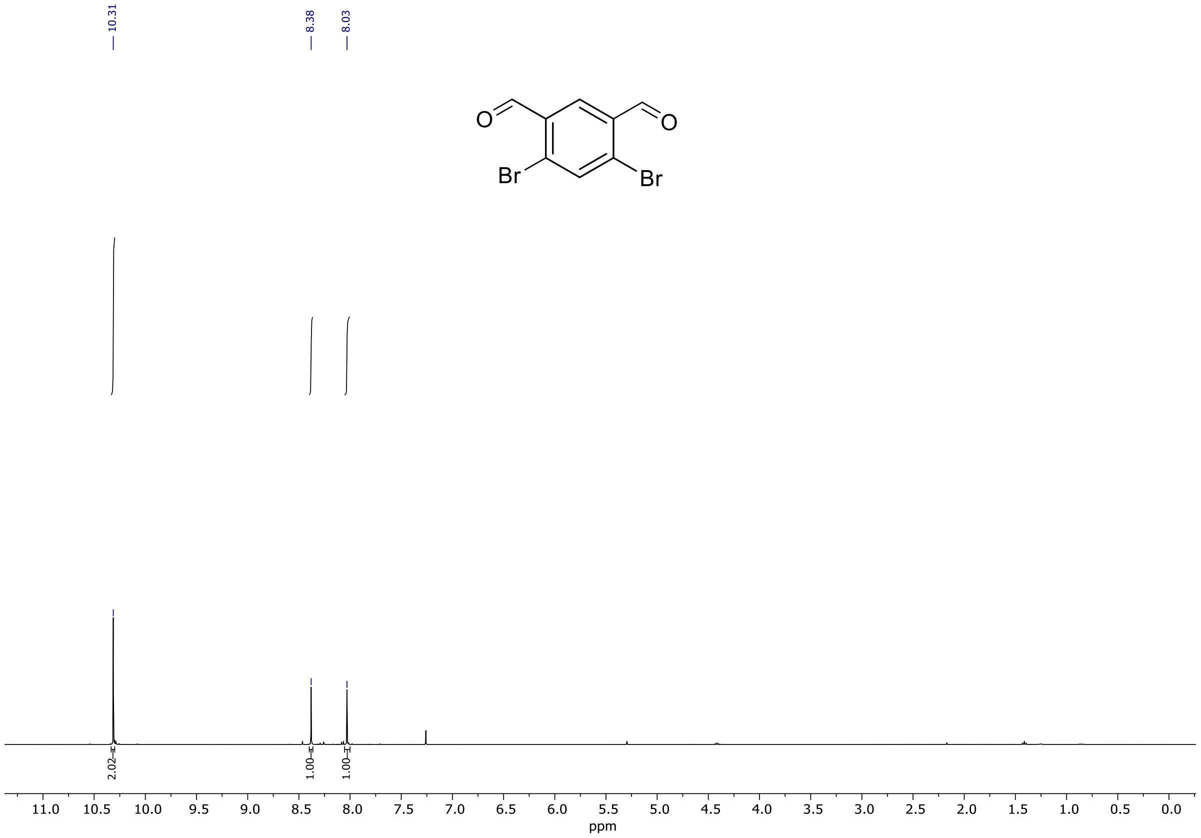

Figure S9. ^{1}H-NMR (500 MHz, $CDCl_3$) of compound **2b**

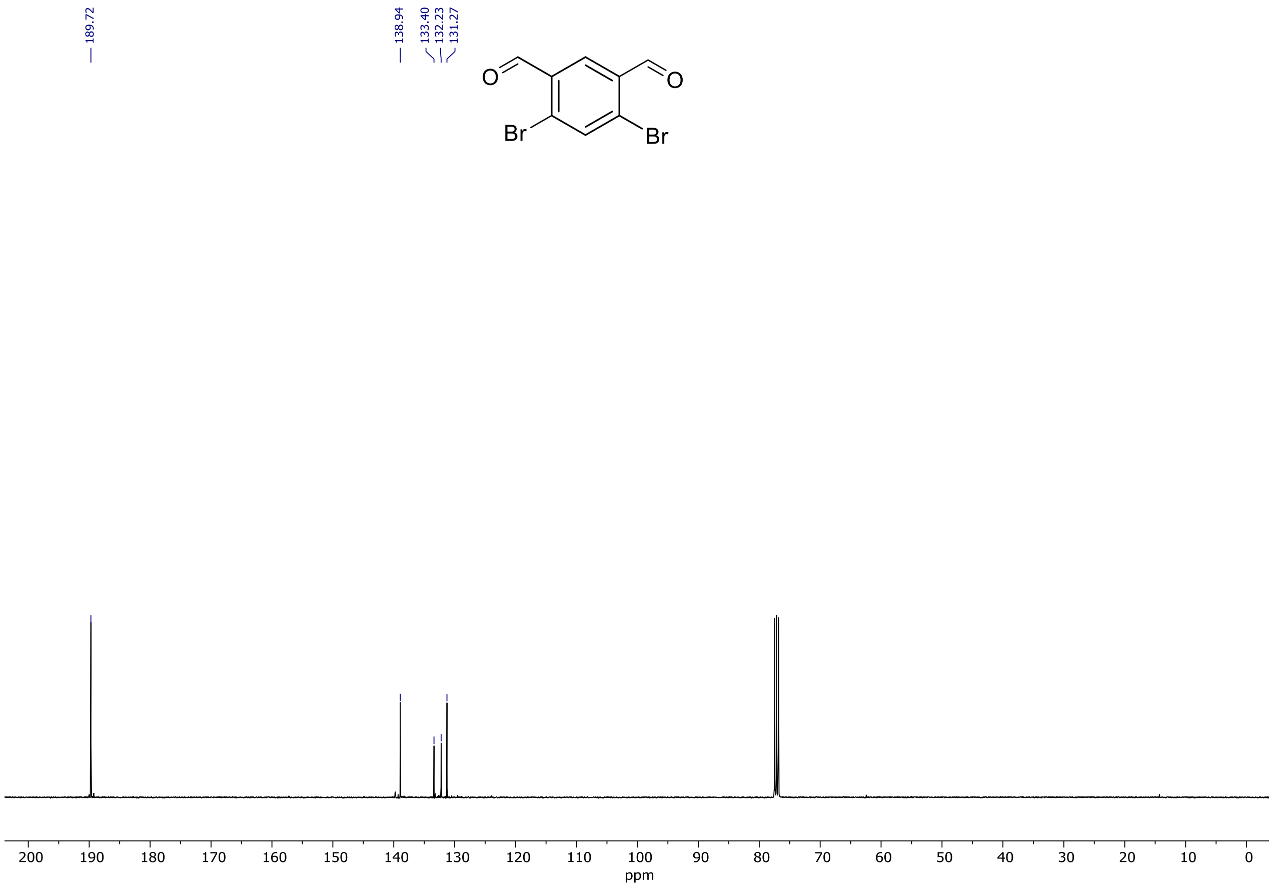

Figure S10. ^{13}C-NMR (126 MHz, CD_2Cl_2) of compound **2b**

^{1}H-NMR (500 MHz, $CDCl_3$) and ^{13}C-NMR (126 MHz, $CDCl_3$) of compound 3b

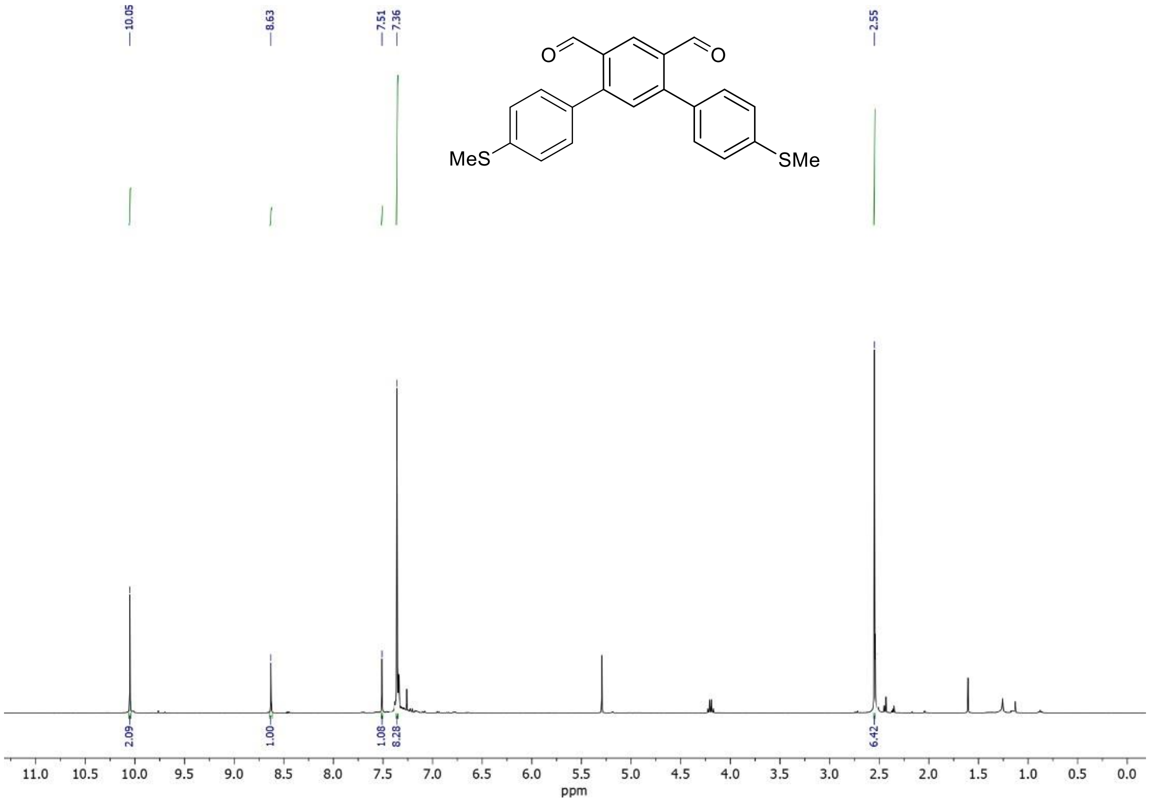

Figure S11. ^{1}H-NMR (500 MHz, $CDCl_3$) of compound **3b**

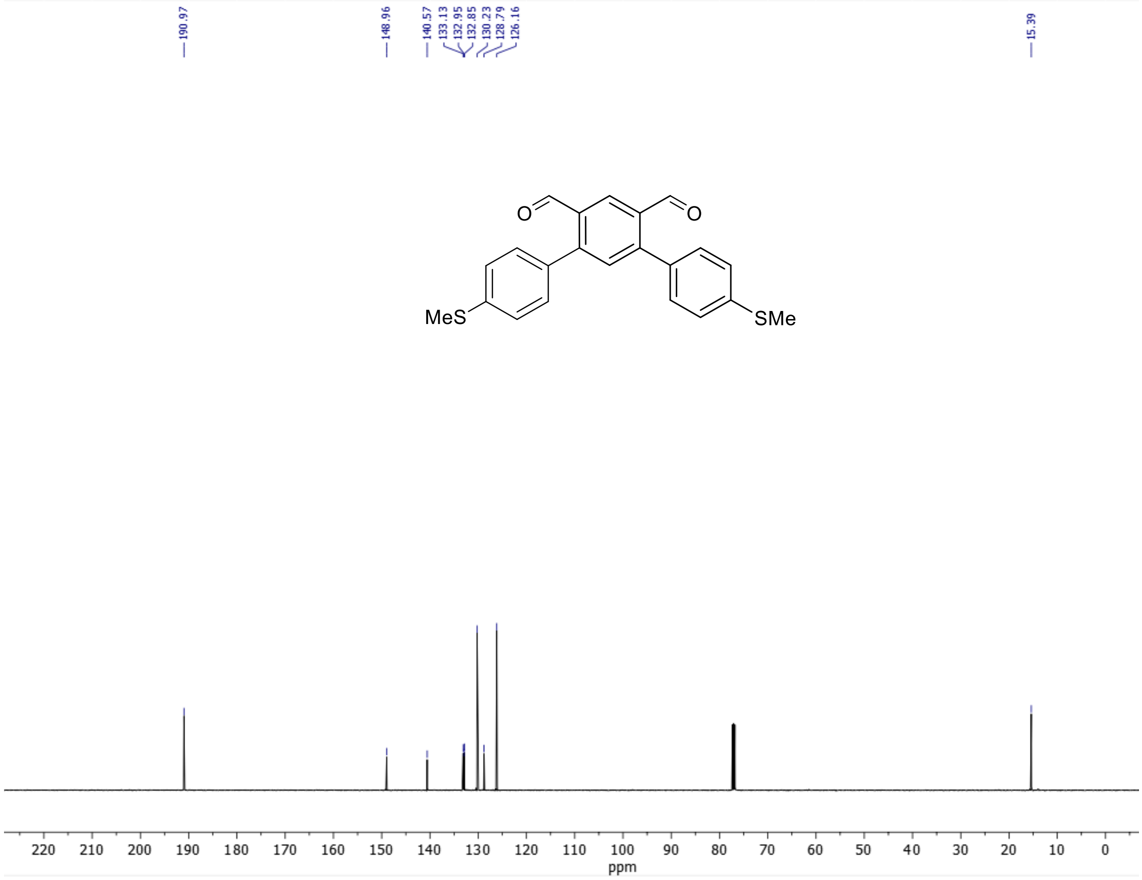

Figure S12. ^{13}C-NMR (126 MHz, CD_2Cl_2) of compound **3b**

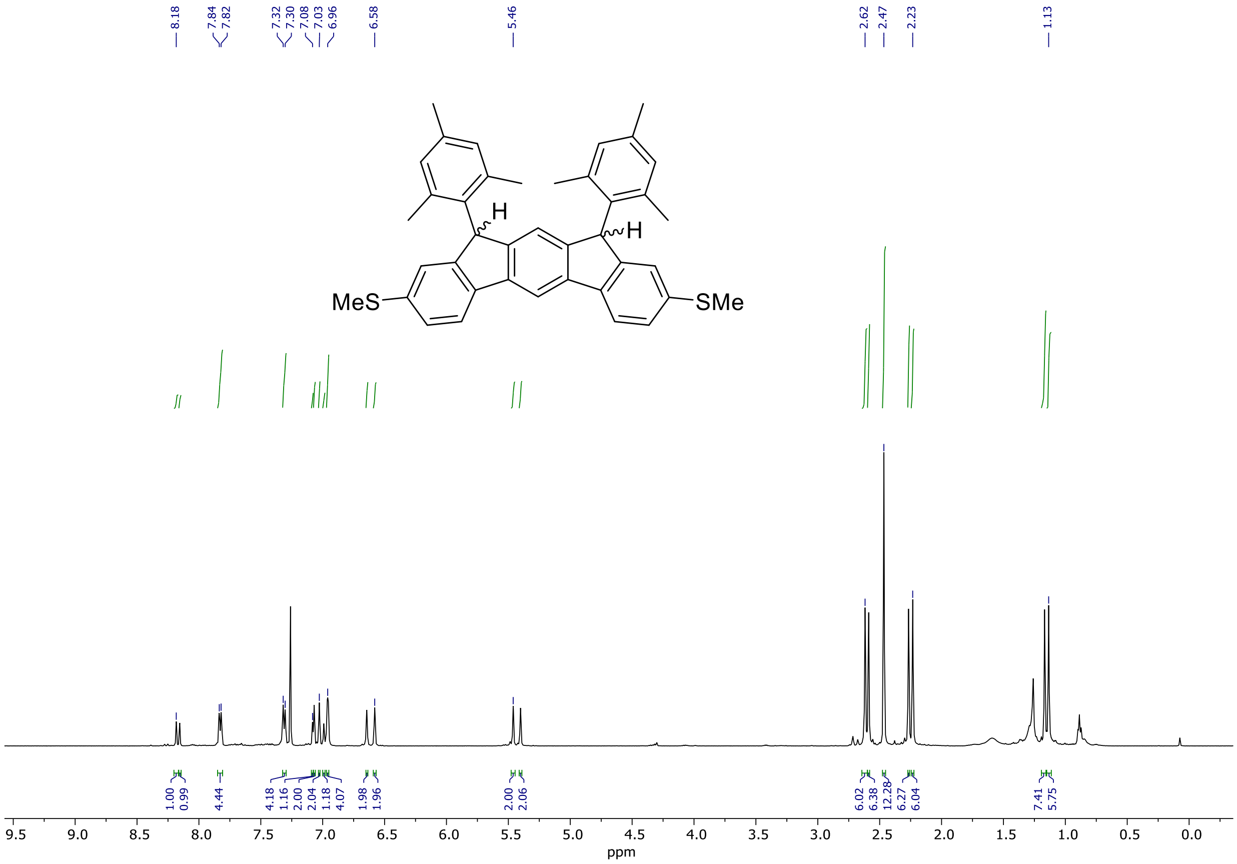

Figure S13. ^{1}H-NMR (500 MHz, $CDCl_3$) of compound **DH-IFS**

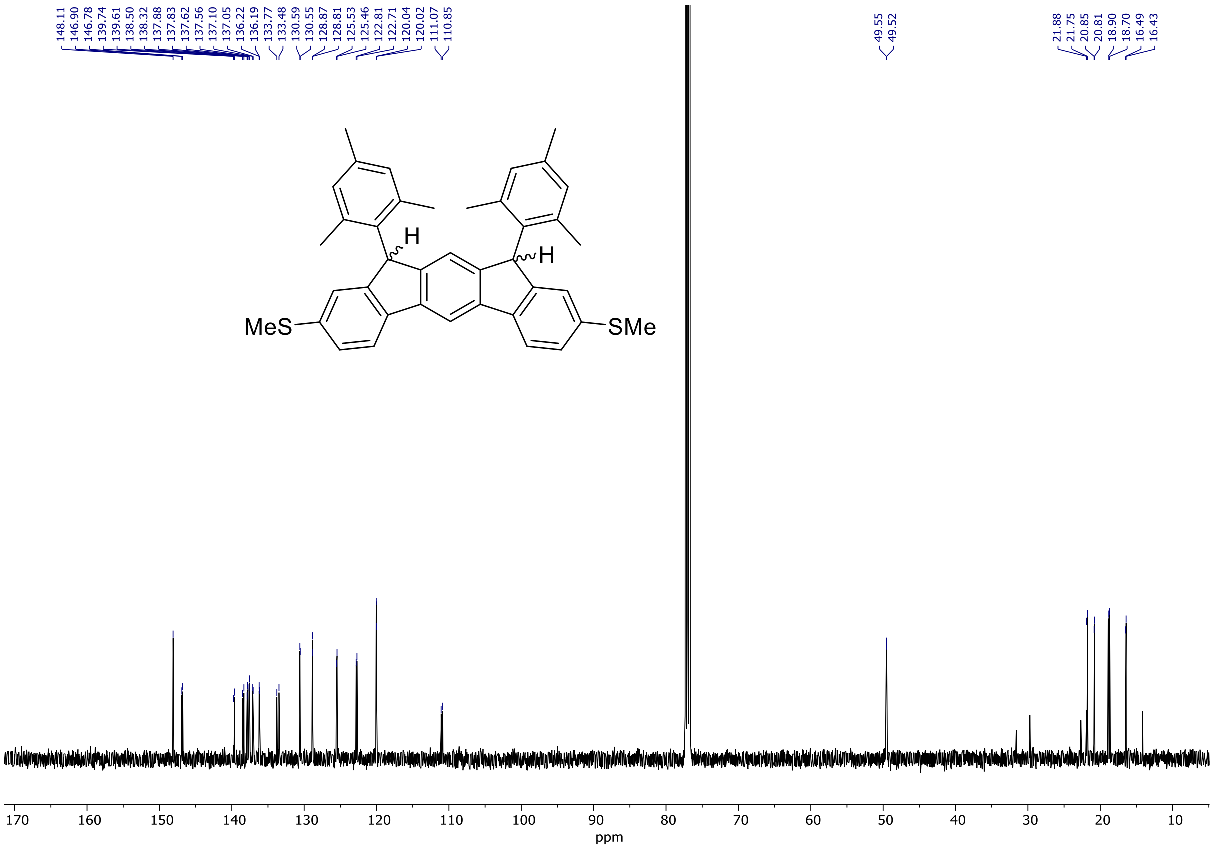

Figure S14. ^{13}C-NMR (126 MHz, CD_2Cl_2) of compound **DH-IFS**

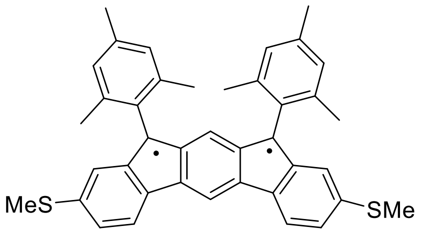

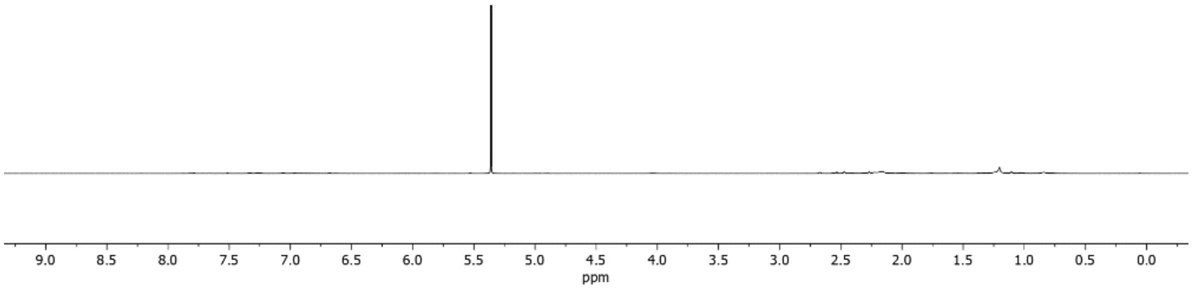

Figure S15. ^{1}H-NMR (500 MHz, CD_2Cl_2) of compound **IFS**

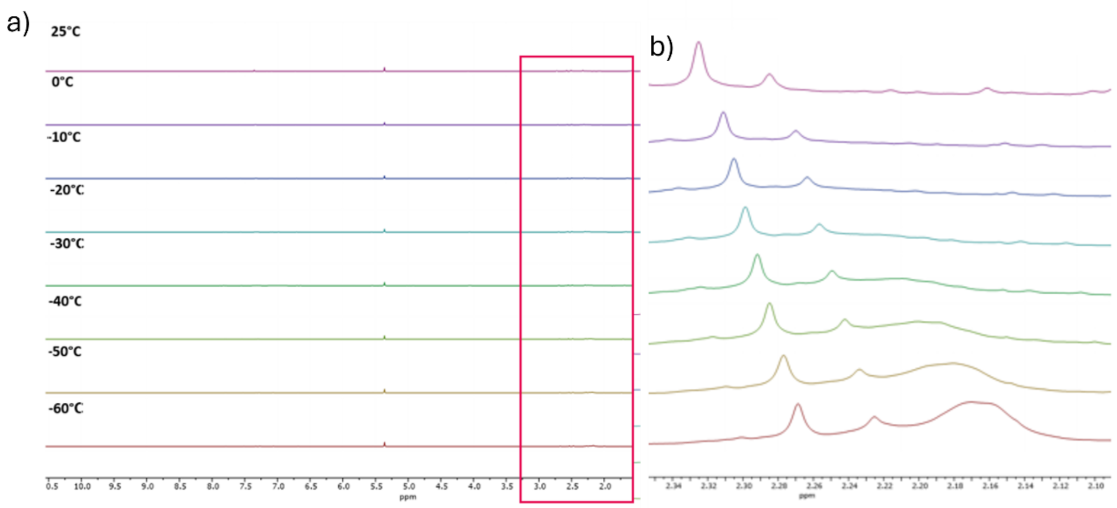

Figure S16. a) VT-^{1}H-NMR (500 MHz, CD_2Cl_2) of compound **IFS** from 25° C to –60 °C; b) Zoom in the 2.34-2.10 ppm region. A broad signal at 2.2 ppm corresponding to the methyl of the mesityl groups started to appear at –30 °C

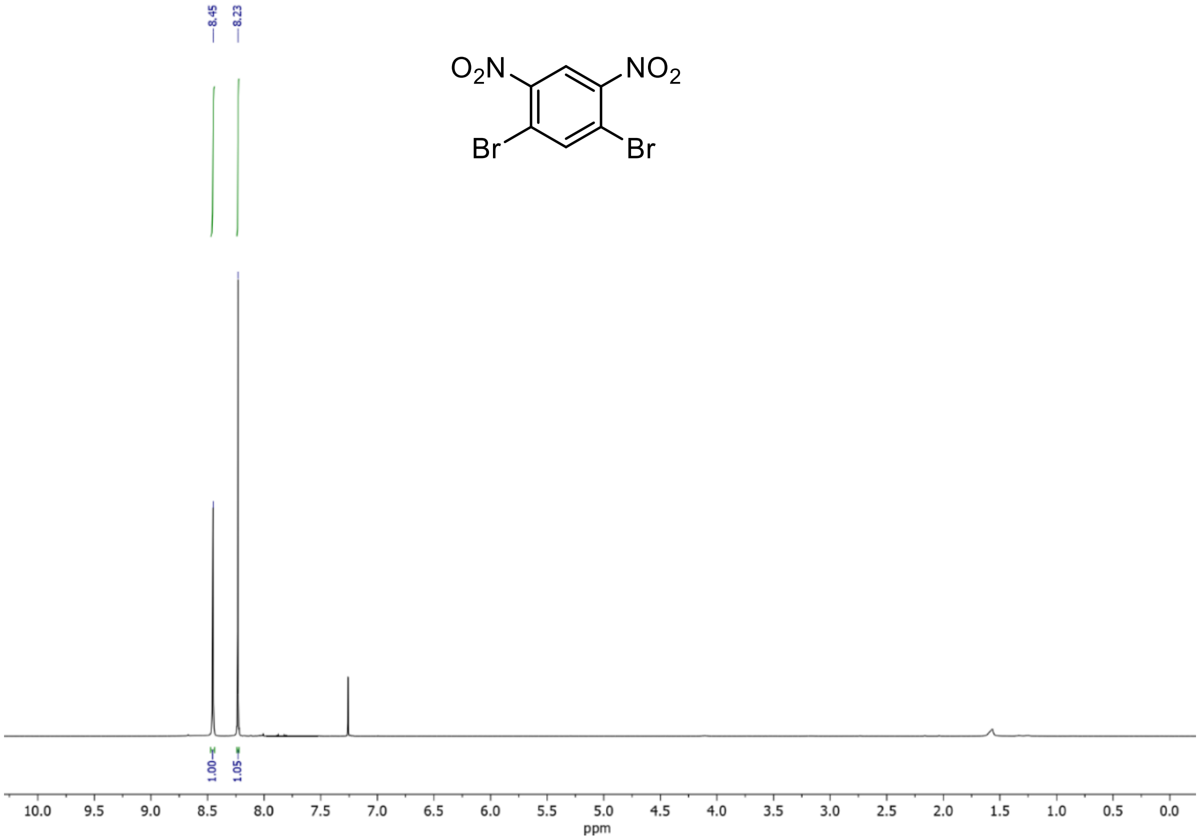

Figure S17. ^{1}H-NMR (500 MHz, $CDCl_3$) of compound **6**

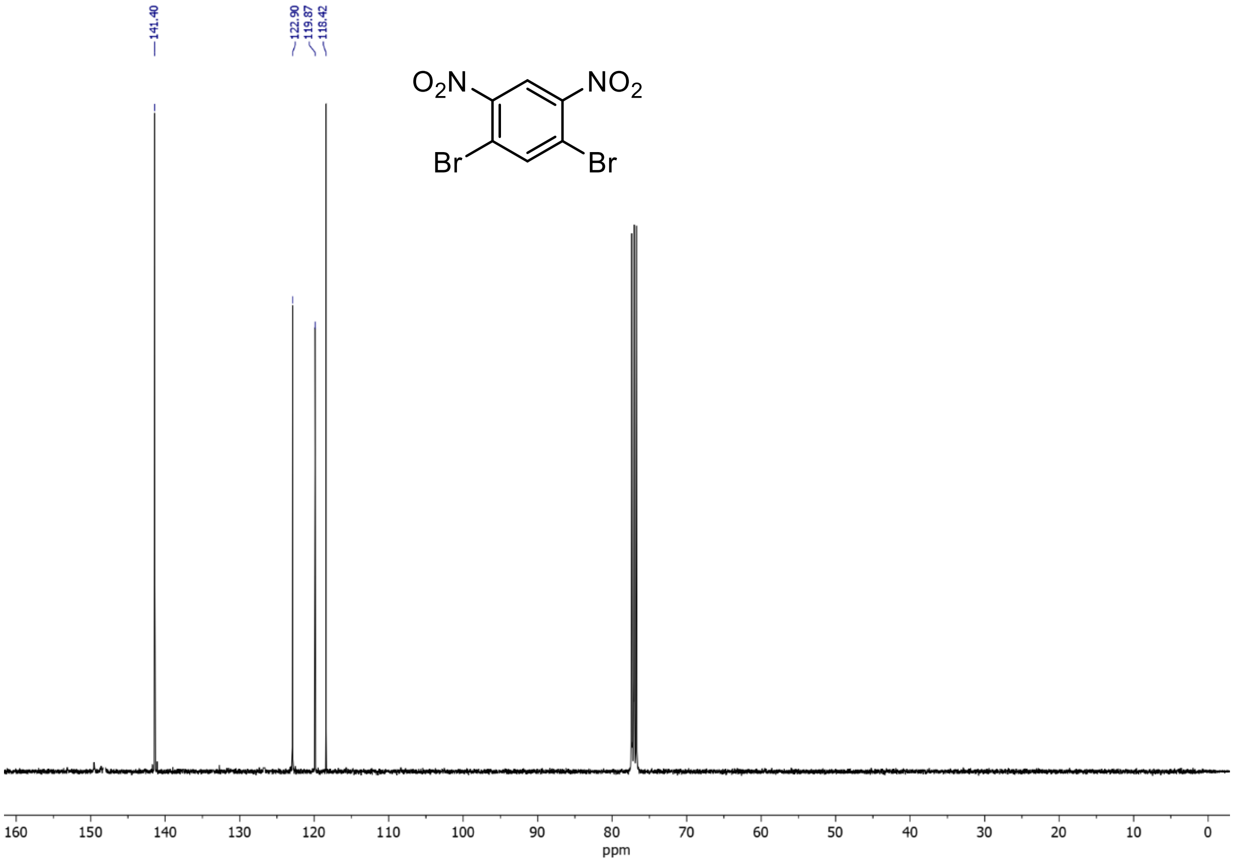

Figure S18. ^{13}C-NMR (126 MHz, $CDCl_3$) of compound **6**

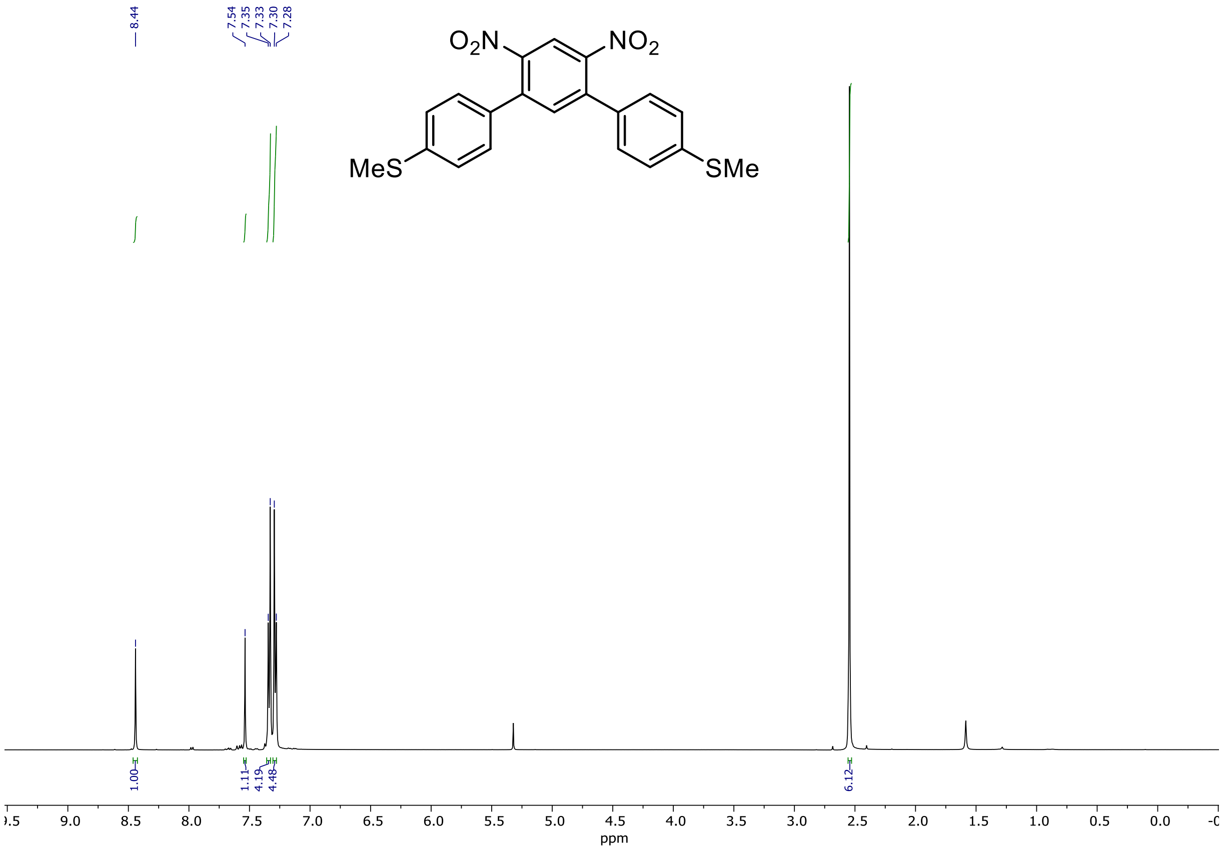

Figure S19. ^{1}H-NMR (500 MHz, $CDCl_3$) of compound **7**

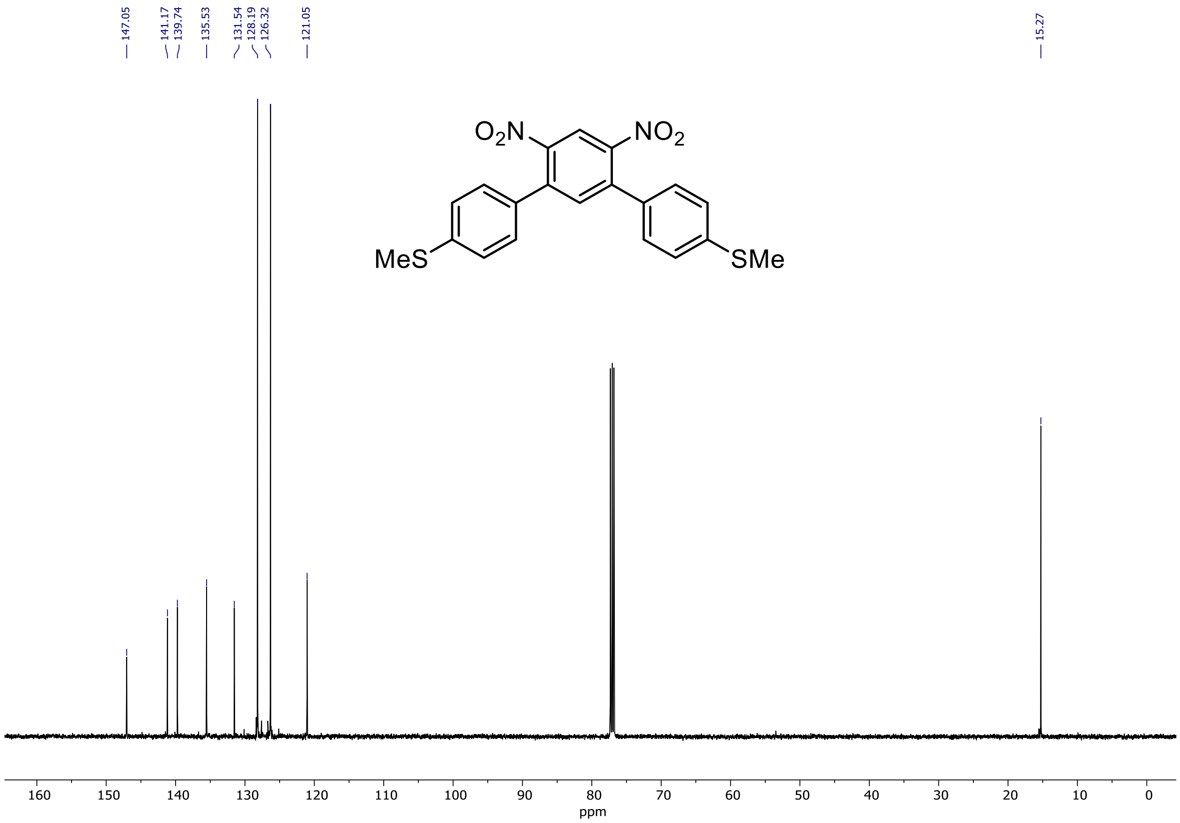

Figure S20. ^{13}C-NMR (126 MHz, $CDCl_3$) of compound **7**

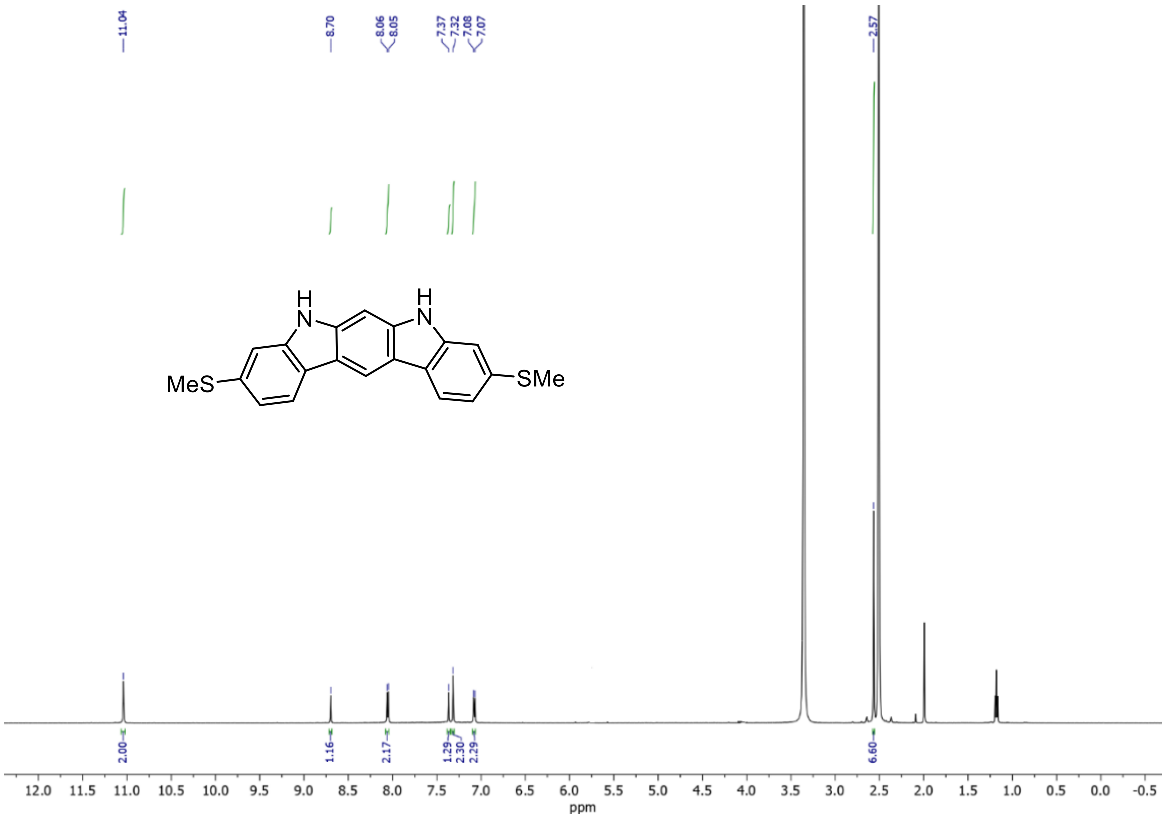

Figure S21. ^{1}H-NMR (500 MHz, DMSO-d$_6$) of compound **8**

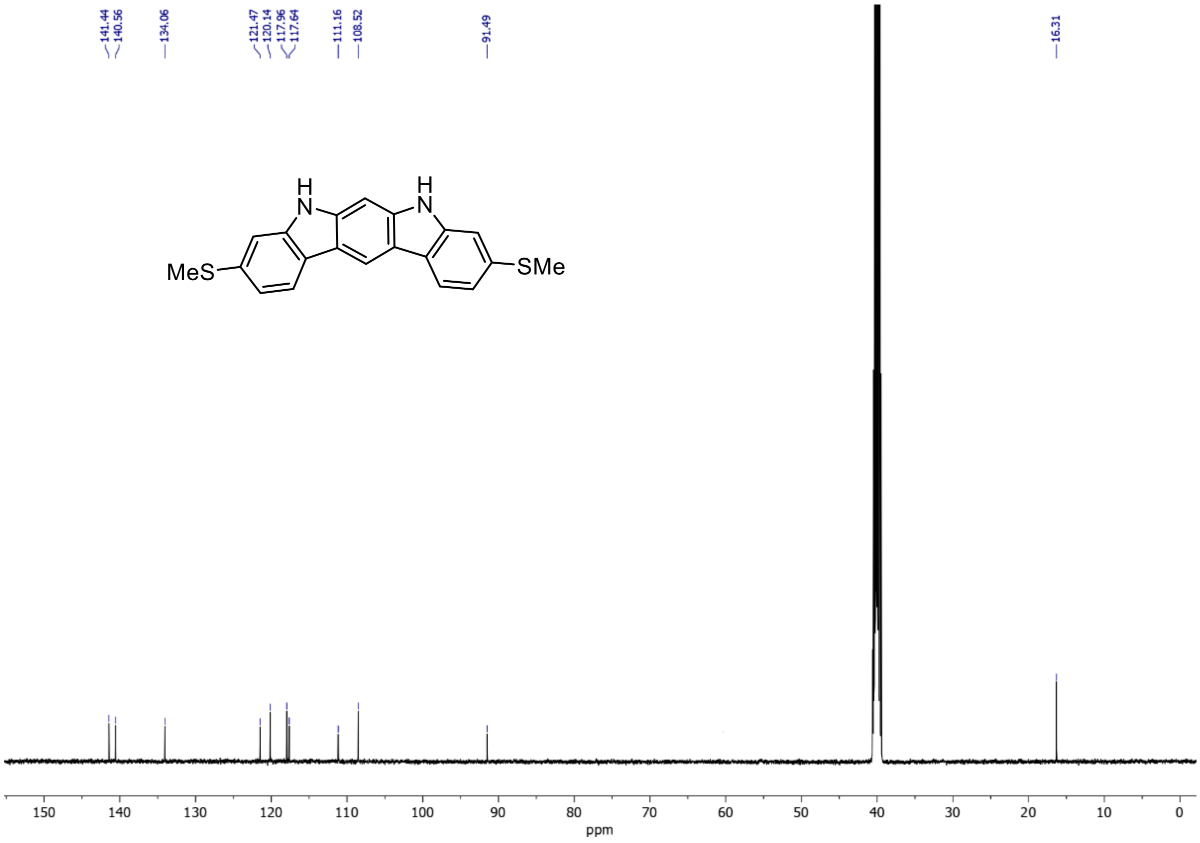

Figure S22. ^{13}C-NMR (126 MHz, DMSO-d$_6$) of compound **7**

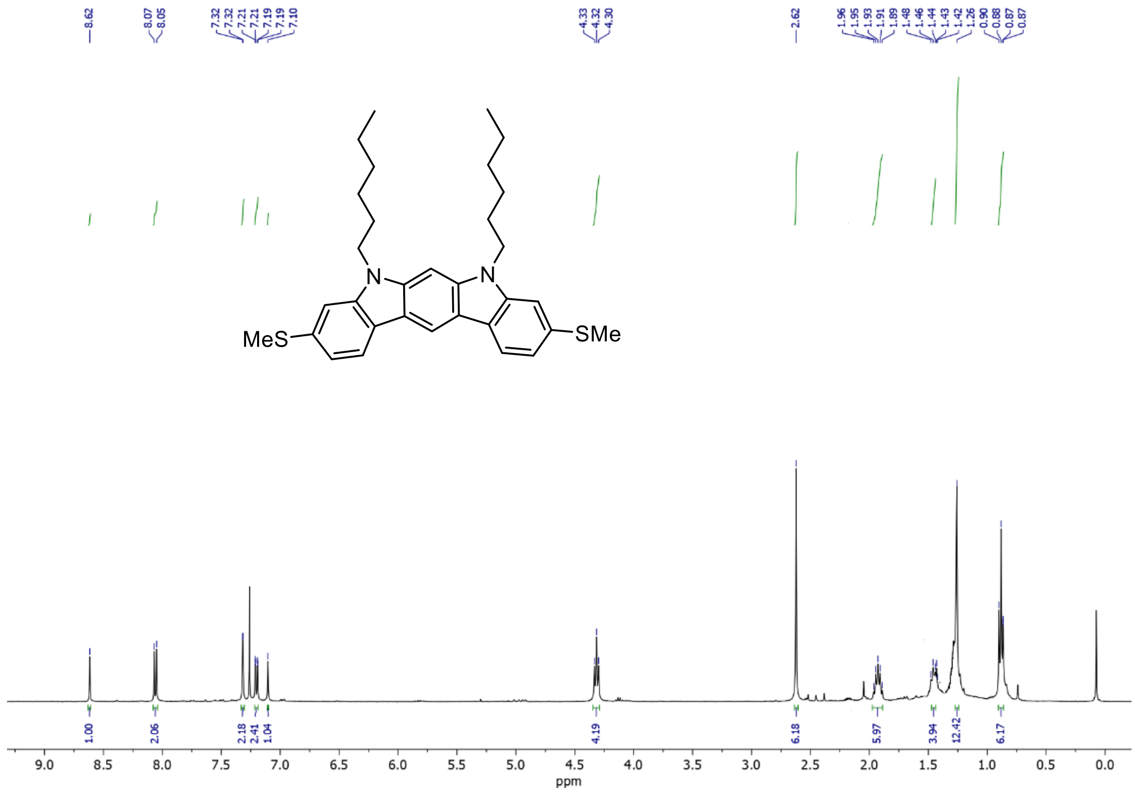

Figure S23. ^{1}H-NMR (500 MHz, $CDCl_3$) of compound **IFSN**

4. High-resolution mass spectra. Isotopic distribution

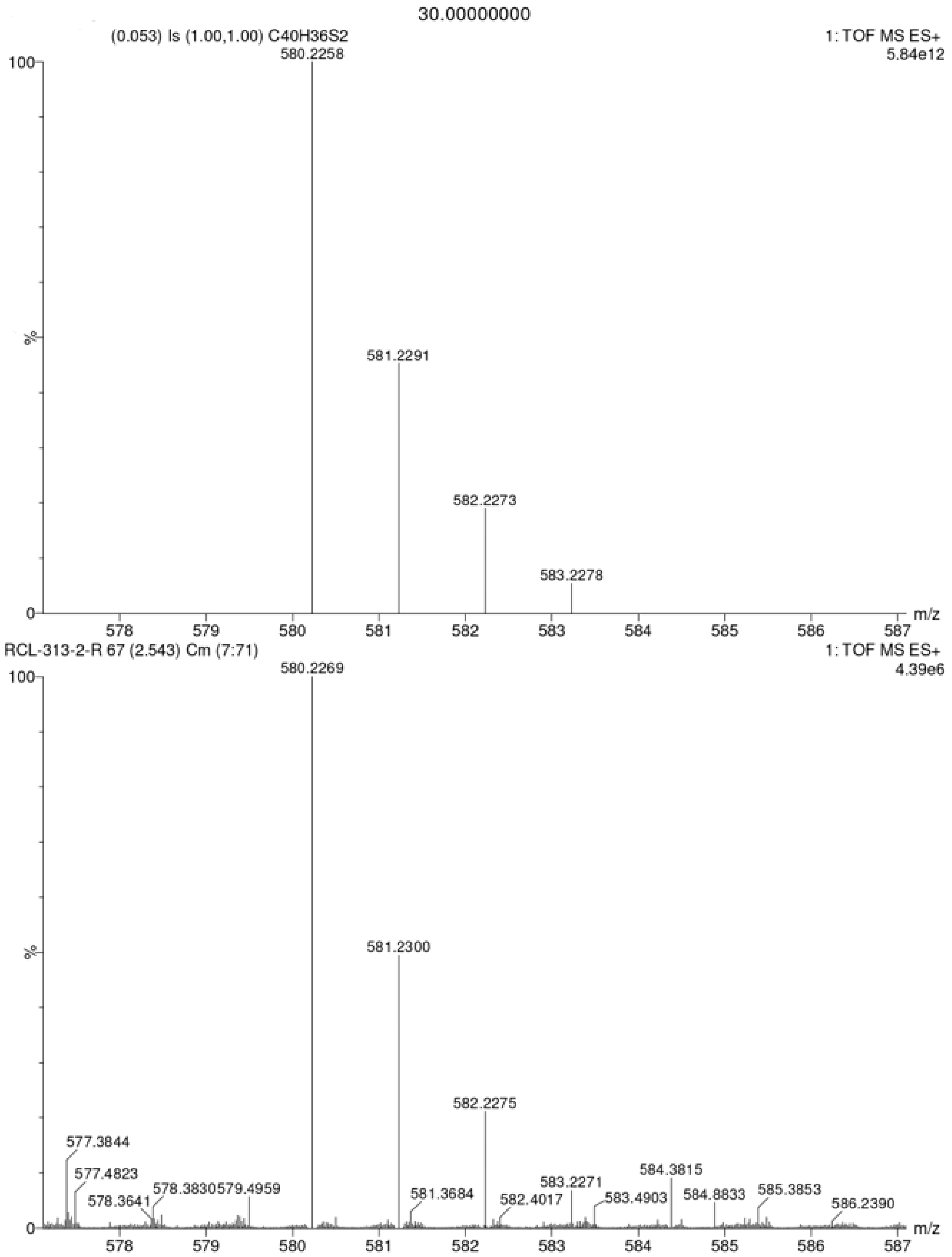

Figure S24. HRMS (ESI) isotopic distribution of the $[M]^+$ peak of compound **IFA**. Top: Calculated. Bottom: Experimental.

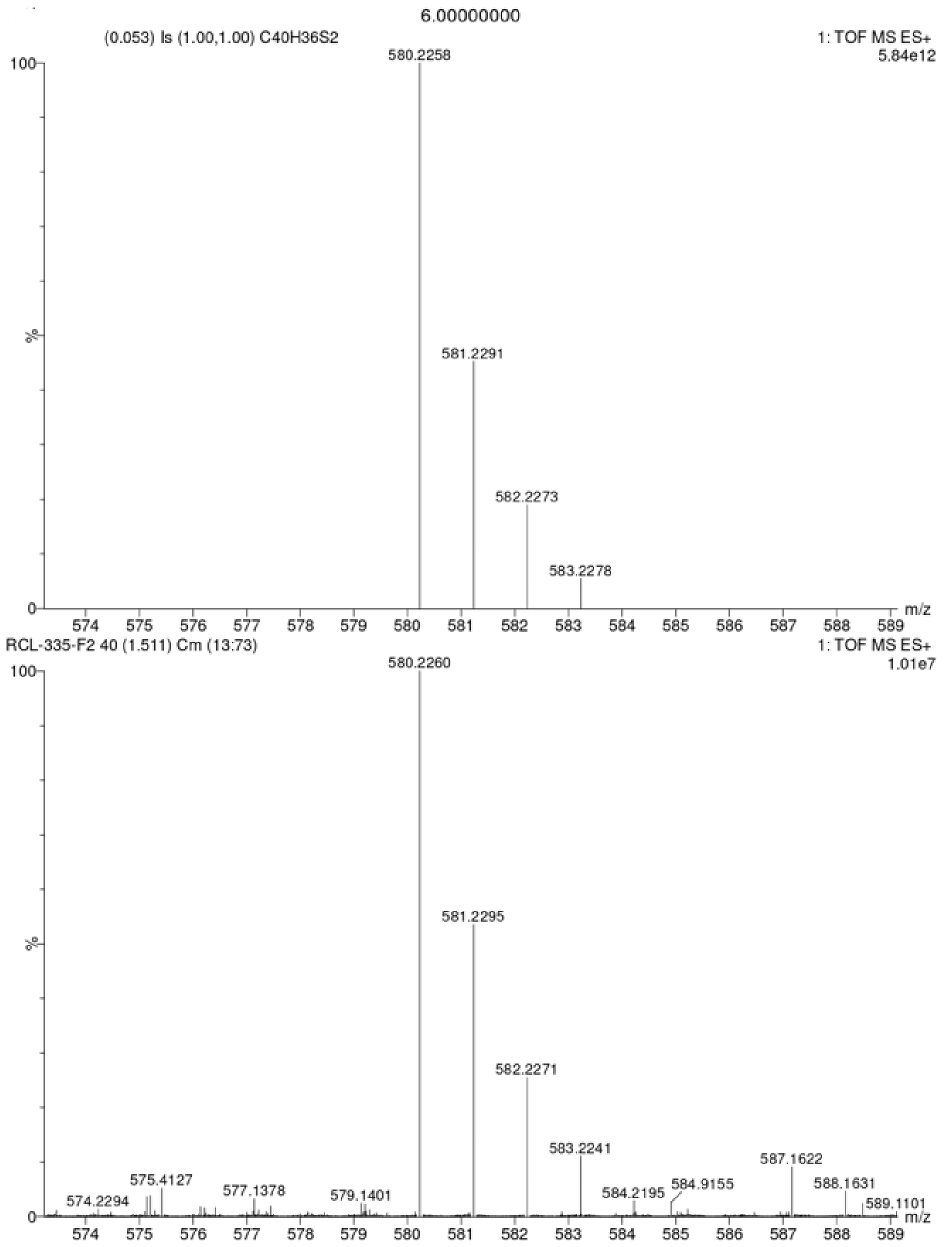

Figure S25. HRMS (ESI) isotopic distribution of the $[M]^+$ peak of compound **IFS**. Top: Calculated. Bottom: Experimental.

5. Single-crystal X-Ray diffraction

X-ray diffraction quality single crystals of compound **IFA** were obtained by slow evaporation of a solution of the compound in a mixture of hexane/DCM (1:1). The diffraction data was collected with a Bruker D8 Venture diffractometer equipped with a Mo radiation source and a PHOTON III detector. The SHELXT,[S3] program was used to solve the structure, which was refined with SHELX 2019,[S4] within the WinGX32 graphical interface,[S5] using the full-matrix least-squares against F^2 procedure. C–H hydrogen atoms were placed in idealized positions (U_{eg}(H) = 1.2U_{eg}(C) or U_{eg}(H) = 1.5U_{eg}(C)) and were allowed to ride on their parent atoms.

X-ray diffraction measurement and refinement data for **IFA**: Chemical formula, $C_{40}H_{36}S_2$; *Mr*, 580.81; crystal size [mm^3], 0.292 x 0.123 x 0.069; temperature, 100(2) K; wavelength [Å], 0.71073 (Mo Kα), crystal system, monoclinic; space group, *C*2/*c*; *a* [Å], 23.794(3); *b* [Å], 7.4126(8); *c* [Å], 18.550(2); α [°], 90; β [°], 108.723(4); γ [°], 90; *V* [Å^3], 3098.7(6); *Z*, 4; ρ_{calcd} [mg m^{-3}], 1.245; μ [mm^{-1}], 0.200; F(000), 1232; ϑ range [°], 2.319 to 28.970; *hkl* ranges, −32/31, −9/10, −25/24; reflections collected, 33123; independent reflections, 4077; R_{int}, 0.0777; completeness to ϑ = 25.242°, 100.0%; absorption correction, semi-empirical from equivalents; refinement method; full-matrix least-squares on F^2 ; Final *R* indices [I>2σ(I)], R_1 = 0.0577, wR_2 = 0.1147; *R* indices (all data), R_1 = 0.0796, wR_2 = 0.1225; goodness-of-fit on F^2 , 1.109.

Deposition Number 2333147 contains the supplementary crystallographic data for this paper. These data are provided free of charge by the joint Cambridge Crystallographic Data Centre and Fachinformationszentrum Karlsruhe Access Structures service.

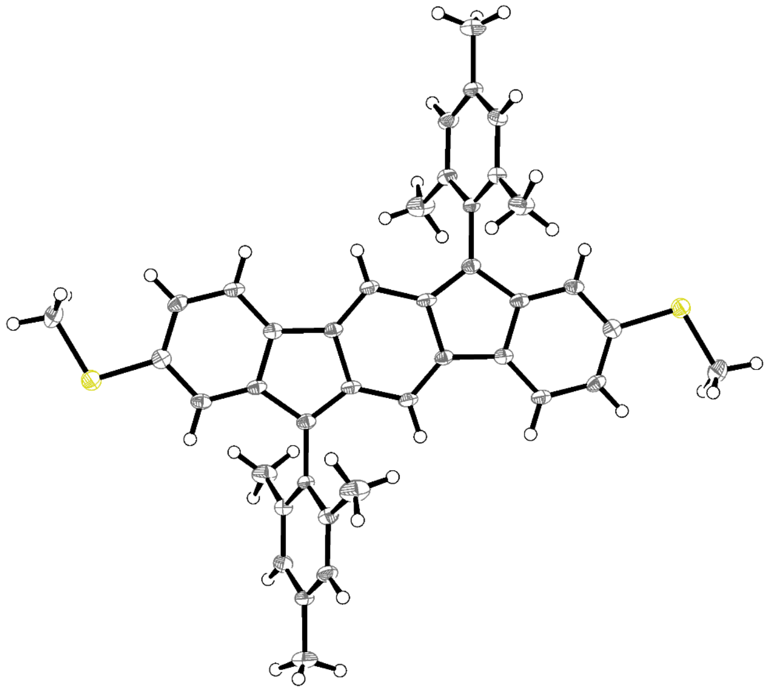

Figure S26. ORTEP plot of the X-ray diffraction structure of compound **IFA**. Thermal ellipsoids are displayed at 50% probability. Colour coding: C, grey, S, yellow, H, white.

6. EPR spectrum for IFS

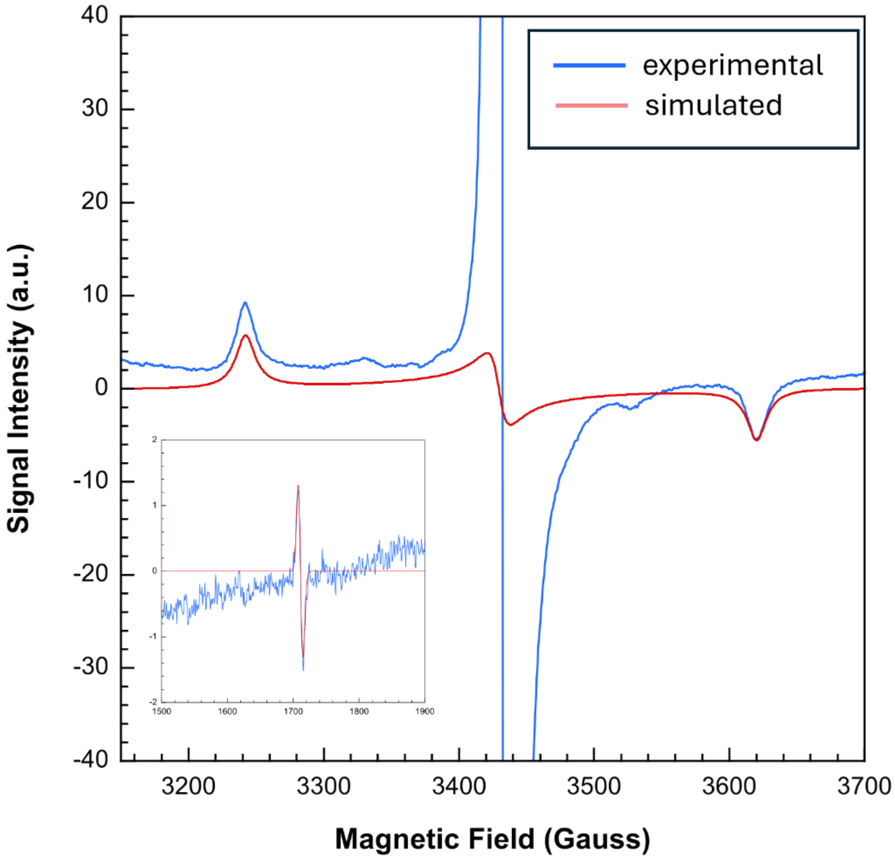

Figure S27. ESR spectra of **IFS**. a) Simulated (red) and observed (blue) spectra ($\Delta m_s = \pm1$) at 298 K. The central signal of the observed spectrum is due to a monoradical impurity. Inset: $\Delta m_s = \pm2$ forbidden signal characteristic of the triplet state (the baseline is not corrected). The microwave frequency used was 6 GHz. The experimentally determined zero-field splitting parameters are D = 189 Gauss and E = 63 Gauss (g = 2.007).

7. UV-Vis spectra

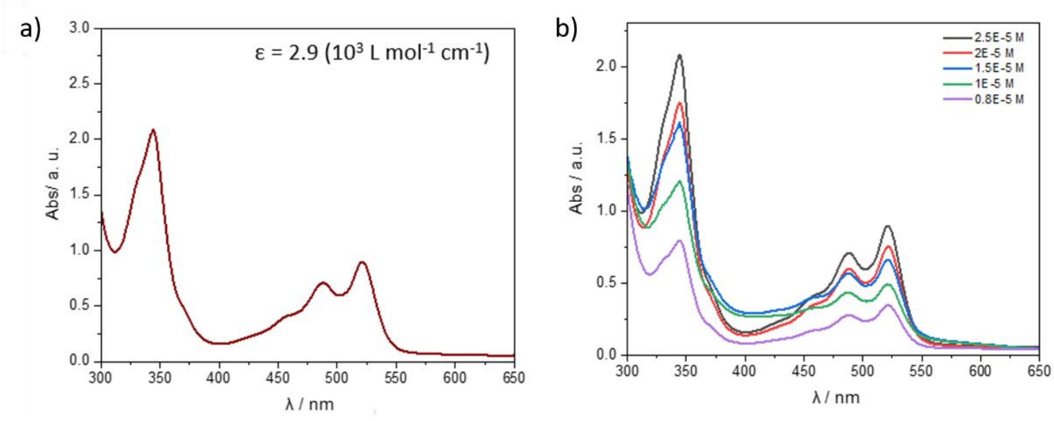

Figure S28. a) UV-Vis spectra of **IFA** in DCM (2.5×10^{-5} M); b) UV-Vis spectra of **IFA** in DCM at different concentrations. ε at 521 nm;

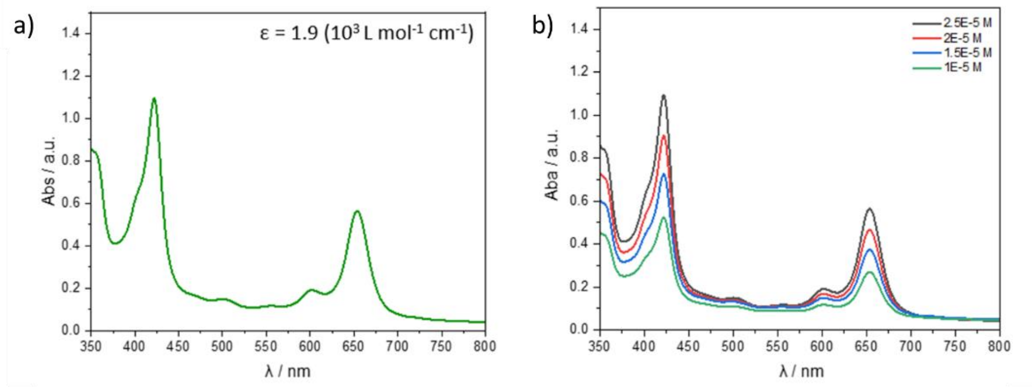

Figure S29. a) UV-Vis spectra (350-800 nm region) of **IFS** in DCM under inert conditions (1.5×10^{-5} M); b) UV-Vis spectra of **IFS** in DCM under inert conditions at different concentrations. ε at 653 nm.

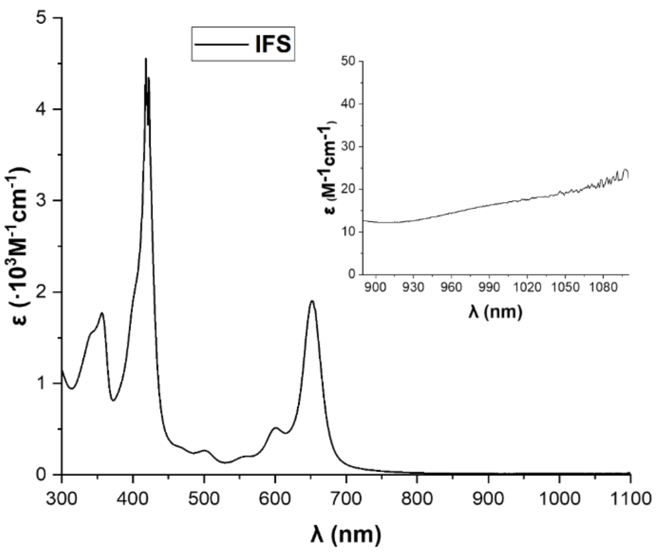

Figure S30. UV-vis spectrum of **IFS** in DCM under inert conditions. Inset shows a magnified view of the 900-1095 nm region.

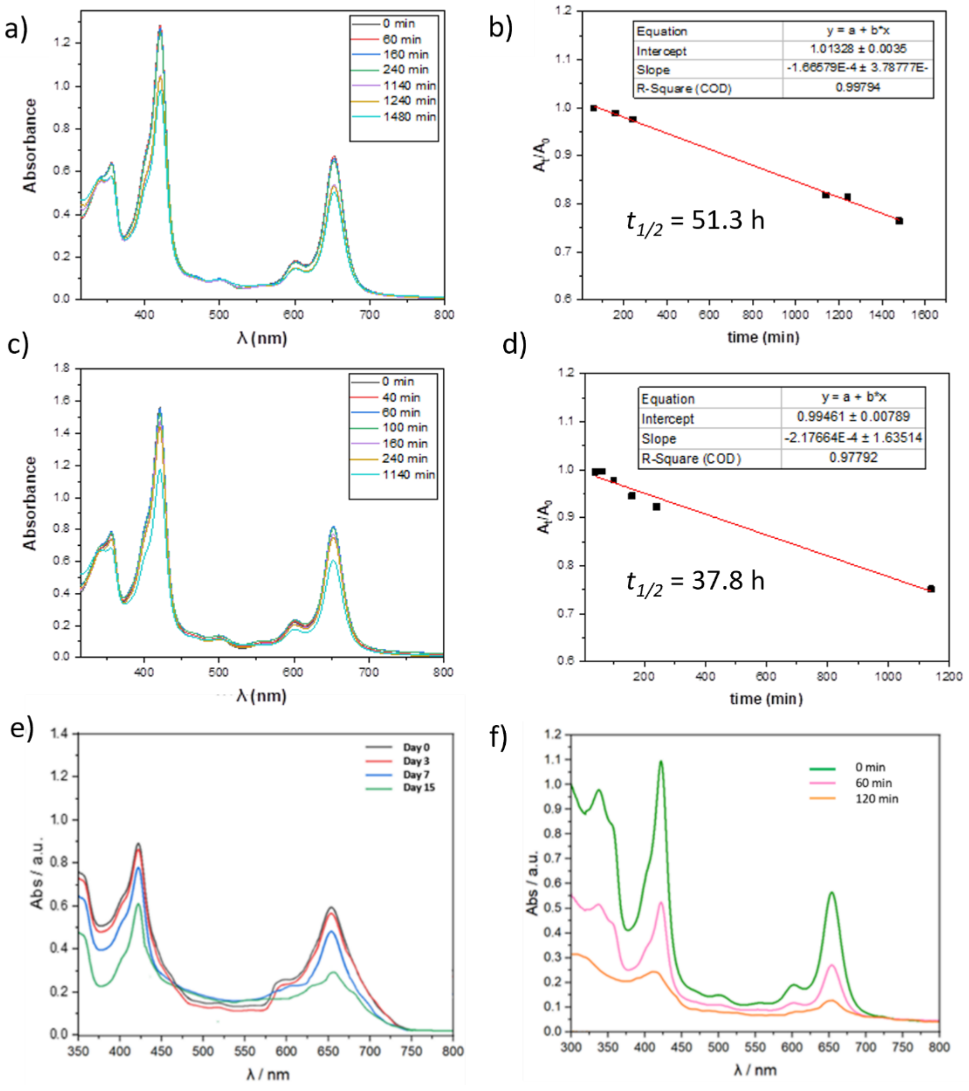

Figure S31. a) Stability of a solution of **IFS** in anhydrous DCM under inert atmosphere at r.t. monitored by UV-vis absorbance.; b) Time-dependant absorption decay (λ=653 nm) of a solution of **IFS** in anhydrous DCM under inert atmosphere at r.t. Zero-order kinetics, $t_{1/2}$= 51.3 h; c) Stability of a solution of **IFS** in DCM under ambient atmosphere at r.t. monitored by UV-vis absorbance.; d) Time-dependant absorption decay (λ=653 nm) of a solution of **IFS** in DCM under ambient atmosphere at r.t. Zero-order kinetics, $t_{1/2}$= 37.8 h; e) Evolution of the UV-Vis spectrum of a sample stored as a solid, under argon and at –20°C. The same concentration was used for every measurement; f) Evolution of the UV-Vis spectrum of a sample stored as a solid under ambient atmosphere and submitted to an evaporation/redissolution process between measurements. The same concentration was used for every measurement.

8. Voltammograms

Cyclic Voltammetry (CV) was carried out on a PGSTAT2014 potentiostat/galvanostat (Metrohm Autolab B. V.) with a three-electrode cell under Ar atmosphere at 25 °C. A Pt-wire counterelectrode, an Ag wire quasireference electrode and a glassy carbon disk working electrode were used. CH_2Cl_2 was used as solvent to prepare a 0.1 M solution of tetra-*n*-butylammonium hexafluorophosphate ($TBAPF_6$) which was used as work solution. The Pt wire counter electrode and Ag wire quasi-reference electrode were cleaned by flame treatment. The glassy carbon disc working electrode was polished according to the literature.[S6] A 1.5 mM solution of **IFA** or **IFS** in CH_2Cl_2 was used. The scan rate was 0.05 V/s. The starting potential was 0 V against Ag reference electrode. Potential values are referred to ferrocenium/ferrocene (Fc^+/Fc). Fc added as an internal reference after each measurement. Potential values are reported in V *vs.* Fc^+/Fc. HOMO and LUMO energies were estimated from first oxidation/reduction half-wave potentials assuming an absolute ionization energy of 4.8 eV for ferrocene. We followed the IUPAC convention for CV plotting.

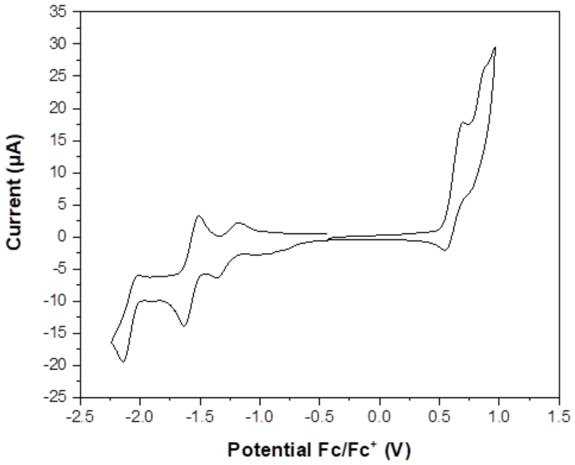

Figure S32. Cyclic voltammetry of **IFA**.

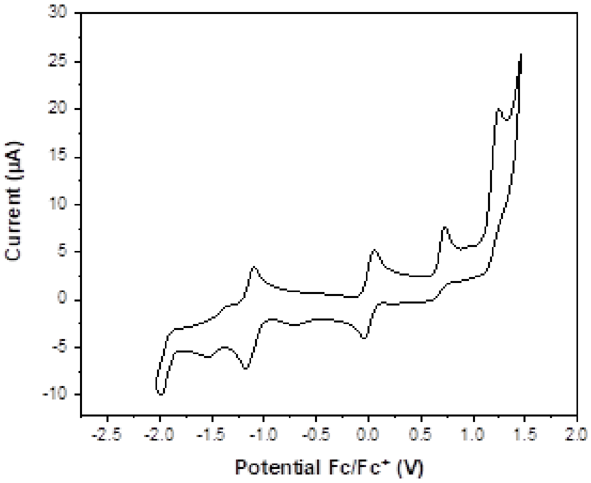

Figure S33. Cyclic voltammetry of **IFS**.

9. STM-BJ experiments

Sample preparation. Before the preparation of the samples, the substrates were cleaned with EtOH and flame-annealed. Later, they were immersed in a 10^{-3} M solution of the compound **IFA, IFS, IFSN** or **DH-IFS** in CH_2Cl_2 under N_2 for 15 minutes and dried with nitrogen gas. We used freshly cut gold wires (Goodfellow) as tip and commercial gold on quartz samples (Arrandee) as substrates.

Single-Molecule Conductance Studies. Single-molecule conductance (G) experiments were carried out using the scanning tunneling microscope break-junction (STM-BJ) technique. For these experiments, we used a home-built STM operating in air and room temperature. A constant 0.16 V bis voltage was applied between the STM tip and substrate electrodes along the experiments. A made-in-house linear current-to-voltage (*I-V*) converter with two stages of amplification was used for obtaining the current-distance (*I-z*) traces. The gains, of 10^8 V/A and 5×10^9 V/A respectively, were selected according to the conductance value observed for the compound under study. These values allowed us to explore a range in conductance $G=I/V$ of 8 orders of magnitude between 10 G_0 and 10^{-7} G_0. A protection resistor of 2×10^6 Ω was placed in-series with the STM circuit. Several runs of thousands (8000 to 20000) of conductance-distance (*G-z*) traces were collected while pulling the STM tip for this compound, changing to new tips, substrates or even different product batches in order to ensure the reproducibility of results. For **IFS** measurements, we synthesized the compound just before its use, kept it in CH_2Cl_2 solution under an inert atmosphere at –20°C. 1D and 2D histograms were constructed with the data of all of them together.

Data analysis. The data analysis started with the conductance-distance alignment of the traces. We used as origin for the z axis the point where the conductance of the traces reached to 0.5 G_0 (just after each gold contact is broken). As molecular junctions do not occur in all the G vs z traces, those presenting plateaus were separated from the ones without plateaus using an automatized program. This process gave rise to rates of success (percentage of traces containing plateaus) between 5% and 35%. The criterion for considering a trace containing plateau is that, at any conductance below 0.5 G_0, a displacement Δz larger than 0.1 nm is needed to produce a change in conductance of $\Delta\log(G/G_0) = 0.1$. As reference, the typical displacement needed for that conductance change for gold−gold tunnelling in air is 0.02 nm.

1D and 2D conductance histograms shown in Figure 2 in the main text were built from all the traces displaying plateaus. We built 1D histograms by accumulating the number of points N_{points} measured in fixed $\log(G/G_0)$ intervals from all the traces displaying plateaus. The y-axis in these histograms is normalized, as

$$N_{\text{norm}} = N_{\text{points}} / (N_{\text{curves}} \cdot v_{\text{p}} \cdot \Delta\log(G/G_0))$$

where N_{curves} is the total number of G vs z traces included in the histogram, and v_p is the number of points recorded per unit of length in z. With this normalization, N_{norm} is now just the inverse of the typical log(G/G_0) vs z curve, that tells us the distance z that we would need to move to produce a variation of 1 order of magnitude in conductance G, at each particular point. Therefore, it has units of nanometers per order of magnitude that we abbreviate to nm/order-mag. At those conductance values where the G vs z traces display plateaus, there will be a larger number of points recorded, and a peak will form in the 1D conductance histograms. The main value of the conductance peak observed in the 1D histogram is considered as the typical conductance of the molecular junction.

We built 2D conductance-distance histograms by accumulating the number of points N_{points} measured in fixed log(G/G_0) and z intervals from the traces. In this case, a color scale from green to brown was used for displaying the N_{points}, where green represents the G vs z intervals with fewer number of points and brown represents the G vs z intervals with higher number of points.

The plateau length was calculated as the variation of z (Δz) needed to change the junction conductance from 0.5 G_0 to a conductance value below the peak observed in the corresponding conductance histogram.

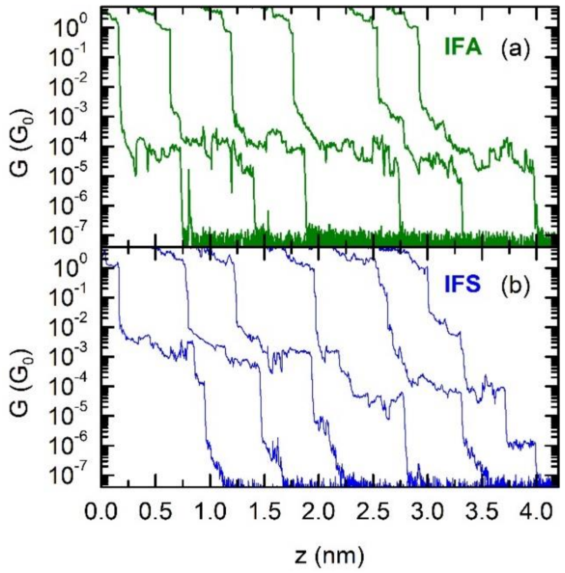

Figure S34. Individual G-z traces recorded for **IFA** and **IFS**.

9.1 Molecules for comparison

Table S1. Molecules used for comparison in the main text.

Compound	Structure	Conductance value	Reference
**DHIF-SMe**	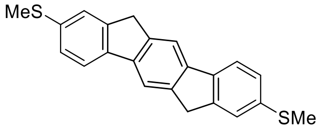	log(G/G_0)= −3.1	*Nat. Commun.* **2022**, *13*, 2102
**IFA**	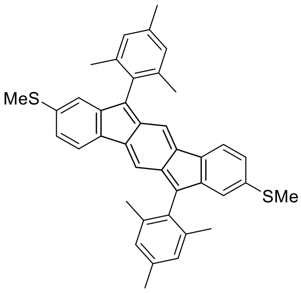	log(G/G_0)= −3.8	*This work*
**IFS**	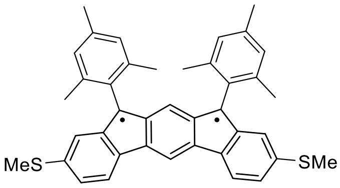	log(G/G_0)= −3.0	*This work*
**IFSN**	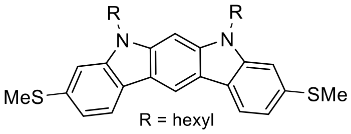	log(G/G_0)= −3.7	*This work*
**DH-IFS**	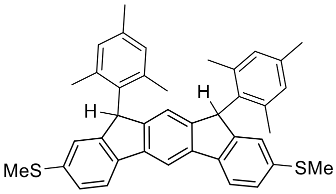	log(G/G_0)= −4.3	*This work*

9.2 Depiction of curly-arrow rules (CAR)

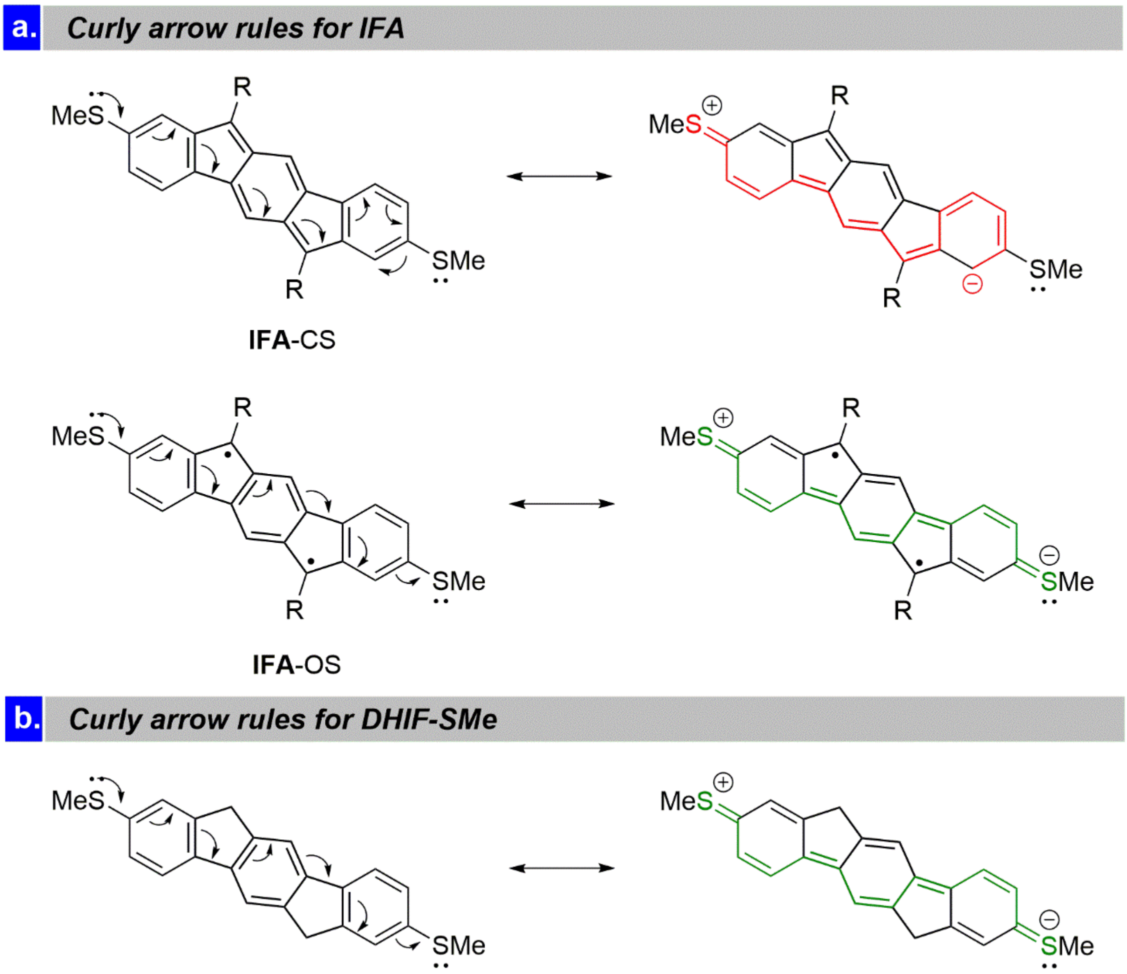

Figure S35. a) Curly arrow diagrams for **IFA**. In the CS configuration (our case) there is no direct conjugation between S atoms (indicative of DQI) whereas in the OS form there would be direct conjugation between anchor groups; b) Curly arrows for **DHIF-SMe**. There is direct conjugation between S atoms. R= 2,4,6-trimethylphenyl

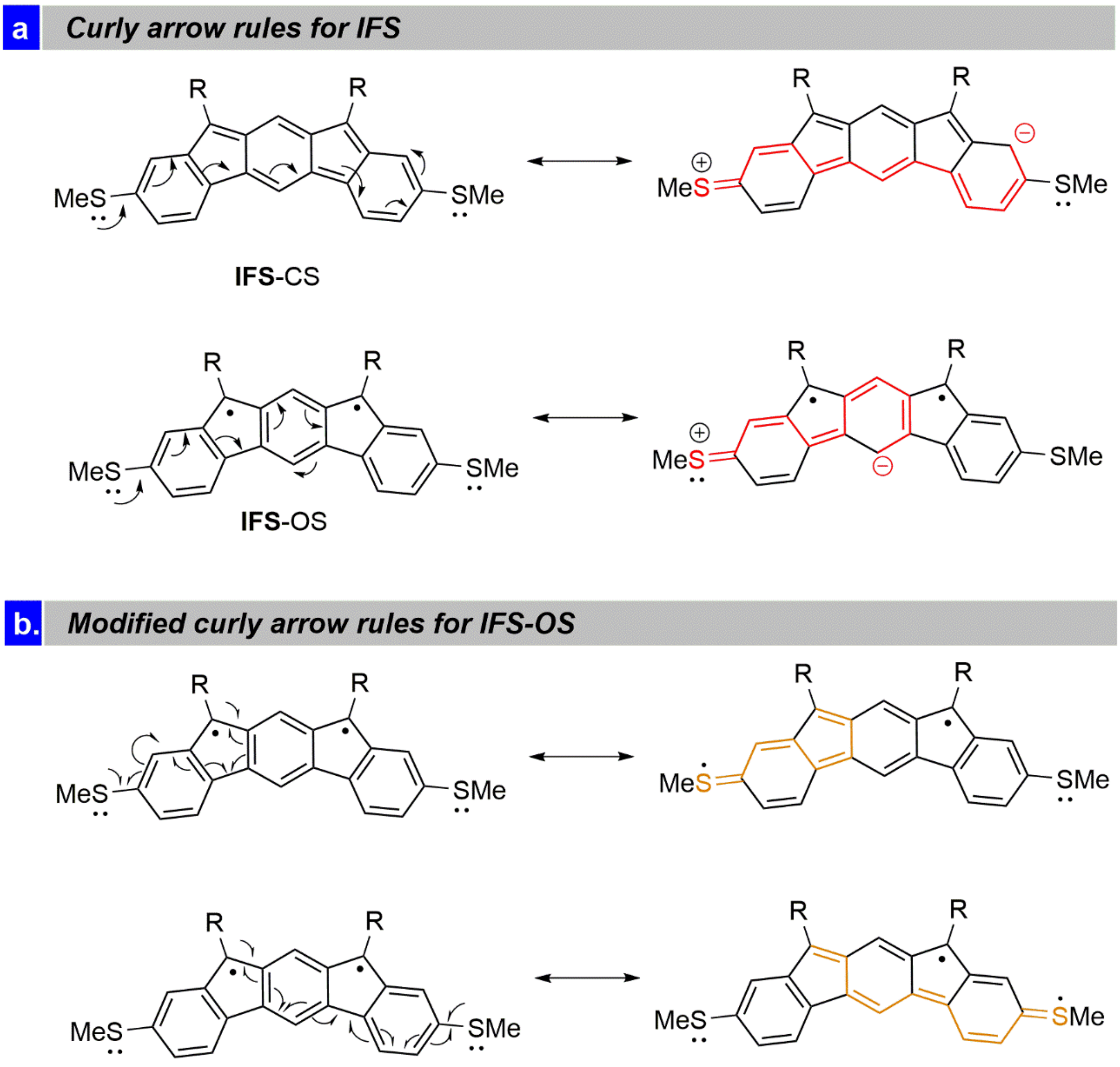

Figure S36. a) Curly arrow diagrams for **IFS**. Neither in the CS nor in the OS configuration would be direct conjugation between anchor groups; b) Modified curly arrows for **IFS-OS**. Unpaired electrons are able to repair the breaking of conjugation between S atoms alleviating the effects of DQI (indicative of sDQI). R= 2,4,6-trimethylphenyl

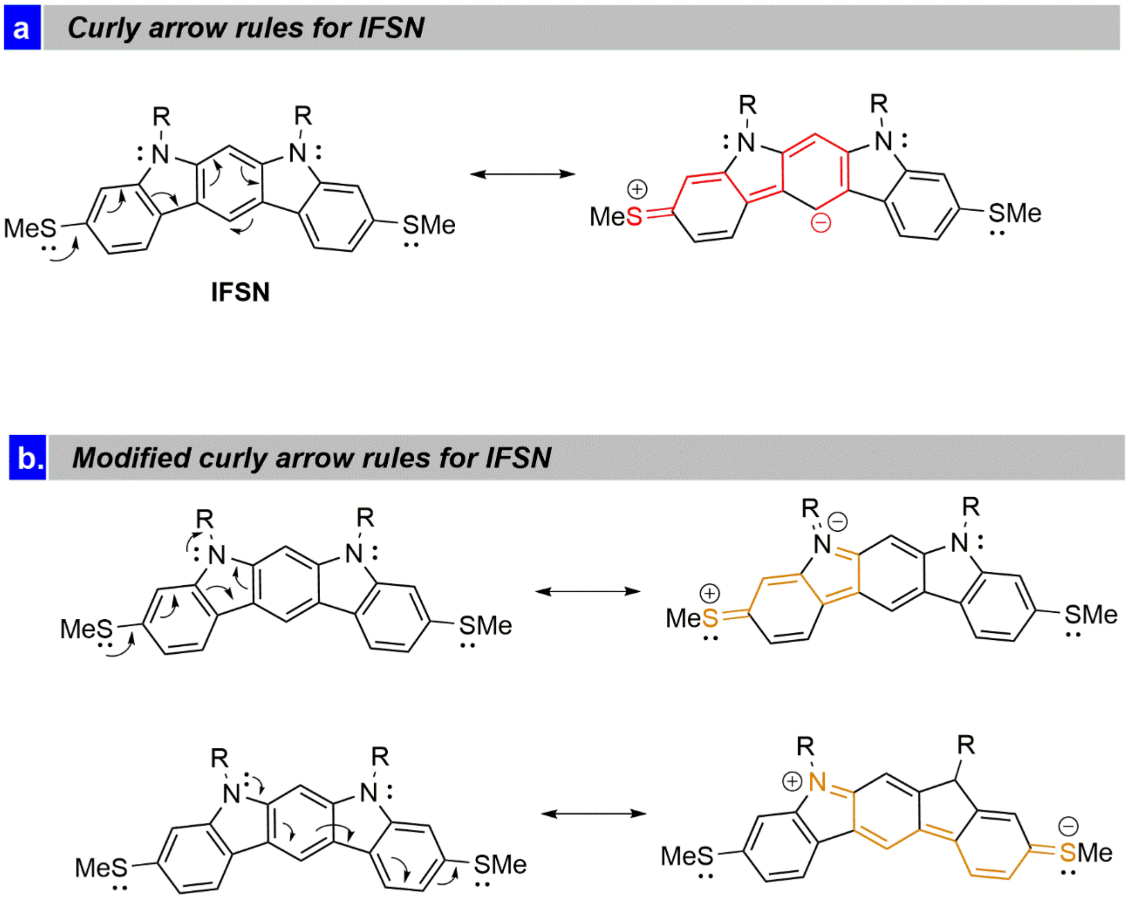

Figure S37. a) Curly arrow diagrams for **IFSN**. There is no direct conjugation between S atoms; b) Modified curly arrows for **IFSN**. Nitrogen lone pair is able to repair the breaking of conjugation between S atoms alleviating the effects of DQI (indicative of sDQI). R= *n*-hexyl

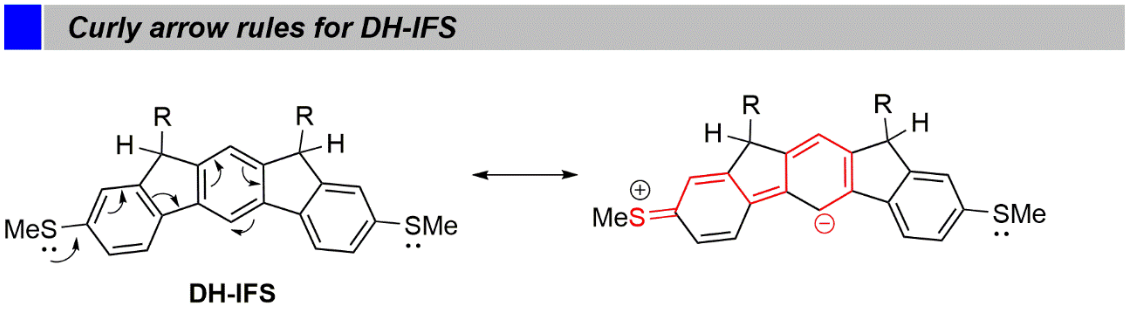

Figure S38. a) Curly arrow diagrams for **DH-IFS**. There is no direct conjugation between S atoms. R= 2,4,6-trimethylphenyl

9.3 Low conductance signals and time evolution in the break-junctions experiments of IFS

As mentioned in the main text, we found that the half-life of compound **IFS** in solution was limited, and, for this reason, we performed our break-junction experiments right after the samples were prepared. As shown in Figure 3, we observed a clear dominant peak centered at log(G/G_0)= −3.0 which was well reproducible in different experimental runs. However, we always observed some minority signals below log(G/G_0)= −4, which were variable and probably due to the partial decomposition of **IFS**. Figure S39 shows the result of separating the traces used in Figure 2 for **IFS** into traces with high and low conductance plateaus.

For this separation, we used a combination of the automatized process described above to identified traces with plateaus, with a clustering analysis based in an unsupervised k-means algorithm similar to those described in literature.[S7] We separated the traces displaying plateaus above (**IFS_a**), below (**IFS_b**) or both above and below (**IFS_c**) log(G/G_0)= –4. The 2D histograms of each group are shown in Figure S39 and the corresponding 1D histograms in Figure S40. The percentage respect to all traces with plateaus of each group was 60% for **IFS_a**, 30% for **IFS_b** and 10% for **IFS_c**.

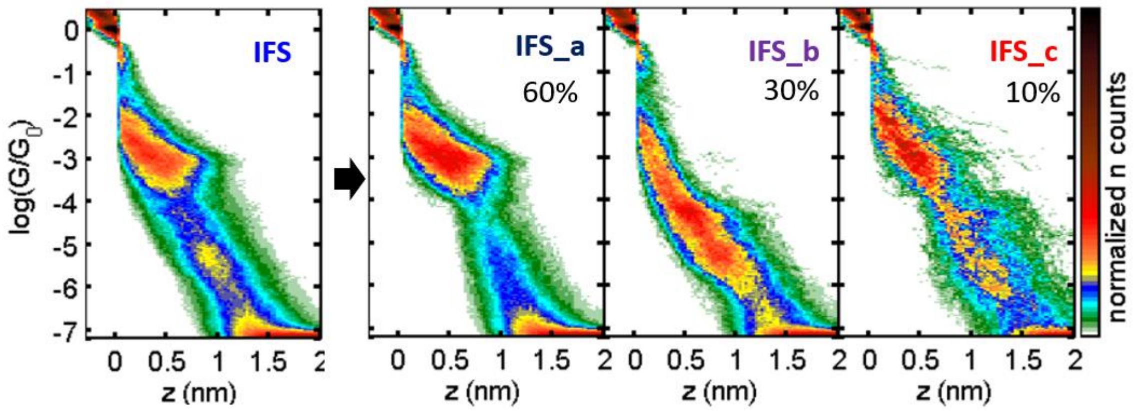

Figure S39. Left panel: 2D histogram of all traces with plateaus for **IFS** recorded from a freshly synthesized **IFS** sample. Right panel: 2D histograms of the traces with plateaus above (**IFS_a**), below (**IFS_b**) or both above and below (**IFS_c**) log(G/G_0)= -4.

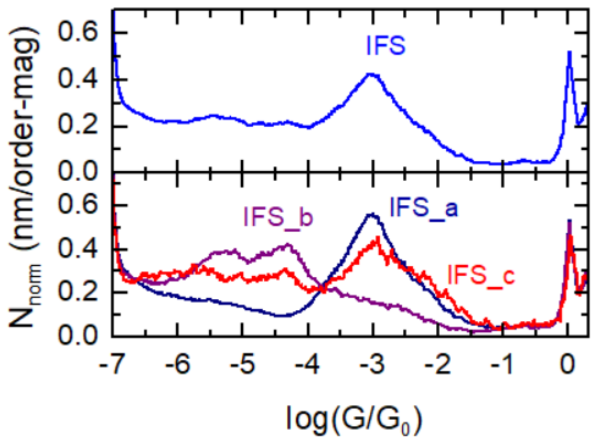

Figure S40. Corresponding 1D histograms for the traces used in the 2D histograms of Figure S39: (a) 1D histogram of all traces with plateaus for **IFS**. (b) 1D histograms of the traces with plateaus above (**IFS_a**), below (**IFS_b**) or both above and below (**IFS_c**) log(G/G_0)= −4.

As mentioned, the low plateau region changed in different experimental runs. Figure S41 shows several experimental runs for **IFS** different from that used in Figure 3. In some of the experiments, there is a predominance of the low conductance plateaus suggesting that the decomposition of **IFS** was more advanced.

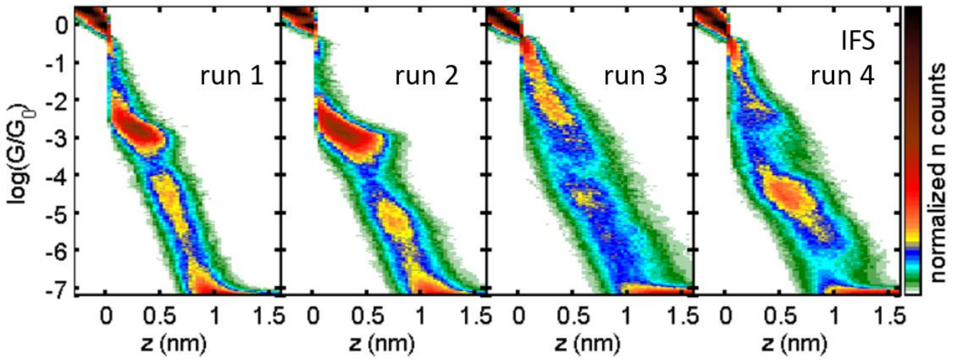

Figure S41. 2D histograms of **IFS** for different experimental runs.

In addition, we explored the evolution of the observed plateaus along time in an experimental run. Figure S42 shows how the measurement of Figure 3 evolved during 48 h (only the results of the first 24 hours were used in Figure 3). We observe that the shape of the dominant conductance cloud (centered at log(G/G_0)= −2.9) changes with time, becoming progressively less defined. On the other hand, we did not observe a significant change in the percentage of traces with plateaus with respect to the total number of recorded traces (from 40% to 33% in 48 h), neither in the percentage of traces with high plateaus with respect to the number of traces with plateaus (indicated in the labels of Figure S42). These results suggest that, while **IFS** decomposes at ambient conditions when it is in solution, it stabilizes when in contact with the gold surface, allowing to get stable and reproducible recordings during several hours.

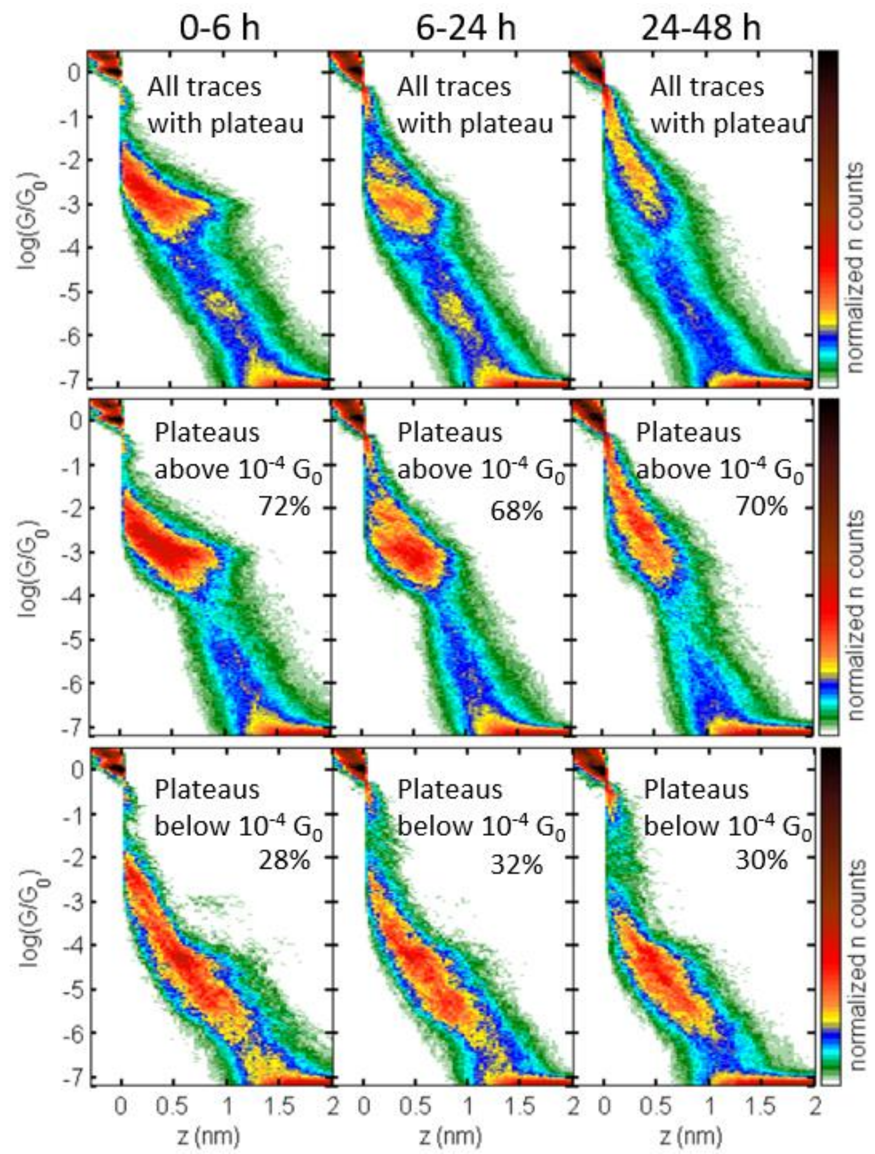

Figure S42. 2D histograms showing the evolution of the signal of **IFS** with the time.

9.4 Identification of the low-G signal. Comparison with DH-IFS signal.

As mentioned above, for **IFS**, and additional cloud of conductance plateaus was also observed below log(G/G_0)= −4. The origin of multiple conductance signals in molecular junction are often attributed to different factors, such as several binding modes[S8] or sites,[S9] multiple molecules in the junction,[S10] π-stacked molecules,[S11] or chains formed from the extraction of adatoms.[S12] These possibilities seem unlikely in our systems, as for structurally similar **IFA** we observed only one conductance group. However, there is also evidence of radicals acting as anchors to gold in molecular junctions[S13] or on metal surfaces.[S14] Here, the significant steric hindrance caused by the mesityl groups in **IFS** will, most likely, prevent efficient binding of the radical with the electrodes. Thus, given its minor contribution to the total signal, we suggest that the low-G signal

originates from a minor decomposition product already present in the solution of **IFS**. Indeed, this is the only signal observed when using substrates prepared with an old batch of **IFS**.

In order to test whether a decomposition product is responsible for the low-G signal, we compare it with the signal obtained for compound **DH-IFS** (Fig. 2 in the main text and Fig. S43, top), which is the synthetic precursor to **IFS**. Compound **DH-IFS** contains a sp^3 saturated carbon at the bridge locations making it the closest analogue of a decomposition product of **IFS** coming from the quenching of the radicals. As shown in the main text, and in contrast to **IFS**, the 1D and 2D histograms (Fig S43) display a single conductance group at $\log(G/G_0)= -4.3$ with a HWHM of 0.4. This result closely matches one of the peaks observed for **IFS** and is half an order of magnitude lower than that of **IFA**. Indeed, from the calculated transmission functions, a value close to $\log(G/G_0)= -3.2$ is obtained for **DH-IFS** (see Figure S49), a significantly lower value than for **IFA**. These findings provide further evidence that the low-G signal from the measurement of **IFS** originates from non-radical species, which are likely decomposition products of **IFS**.

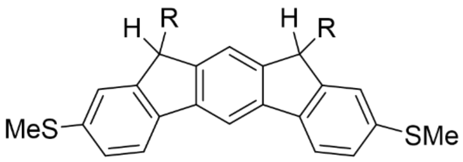

DH-IFS

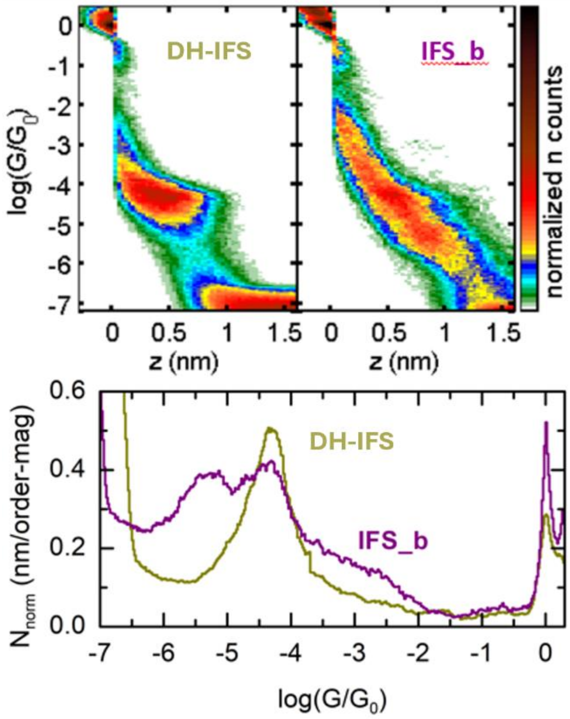

Figure S43. 2D and 1D histograms of compound **DH-IFS** in comparison with the corresponding histograms for the traces with low-G plateaus recorded for **IFS**. For **DH-IFS**, plateaus were identified in 21% of the recorded.

10.Theoretical calculation methods and results

The Gaussian 16 package,[S15] within the density functional theory (DFT), was employed to obtain the molecule optimized geometries, the energy and electron density distribution of molecular orbitals, the diradical character (y), spin density redistributions, and singlet-triplet energy gaps in the gas phase. The resulting calculations were then processed using the graphical interface program Gaussview 6.0.[S16]

The optimized geometries, electronic structures and the transmission spectra of gold-molecule-gold junctions were simulated by means of the widely used non-equilibrium Green's function (NEGF) formalism coupled to the DFT method, as implemented in the SIESTA, TranSIESTA and TBtrans codes (Version 4.1.5).[S17]

Spin distribution maps in the junction were extracted using the program Xcrysden.[S18]

10.1 Gas phase quantum chemical simulations

The geometries of the closed-shell singlets (CS) and open-shell (singlet, OS and triplet, T) states, were optimized using the Becke's three-parameter B3LYP exchange-correlation functional, and the 6-311+G(d,p) basis set. Geometries were verified as stationary points by harmonic vibrational frequency calculation at the same level of theory. Unrestricted approach was employed in open-shell optimizations, and the Broken Symmetry (BS) method additionally on OS.

The degree of open-shell character (y) was computed within the single determinant UDFT method.[S19] Here, y is defined as the occupation number of the lowest unoccupied natural orbital (LUNO), in the unrestricted wavefunction, denoted as n_{LUNO}:

$$y = n_{LUNO} = 2 - n_{HONO}$$

The range values for y, falls between 0, indicating a molecule with a closed-shell structure, and 1, representing a pure open-shell (diradical) structure.

The long-range corrected UDFT method with the Lee-Yang-Parr correlation functional (LC-BLYP) and the 6-311+G(d,p) basis set were employed. The value for the range separation parameter, μ, was set to 0.33 bohr^{-1}.

Table S2. Total and relative energies [eV] for the singlet and triplet states of IF compounds, as well as the singlet-triplet energy gap, ΔE_{S-T}.

Compound	$S_0(CS)^a$	$S_0(OS)^b$	T^c	ΔE(CS-OS)	$\Delta E(T-S_0)$/ ΔE_{S-T}
**IFA**	-63750.499	-63750.499	-63749.691	0.00	0.808
**IFS**	-63750.499	-63750.008	-63749.960	0.491	0.048

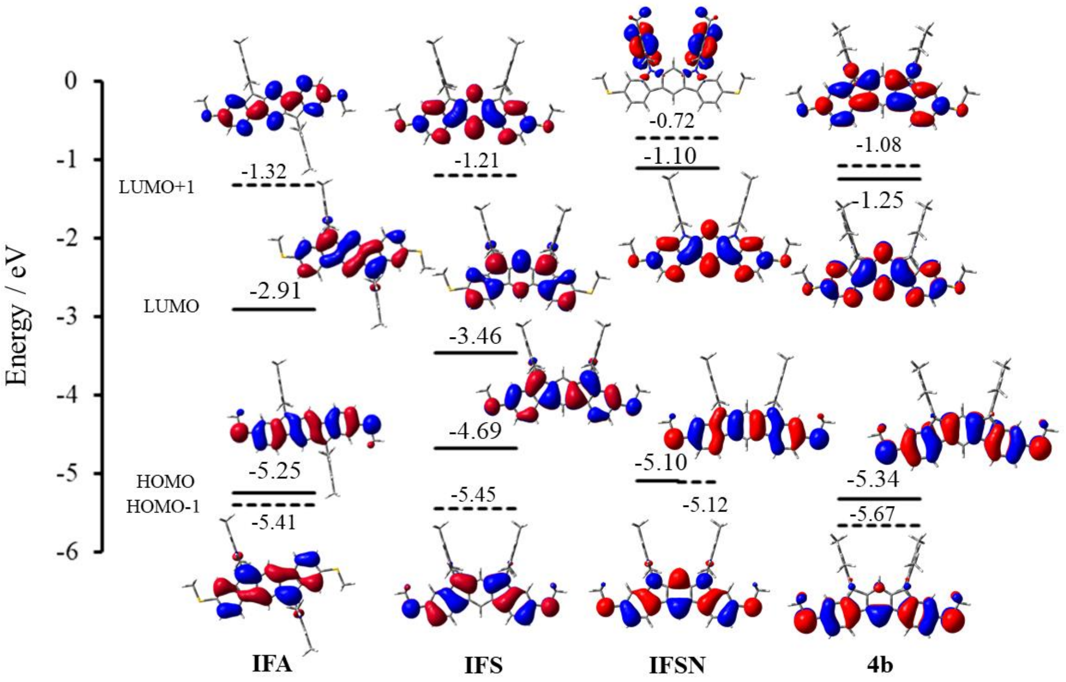

Figure S44. DFT-calculated molecular orbital energy diagram for **IFA**, **IFS, IFSN** and **DH-IFS** in the CS state. For simplicity, only the syn isomer of **DH-IFS** is shown. Isovalue of 0.02.

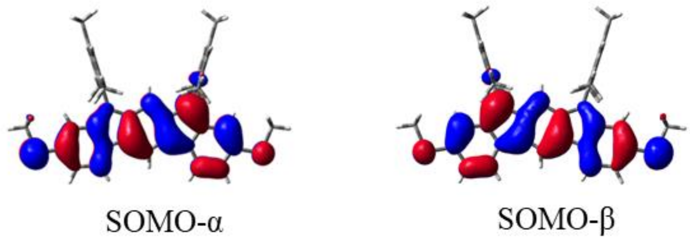

Figure S45. DFT-calculated SOMO corresponding to α and β electrons for **IFS** in the OS state. Isovalue of 0.02.

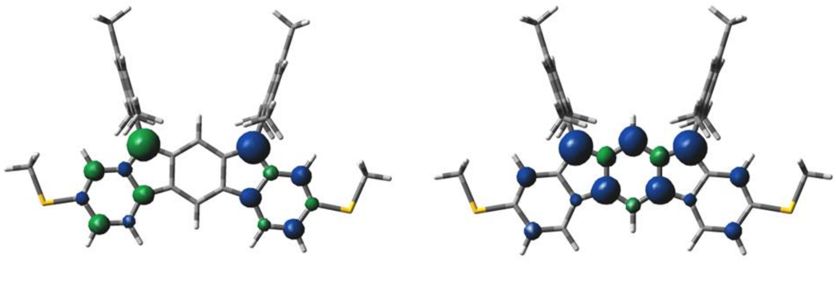

Figure S46. Spin density distribution in the diradical singlet ground state (OS: left), and in the triplet state (T: right) of **IFS** using an isovalue of 0.005. Blue and green surfaces correspond to α and β spin densities, respectively.

10.2 First-principles electron transport calculations

Single-molecule junctions have been described by a supercell containing the molecule, and two 10-atom Au tips (pyramids) attached to three Au(111) layers on the left and right side, respectively. An 8x8 in plane periodicity (64 atoms of Au per layer) was used for obtaining single-molecule properties (see Figure S47)

Geometries were optimized using the SIESTA code,[S17a] allowing to fully relax the molecule and the bottom and up-tips, while the first three gold layers of the bottom electrode were kept at bulk values, and the three gold layers of the top electrode were rigidly enabled to relax only along the transport direction (z in this case). This approach allows the full optimization of the junction: molecule, molecule-gold contacts, and the junction length, simultaneously as previously.[S20]

Kohn-Sham self-consistent DFT method using the Perdew-Burke-Ernzerhof (PBE) exchange-correlation functional within the Generalized Gradient Approximation (GGA) were employed.[S21] The valence electrons were described using a single-ζ plus polarization basis set for Au atoms, and double-ζζ plus polarization basis set for the molecule atoms, whereas norm-conserving pseudopotentials were used for the description of the core electrons. 300.00 Ry for real-space integrations and a 0.04 eV/A as maximum force tolerance in coordinate optimization were established, and the transverse Brillouin zone was described with a 2x2x1 k-grid sampling.

Spin-polarized calculations were performed in open-shell molecular states, defining as the initial spin density for each carbon atom, that obtained from the gas phase optimization. Besides, spin-unpolarized calculations were carried out for singlet closed-shell states.

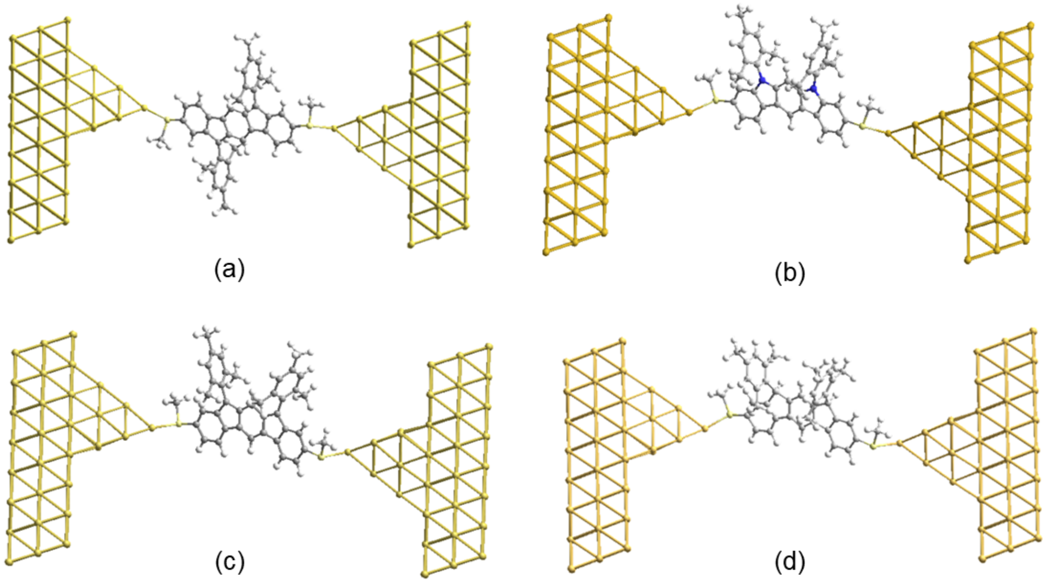

Figure S47. Optimized molecular junction geometries used for calculating the transmission spectra (a) **IFA-CS**, (b) **IFSN-CS**, (c) **IFS-OS** and (d) **DH-IFS-syn**. Color code: Au (dark yellow), C (grey), S (yellow), N (blue) and H (black). The xyz coordinates are shown in the next section 10.3.

After geometry optimization, two semi-infinite Au (111) electrodes were included on both sides of the previous structure. For an accurate description of the semi-infinite electrode bulk properties, T_{RAN}SIESTA [S17b,S17c] calculations were performed with high k-point sampling along the transport direction (80 k-points). Then, the electronic structure of the open system (scattering region between semi-infinite electrodes) was resolved using the T_{RAN}SIESTA module, employing identical functional, pseudopotentials, and k-grid sampling parameters as those used during the geometry optimization. TB_{TRANS} post-processing code,[S17b,S17c] was then used to calculate the transmission coefficient T(E) for electrons of energy E, traveling through the molecule from the left to the right electrode at zero-bias. In spin-polarized calculations, $T^{\sigma}(E)$ corresponds with the transmission coefficient for electrons of energy E, and Spin σ=[up,down], from the left to the right electrode across the molecule.

Double-ζζ, and double-ζζ plus polarization basis set were used for gold and molecule atoms, respectively. k-point sampling was increased to 8x8x1 to reach the convergence of the transmission functions. In addition, zero-bias conductance is approximated as the transmission probability at the Fermi level, $G/G_0 = T(E_F)$, according to the Landauer approach for linear regimen and low temperatures ($G_0 = (2e^2)/h$, the quantum conductance).

For open-shell states, the average between spin-up (σ= ↑) and spin-down (σ=↓) transmission coefficients are considered, and the conductance will be given by the average value between spin-up and spin-down conductances:

$$G=(G^{\uparrow} + G^{\downarrow})/2$$

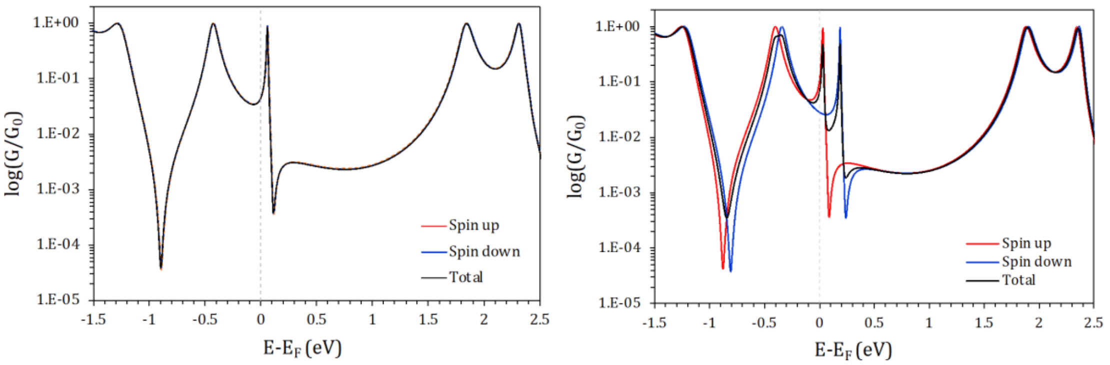

Figure S48. Spin-dependent and total transmission functions for the open-shell states of the **IFS** molecule: OS (left) and T (right). The spin-up and spin-down functions in **IFS-OS** overlap.

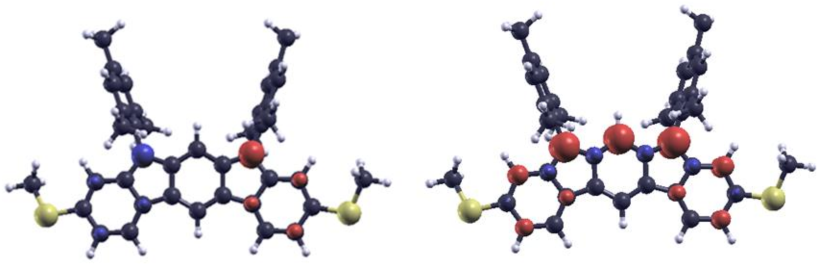

Figure S49. Spin density distribution calculated for **IFS** in OS (left, grid value:10^{-4}), and T (right, grid value: 10^{-3}) states in the junction.

10.2.1 Description of the interference phenomena in the transmission functions of IFA, IFS-OS and IFSN

Close to the Fermi level (E_F), the zero-bias transmission function of **IFA** (Figure 5), exhibits a distinct anti-resonance dip (destructive) at –0.12 eV, succeeded by a peak at 0.16 eV, which corresponds to the LUMO molecular level resonance (energy difference of 0.30 eV). This interference pattern have been previously studied, and associated with molecular cross-conjugation or the existence of side-groups, different anchoring sites in PAH molecules to electrodes, or the inability to draw resonances structures between the anchoring groups.[S22] In the case at hand, stemming from the latter assumption, absence of direct conjugation between anchors (Figure S35a), as detailed in the experimental section.

On the other hand, the transmission for the **IFS**-**OS** presents a characteristic Fano-resonance profile adjacent to E_F, with sharp peak at 0.06 eV corresponding to the LUMO level resonance,

and a sharp dip at, 0.12 eV. The energy difference between both resonances is lower than 0.1 eV. Fano resonance arises when a bound state is coupled to a continuum of states. In the context of single-molecule junctions, this resonance has been ascribed to the interplay between discrete and continuous molecular levels, as well as the electron density distribution that drives to the previous phenomenon.[S23] Furthermore, a shifted DQI (s-DQI) is observed between the HOMO and (HOMO-1) resonances, and far enough from E_F to contribute on the zero bias conductance. This support the idea that unpaired electron from radicals enable the drawing of resonance structures connecting them with the anchor groups, following curly-arrow rules (CARs) (see Figure S36b).

Additionally, **IFSN** exhibits s-DQI within the HOMO-LUMO gap, similar to **IFA**, but towards the HOMO level**.** This indicates that the lone pairs of nitrogen help to overcome the lack of direct conjugation between anchors (See Figure S37b), although less effectively than the unpaired electrons from radicals in **IFS,** where the s-DQI is outside the gap.

10.2.2. Energy-resolved transmission spectra for DH-IFS isomers

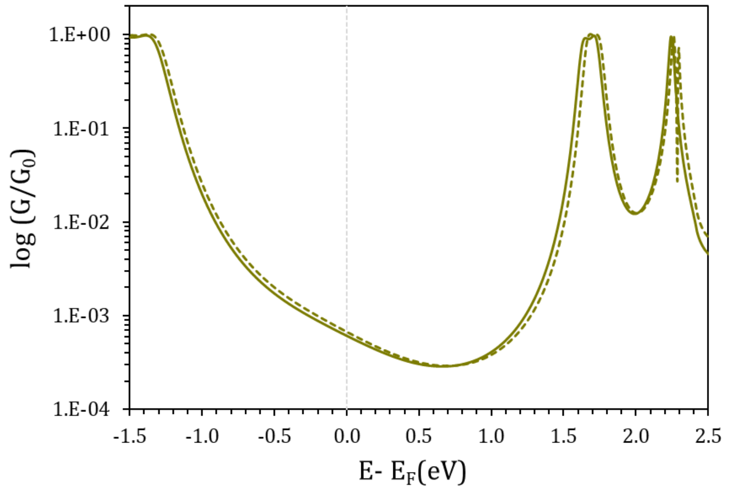

Figure S50. Zero-bias transmission function for **DH-IFS-syn** isomer (continuum line) and **DH-IFS-anti** isomer (dashed line).

10.2.3. IFS Radical cation energy-resolved transmission function

Since NEGF-DFT formalism is not straightforward for calculating the transmission properties of charged molecules, particularly with gold electrodes serving as electron reservoirs, we adopted an alternative modeling approach. We placed eight fluorine atoms above and below the molecule core, at various distances (see Figure S51-Left), following the previous work of Xiaodong Yin, et. al. ("*A reversible single-molecule switch based on activated antiaromaticity*,"

Sci Adv., **2017**, 3(10): eaao2615).[S24] This method achieves charge neutrality for the positively charged molecule and negative countercharges, until the molecule reached a charge close to +1 e for the radical cation (RC) in gas phase. The molecule charge was determined using Hirshfeld atomic population analysis.

Spin-dependent DFT based transmission calculations were performed to capture resonances derived from the partially filled molecular orbitals of the radical cation around $E_{F,}$ Figure S51-Right and Figure S52.

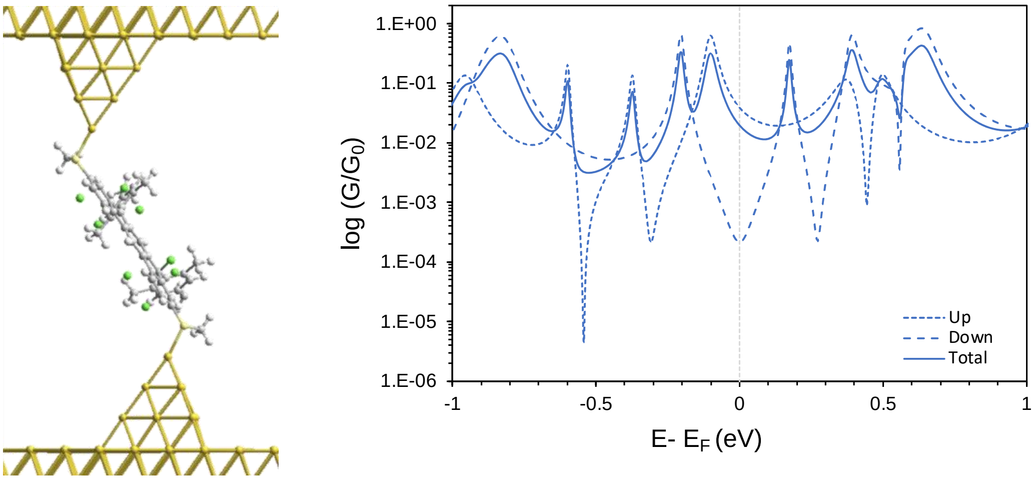

Figure S51. (Left) Molecule junction containing the neutral **IFS** molecule (C: grey, S: yellow, and H: white), and eight fluorine atoms (green) to model the **IFS** in a +1 oxidized state; (Right) Spin-dependent and total transmission as a function of the energy for the oxidized **IFS** molecule.

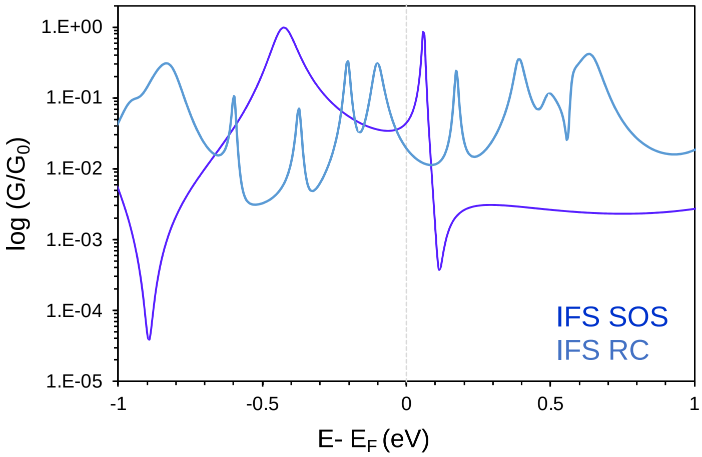

Figure S52. Comparison of **IFS OS** and **RC** energy-dependent transmission functions.

10.3. Optimized Geometries Coordinates

The following sections provide the atomic coordinates associated with (i) the optimized molecular geometries in the gas phase, considering the close- (CS) and open-shell (OS, T) states (section 10.3.1); (ii) the optimized -gold tips and molecule- structures extracted from the junction depicted in Figure S47, in their ground electronic states, and the triplet state of **IFS** (section 10.3.2).

10.3.1. Gas Phase

The relative energies are shown in Table S2

IFA-CS

C	1.37024600	0.29661900	0.00005700
C	0.89662000	-1.09229400	0.00014900
C	-0.43234500	-1.38308200	0.00009300
C	1.37024600	-0.29661900	-0.00005800
C	-0.89662000	1.09229400	-0.00015000
C	0.43234500	1.38308200	-0.00009400
H	-0.79649300	-2.40556900	0.00016000
H	0.79649300	2.40556900	-0.00016100
C	-2.75012400	-0.31208700	-0.00014100
C	-3.21718800	1.07730600	-0.00029100
C	-4.51515900	1.58544800	-0.00041400
C	-4.69566200	2.97548200	-0.00054600
C	-3.57732700	3.82745500	-0.00055200
C	-2.27976000	3.31479800	-0.00042800
C	-2.09089300	1.93838000	-0.00029600
H	-5.35688800	0.90513200	-0.00040600
H	-3.72724300	4.90149600	-0.00065400
H	-1.43662200	3.99743400	-0.00043500
S	-6.29006300	3.77395100	-0.00071300
C	-7.46846000	2.38662700	-0.00065900
H	-7.36587900	1.77284200	-0.89634200
H	-8.45608500	2.84879100	-0.00075800
H	-7.36598400	1.77300100	0.89514600
C	2.09089300	-1.93838000	0.00029600
C	2.75012400	0.31208700	0.00014100
C	3.21718800	-1.07730600	0.00029000
C	2.27976000	-3.31479800	0.00042800
C	4.51515900	-1.58544800	0.00041500
C	3.57732700	-3.82745500	0.00055300
C	4.69566200	-2.97548200	0.00054700
S	6.29006300	-3.77395100	0.00071600
C	7.46846000	-2.38662700	0.00065800
H	8.45608500	-2.84879100	0.00075700
H	7.36588000	-1.77284000	0.89634000
H	7.36598300	-1.77300300	-0.89514700
H	1.43662200	-3.99743400	0.00043500
H	3.72724300	-4.90149600	0.00065500
H	5.35688800	-0.90513200	0.00040700
C	-3.63724900	-1.50263100	-0.00009200
C	-4.06831900	-2.06076000	1.22008400
C	-4.06820500	-2.06094500	-1.22022400
C	-4.91746100	-3.16991200	1.19650300
C	-4.91734900	-3.17009300	-1.19655500
C	-5.35126100	-3.74255300	-0.00000300
H	-5.24928600	-3.59461700	2.13956300
H	-5.24908600	-3.59494100	-2.13958200
C	-3.62323000	-1.48027100	-2.54281100
C	-3.62347100	-1.47988500	2.54262500
C	-6.24227500	-4.96163000	0.00004700
H	-2.54198800	-1.57570600	2.67717900
H	-3.85681900	-0.41377300	2.61297200
H	-4.11264200	-1.99024500	3.37443700
H	-4.11231500	-1.99076500	-3.37459300
H	-3.85657900	-0.41417300	-2.61334800
H	-2.54173400	-1.57610500	-2.67724200
H	-5.64749700	-5.88181400	0.00012500
H	-6.88302500	-4.98761200	0.88466600
H	-6.88296900	-4.98772700	-0.88460900
C	3.63724900	1.50263100	0.00009200
C	4.06820500	2.06094500	1.22022300
C	4.06831900	2.06076000	-1.22008400
C	4.91734900	3.17009300	1.19655400
C	4.91746200	3.16991200	-1.19650300
C	5.35126100	3.74255300	0.00000300
H	5.24908500	3.59494100	2.13958100
H	5.24928700	3.59461600	-2.13956300
C	3.62322900	1.48027200	2.54281100
C	3.62347200	1.47988400	-2.54262600
C	6.24227500	4.96163000	-0.00004700
H	6.88302500	4.98761300	-0.88466600
H	5.64749700	5.88181400	-0.00012500
H	6.88296900	4.98772600	0.88460900
H	3.85682000	0.41377300	-2.61297300
H	2.54199000	1.57570500	-2.67718000
H	4.11264400	1.99024500	-3.37443800
H	3.85657800	0.41417300	2.61334700
H	4.11231400	1.99076600	3.37459200
H	2.54173200	1.57610600	2.67724100

IFA-OS

C	-1.37024700	0.29661400	0.00001800

C	-0.89661700	-1.09229700	0.00005700
C	0.43235000	-1.38308000	0.00004000
C	1.37024700	-0.29661400	-0.00001800
C	0.89661700	1.09229700	-0.00005700
C	-0.43235000	1.38308000	-0.00004000
H	0.79650100	-2.40556600	0.00006800
H	-0.79650100	2.40556600	-0.00006800
C	2.75012500	-0.31207700	-0.00004500
C	3.21718400	1.07731700	-0.00010400
C	4.51515400	1.58546400	-0.00014900
C	4.69565200	2.97549800	-0.00020200
C	3.57731400	3.82746700	-0.00020900
C	2.27974800	3.31480600	-0.00016400
C	2.09088600	1.93838700	-0.00011100
H	5.35688500	0.90515100	-0.00014200
H	3.72722600	4.90150900	-0.00025100
H	1.43660800	3.99743900	-0.00017100
S	6.29005000	3.77397300	-0.00026300
C	7.46845100	2.38665300	-0.00023600
H	7.36594200	1.77298000	0.89553200
H	8.45607500	2.84882100	-0.00027100
H	7.36590800	1.77291500	-0.89595500
C	-2.09088600	-1.93838700	0.00011100
C	-2.75012500	0.31207700	0.00004500
C	-3.21718400	-1.07731700	0.00010400
C	-2.27974800	-3.31480600	0.00016400
C	-4.51515400	-1.58546400	0.00014900
C	-3.57731400	-3.82746700	0.00020900
C	-4.69565200	-2.97549800	0.00020200
S	-6.29005000	-3.77397300	0.00026300
C	-7.46845100	-2.38665300	0.00023600
H	-8.45607500	-2.84882100	0.00027100
H	-7.36594200	-1.77298000	-0.89553200
H	-7.36590800	-1.77291500	0.89595500
H	-1.43660800	-3.99743900	0.00017100
H	-3.72722600	-4.90150900	0.00025100
H	-5.35688500	-0.90515100	0.00014200
C	3.63725400	-1.50261900	-0.00002200
C	4.06824900	-2.06087700	-1.22016500
C	4.06828800	-2.06080000	1.22014300
C	4.91739600	-3.17002400	-1.19651900
C	4.91743400	-3.16994800	1.19653900
C	5.35127300	-3.74253500	0.00002100
H	5.24916300	-3.59482900	-2.13955400
H	5.24923200	-3.59469400	2.13959100
C	3.62339700	-1.47998500	2.54269600
C	3.62331300	-1.48014600	-2.54274000
C	6.24229100	-4.96160900	0.00004500
H	2.54182200	-1.57598100	-2.67721100
H	3.85665900	-0.41404300	-2.61322100
H	4.11242900	-1.99059900	-3.37452900
H	4.11254100	-1.99038300	3.37450100
H	3.85674200	-0.41387700	2.61309900
H	2.54191000	-1.57581300	2.67721000
H	5.64751500	-5.88179500	0.00007700
H	6.88300300	-4.98767100	-0.88459800
H	6.88302300	-4.98762200	0.88467600
C	-3.63725400	1.50261900	0.00002200
C	-4.06828800	2.06080000	-1.22014300
C	-4.06824900	2.06087700	1.22016500
C	-4.91743400	3.16994800	-1.19653900
C	-4.91739600	3.17002400	1.19651900
C	-5.35127300	3.74253500	-0.00002100
H	-5.24923200	3.59469400	-2.13959100
H	-5.24916300	3.59482900	2.13955400
C	-3.62339700	1.47998500	-2.54269600
C	-3.62331300	1.48014600	2.54274000
C	-6.24229100	4.96160900	-0.00004500
H	-6.88300300	4.98767100	0.88459800
H	-5.64751500	5.88179500	-0.00007700
H	-6.88302300	4.98762200	-0.88467600
H	-3.85665900	0.41404300	2.61322100
H	-2.54182200	1.57598100	2.67721100
H	-4.11242900	1.99059900	3.37452900
H	-3.85674200	0.41387700	-2.61309900
H	-4.11254100	1.99038300	-3.37450100
H	-2.54191000	1.57581300	-2.67721000

IFA-T

C	1.35764000	0.29700800	0.01005500
C	0.91049500	-1.05805900	0.02017300
C	-0.44945400	-1.36142900	0.01226800
C	-1.35764000	-0.29700900	-0.01005800
C	-0.91049500	1.05805800	-0.02017500
C	0.44945500	1.36142800	-0.01227000
H	-0.80246600	-2.38728700	0.02422600
H	0.80246700	2.38728500	-0.02422700
C	-2.81616000	-0.30770900	-0.01276200
C	-3.24777900	1.05673200	-0.02660600
C	-4.54464700	1.59698100	-0.04822200
C	-4.70078900	2.98406800	-0.07054500
C	-3.56456900	3.81846300	-0.07374400
C	-2.27273400	3.28557200	-0.05688100
C	-2.10147300	1.90937000	-0.03397100
H	-5.39762600	0.93075100	-0.05042400
H	-3.69696400	4.89466100	-0.09278400
H	-1.42077300	3.95710400	-0.06439100
S	-6.27876800	3.81655000	-0.09704400
C	-7.48272800	2.45196300	-0.11682100
H	-7.37092100	1.83209100	-1.00729300
H	-8.46154100	2.93185100	-0.14108200
H	-7.41177100	1.84062400	0.78369100
C	2.10147300	-1.90937100	0.03397100
C	2.81616200	0.30770800	0.01276100
C	3.24778000	-1.05673300	0.02660700
C	2.27273500	-3.28557300	0.05688200
C	4.54464800	-1.59698100	0.04822400
C	3.56457000	-3.81846400	0.07374600
C	4.70079000	-2.98406700	0.07054700
S	6.27876900	-3.81655000	0.09704800
C	7.48273000	-2.45196300	0.11682000
H	8.46154300	-2.93185100	0.14108100
H	7.37092300	-1.83209000	1.00729100

H	7.41177200	-1.84062600	-0.78369200
H	1.42077500	-3.95710500	0.06439100
H	3.69696600	-4.89466100	0.09278700
H	5.39762800	-0.93075100	0.05042500
C	-3.67852800	-1.51016800	-0.00140800
C	-4.25747900	-1.95451700	1.20535200
C	-3.92503600	-2.21377400	-1.19878400
C	-5.06910400	-3.09132900	1.19126000
C	-4.74533000	-3.34365700	-1.16638200
C	-5.32471900	-3.80217800	0.01779300
H	-5.50988800	-3.43058900	2.12427300
H	-4.93920400	-3.87575400	-2.09331200
C	-3.33385600	-1.74792500	-2.50935900
C	-3.99949600	-1.22885300	2.50597100
C	-6.17830200	-5.04755100	0.03349400
H	-2.92814300	-1.14661800	2.71048400
H	-4.39614400	-0.20966900	2.48563900
H	-4.46419600	-1.75479900	3.34211500
H	-3.67018900	-2.37989900	-3.33345500
H	-3.62223600	-0.71663400	-2.73263300
H	-2.24046300	-1.77298400	-2.48934900
H	-5.56549400	-5.94065700	0.19893600
H	-6.92339000	-5.01079400	0.83173700
H	-6.70297500	-5.18361400	-0.91522700
C	3.67852800	1.51016800	0.00140700
C	3.92505000	2.21376400	1.19878600
C	4.25746400	1.95452800	-1.20535600
C	4.74534300	3.34364800	1.16638300
C	5.06908700	3.09134100	-1.19126400
C	5.32471600	3.80218000	-0.01779400
H	4.93922700	3.87573800	2.09331600
H	5.50986000	3.43060900	-2.12428000
C	3.33388500	1.74790300	2.50936400
C	3.99946700	1.22887400	-2.50597800
C	6.17829600	5.04755500	-0.03349500
H	6.92338600	5.01079800	-0.83173700
H	5.56548800	5.94065900	-0.19893900
H	6.70296900	5.18362000	0.91522600
H	4.39610800	0.20968600	-2.48565400
H	2.92811200	1.14664600	-2.71048300
H	4.46416300	1.75482200	-3.34212300
H	3.62227300	0.71661200	2.73262900
H	3.67022100	2.37987400	3.33346000
H	2.24049200	1.77295500	2.48936400

IFS-CS

C	-1.20023000	0.54144600	0.00482200
C	-1.20061200	1.98548800	0.00350900
C	0.00000100	2.69513900	0.00000100
C	1.20061000	1.98549100	-0.00350700
C	1.20022900	0.54145000	-0.00482000
C	-0.00000300	-0.17679200	0.00000100
H	0.00000000	3.78139500	0.00000000
H	-0.00000200	-1.26304000	0.00000100
C	-2.57538900	2.39376600	0.00484500
C	-2.54991100	0.06746500	0.00646400
C	-3.39377100	1.20662200	0.00603600
C	-3.20453300	3.64868100	0.00598000
C	-4.80551900	1.29644800	0.01042200
C	-4.58423400	3.71687900	0.00896300
C	-5.39731900	2.54555700	0.01159800
S	-7.15554500	2.84412200	0.01709500
C	-7.86424600	1.16905300	0.02483000
H	-8.94541000	1.30985500	0.02989500
H	-7.57422400	0.61749900	0.92024700
H	-7.58334500	0.61282500	-0.87061000
H	-2.62306700	4.56419200	0.00501500
H	-5.06945900	4.68731500	0.01010200
H	-5.39202300	0.38667400	0.01358600
C	2.57539200	2.39377000	-0.00484400
C	2.54990300	0.06746900	-0.00646200
C	3.39377000	1.20662800	-0.00603500
C	3.20453600	3.64868300	-0.00598000
C	4.58424000	3.71688000	-0.00896400
C	4.80551600	1.29644900	-0.01042200
H	5.39201800	0.38667400	-0.01358500
C	5.39732100	2.54555800	-0.01159800
H	2.62307200	4.56419500	-0.00501500
H	5.06946500	4.68731500	-0.01010400
S	7.15554900	2.84411400	-0.01709700
C	7.86424200	1.16904200	-0.02482800
H	8.94540700	1.30983900	-0.02989600
H	7.58334000	0.61281700	0.87061300
H	7.57421700	0.61748600	-0.92024300
C	2.98708300	-1.34993600	-0.00823800
C	3.14622300	-2.03862300	-1.22806800
C	3.25648200	-2.00691600	1.20987100
C	3.56617900	-3.37087900	-1.20555900
C	3.67276100	-3.34014100	1.18443100
C	3.83069000	-4.04223900	-0.01122200
H	3.69165700	-3.89521700	-2.14850900
H	3.87955500	-3.84108500	2.12579100
C	-2.98708900	-1.34994000	0.00823900
C	-3.14622300	-2.03863000	1.22806800
C	-3.25648700	-2.00692000	-1.20987100
C	-3.56617400	-3.37088700	1.20555800
C	-3.67276100	-3.34014700	-1.18443200
C	-3.83068400	-4.04224700	0.01122000
H	-3.69164800	-3.89522700	2.14850800
H	-3.87955300	-3.84109000	-2.12579200
C	2.87845700	-1.35611300	-2.54978900
C	3.10234700	-1.29126500	2.53216100
C	4.24866600	-5.49315100	-0.01103000
C	-3.10235700	-1.29126700	-2.53216000
C	-2.87845900	-1.35612000	2.54978900
C	-4.24865500	-5.49316000	0.01102600
H	-4.88602900	-5.72481600	-0.84567200
H	-3.37520400	-6.15240700	-0.04359000
H	-4.79553400	-5.75103200	0.92107000
H	3.37521500	-6.15240100	0.04355100
H	4.79557400	-5.75101100	-0.92105900
H	4.88601300	-5.72481300	0.84568600
H	-3.10752700	-2.02258400	3.38344000
H	-1.83183400	-1.05118400	2.63926400

H -3.48078300 -0.45032600 2.66229200
H -3.75691400 -0.41721200 -2.59416900
H -2.08019900 -0.92933600 -2.67502200
H -3.34514600 -1.95599600 -3.36330900
H 2.08018500 -0.92934500 2.67502500
H 3.34514500 -1.95599200 3.36331000
H 3.75689600 -0.41720400 2.59416900
H 1.83182600 -1.05119500 -2.63927000
H 3.48076600 -0.45030800 -2.66228400
H 3.10754200 -2.02256900 -3.38344000

IFS-OS

C -1.19911600 0.52503300 -0.00205100
C -1.19769800 1.96394500 -0.00158800
C 0.00000000 2.67691900 0.00000000
C 1.19769800 1.96394500 0.00158900
C 1.19911600 0.52503300 0.00205100
C 0.00000000 -0.19460300 0.00000000
H 0.00000000 3.76261800 0.00000000
H 0.00000000 -1.28022400 0.00000000
C -2.58195800 2.38632100 -0.00253500
C -2.55841500 0.05701400 -0.00288900
C -3.40182700 1.21023200 -0.00307800
C -3.19328400 3.64194500 -0.00343900
C -4.80617900 1.30768300 -0.00536100
C -4.57913700 3.72724900 -0.00513600
C -5.39390900 2.56758400 -0.00626400
S -7.15234500 2.86348400 -0.00902500
C -7.86484000 1.18932700 -0.01303700
H -8.94546900 1.33457300 -0.01569500
H -7.58480300 0.63318000 0.88256600
H -7.58000800 0.63558200 -0.90861200
H -2.60260900 4.55170400 -0.00308000
H -5.05334900 4.70281200 -0.00597300
H -5.40021400 0.40289400 -0.00671100
C 2.58195800 2.38632100 0.00253500
C 2.55841500 0.05701400 0.00288900
C 3.40182700 1.21023200 0.00307800
C 3.19328400 3.64194500 0.00343900
C 4.57913700 3.72724900 0.00513600
C 4.80617900 1.30768300 0.00536100
H 5.40021400 0.40289400 0.00671100
C 5.39390900 2.56758400 0.00626400
H 2.60260900 4.55170400 0.00308100
H 5.05334900 4.70281100 0.00597300
S 7.15234500 2.86348300 0.00902500
C 7.86484000 1.18932700 0.01303700
H 8.94546900 1.33457300 0.01569500
H 7.58000800 0.63558200 0.90861300
H 7.58480300 0.63318000 -0.88256600
C 3.00357100 -1.35708600 0.00314200
C 3.24603000 -2.02085000 -1.21660000
C 3.19442000 -2.03702600 1.22308200
C 3.67017000 -3.35160300 -1.19282300
C 3.62015900 -3.36731300 1.19955500
C 3.86015300 -4.04489300 0.00338100
H 3.85715800 -3.85773200 -2.13556400
H 3.76888400 -3.88546800 2.14256600
C -3.00357200 -1.35708600 -0.00314200
C -3.24602700 -2.02085100 1.21659900
C -3.19442300 -2.03702600 -1.22308200
C -3.67016800 -3.35160400 1.19282300
C -3.62016200 -3.36731200 -1.19955600
C -3.86015300 -4.04489300 -0.00338200
H -3.85715300 -3.85773300 2.13556300
H -3.76888800 -3.88546600 -2.14256700
C 3.05563800 -1.31302400 -2.53833500
C 2.95083100 -1.34606400 2.54500000
C 4.28513100 -5.49380200 0.00267900
C -2.95083600 -1.34606200 -2.54500100
C -3.05563300 -1.31302600 2.53833500
C -4.28513100 -5.49380200 -0.00268000
H -4.85820600 -5.74207400 -0.89919600
H -3.41376400 -6.15757100 0.02269800
H -4.89974000 -5.72907400 0.86952100
H 3.41376400 -6.15757100 -0.02270200
H 4.89974200 -5.72907300 -0.86952000
H 4.85820400 -5.74207500 0.89919700
H -3.28618500 -1.97891400 3.37205300
H -2.02680800 -0.96206500 2.65965300
H -3.70038600 -0.43307000 2.61871100
H -3.57481000 -0.45412000 -2.65204400
H -1.91190100 -1.01723300 -2.63943800
H -3.17019500 -2.01532400 -3.37903400
H 1.91189600 -1.01723600 2.63943700
H 3.17018900 -2.01532600 3.37903400
H 3.57480300 -0.45412100 2.65204500
H 2.02681400 -0.96206200 -2.65965400
H 3.70039200 -0.43306900 -2.61871000
H 3.28619100 -1.97891200 -3.37205300

IFS-T

C 1.20299100 -0.48847300 0.00552200
C 1.19498500 -1.93773800 0.00392200
C 0.00000000 -2.65240200 0.00000100
C -1.19498400 -1.93773800 -0.00392000
C -1.20299100 -0.48847400 -0.00552200
C 0.00000000 0.23289800 0.00000000
H 0.00000000 -3.73763200 0.00000100
H 0.00000000 1.31761200 -0.00000200
C 2.58348400 -2.37962100 0.00710700
C 2.55351100 -0.03960700 0.00810000
C 3.40351100 -1.21526600 0.00913600
C 3.17385100 -3.63712600 0.01145300
C 4.79524800 -1.32058500 0.01863500
C 4.56577900 -3.74312900 0.01861800
C 5.37938500 -2.59582400 0.02280800
S 7.13660600 -2.88403900 0.03338700
C 7.85334400 -1.21074900 0.05095200
H 8.93318400 -1.36167700 0.06170700
H 7.56460300 -0.66037500 0.94705000
H 7.58425400 -0.64933900 -0.84442300
H 2.57172300 -4.53957700 0.01049500
H 5.02804900 -4.72389700 0.02247600

H 5.39908200 -0.42250100 0.02388300
C -2.58348400 -2.37962100 -0.00710600
C -2.55351100 -0.03960700 -0.00810100
C -3.40351100 -1.21526700 -0.00913700
C -3.17385100 -3.63712600 -0.01144900
C -4.56577900 -3.74313000 -0.01861400
C -4.79524800 -1.32058500 -0.01863500
H -5.39908200 -0.42250100 -0.02388300
C -5.37938500 -2.59582400 -0.02280500
H -2.57172300 -4.53957700 -0.01049000
H -5.02804900 -4.72389700 -0.02247000
S -7.13660600 -2.88404000 -0.03338300
C -7.85334300 -1.21074900 -0.05094300
H -8.93318400 -1.36167700 -0.06169600
H -7.58425200 -0.64934100 0.84443200
H -7.56460500 -0.66037400 -0.94704200
C -3.02106600 1.36684900 -0.01067100
C -3.15063100 2.06652000 -1.22754300
C -3.34869200 2.00442400 1.20316600
C -3.60284000 3.38806200 -1.20718600
C -3.79593600 3.32755700 1.17732500
C -3.92760300 4.03895400 -0.01613600
H -3.70556800 3.92048400 -2.14842500
H -4.04651700 3.81366400 2.11588700
C 3.02106600 1.36685000 0.01066900
C 3.15060300 2.06653100 1.22753800
C 3.34872000 2.00441400 -1.20316700
C 3.60281200 3.38807300 1.20717900
C 3.79596300 3.32754800 -1.17732700
C 3.92760200 4.03895500 0.01613100
H 3.70551900 3.92050400 2.14841600
H 4.04656600 3.81364700 -2.11588700
C -2.81587000 1.40374700 -2.54390300
C -3.21495500 1.27849000 2.52201300
C -4.37975700 5.47966600 -0.01624000
C 3.21501200 1.27846900 -2.52201100
C 2.81581400 1.40376900 2.54389600
C 4.37975600 5.47966700 0.01623300
H 5.04597200 5.68907600 -0.82410700
H 3.52408000 6.15887200 -0.06773900
H 4.90718200 5.73239100 0.93918400
H -3.52407900 6.15887200 0.06771100
H -4.90719900 5.73238300 -0.93918300
H -5.04595600 5.68908100 0.82411100
H 3.02288400 2.07435700 3.38009100
H 1.76056000 1.11979400 2.59142300
H 3.39636400 0.48838000 2.69063800
H 3.85378200 0.39121300 -2.55992800
H 2.18918300 0.93592300 -2.68558600
H 3.49133200 1.92945000 -3.35367100
H -2.18912300 0.93594900 2.68557200
H -3.49126200 1.92947600 3.35367300
H -3.85372200 0.39123200 2.55995000
H -1.76061800 1.11976900 -2.59144800
H -3.39642500 0.48835900 -2.69062700
H -3.02295500 2.07432900 -3.38009900

IFSN CS

C -1.17848900 -0.57916000 -0.00010900
C -1.19813300 -2.00603400 -0.00002700
C 0.00000000 -2.71966300 0.00001200
C 1.19813300 -2.00603400 -0.00002700
C 1.17848900 -0.57916000 -0.00010900
C 0.00000000 0.16462500 -0.00014500
H 0.00000000 -3.80422700 0.00007600
H 0.00000000 1.24749000 -0.00019300
C 2.59294300 -2.39726700 0.00000000
C -3.35624500 -1.20692000 -0.00006700
C -3.26639800 -3.62239900 0.00006900
C -4.75171600 -1.20960500 -0.00006900
C -4.65390900 -3.64458000 0.00006800
C -5.39953300 -2.44658500 0.00000000
S -7.17355300 -2.63770200 0.00003100
C -7.79203000 -0.92644100 -0.00029900
H -8.87912600 -1.01190900 -0.00034700
H -7.48054100 -0.38720300 -0.89599100
H -7.48064800 -0.38689100 0.89524200
H -2.71523000 -4.55649600 0.00011700
H -5.17611500 -4.59462900 0.00011500
H -5.29628300 -0.27526700 -0.00012500
C 2.59294300 -2.39726700 0.00000000
C 3.35624500 -1.20692000 -0.00006700
C 3.26639800 -3.62239900 0.00006900
C 4.65390900 -3.64458000 0.00006800
C 4.75171600 -1.20960500 -0.00006900
H 5.29628300 -0.27526700 -0.00012500
C 5.39953300 -2.44658500 0.00000000
H 2.71523000 -4.55649600 0.00011700
H 5.17611500 -4.59462900 0.00011500
S 7.17355300 -2.63770200 0.00003100
C 7.79203000 -0.92644100 -0.00029900
H 8.87912600 -1.01190900 -0.00034700
H 7.48054100 -0.38720300 -0.89599000
H 7.48064800 -0.38689100 0.89524200
C 2.88954400 1.26357400 -0.00003600
C 3.08303100 1.92227900 1.22444000
C 3.08253200 1.92261500 -1.22441100
C 3.47396900 3.26309900 1.19946600
C 3.47347700 3.26342700 -1.19922900
C 3.67098000 3.95107300 0.00017400
H 3.62913400 3.78061200 2.14139900
H 3.62826100 3.78119900 -2.14108200
C -2.88954400 1.26357400 -0.00003600
C -3.08253200 1.92261500 -1.22441100
C -3.08303100 1.92227900 1.22444000
C -3.47347700 3.26342700 -1.19922900
C -3.47396900 3.26309900 1.19946600
C -3.67098000 3.95107300 0.00017400
H -3.62826000 3.78119900 -2.14108200
H -3.62913500 3.78061200 2.14139900
C 2.87953700 1.19996400 2.53354700
C 2.87849900 1.20065600 -2.53363200
C 4.06004200 5.40997300 0.00029900
C -2.87953700 1.19996400 2.53354700

C	-2.87849900	1.20065600	-2.53363200
C	-4.06004200	5.40997300	0.00029900
H	-4.64768300	5.66621800	0.88482600
H	-3.17142600	6.05076800	0.00068300
H	-4.64713600	5.66651500	-0.88450600
H	3.17142600	6.05076800	0.00068400
H	4.64768400	5.66621700	0.88482600
H	4.64713500	5.66651500	-0.88450600
H	-3.06142500	1.86969600	-3.37614100
H	-1.85966400	0.81318000	-2.61964900
H	-3.55189500	0.34389700	-2.62574100
H	-3.55307900	0.34327300	2.62521400
H	-1.86078500	0.81232600	2.61980200
H	-3.06265600	1.86881400	3.37616600
H	1.85966500	0.81317900	-2.61964900
H	3.06142500	1.86969700	-3.37614100
H	3.55189600	0.34389800	-2.62574100
H	1.86078500	0.81232600	2.61980200
H	3.55307900	0.34327300	2.62521400
H	3.06265500	1.86881500	3.37616600
N	-2.49306600	-0.11191500	-0.00016100
N	2.49306600	-0.11191500	-0.00016100

IFSN OS

C	-0.57916000	0.00010900	1.17848900
C	-2.00603400	0.00002700	1.19813300
C	-2.71966300	-0.00001200	0.00000000
C	-2.00603400	0.00002700	-1.19813300
C	-0.57916000	0.00010900	-1.17848900
C	0.16462500	0.00014500	0.00000000
H	-3.80422700	-0.00007600	0.00000000
H	1.24749000	0.00019300	0.00000000
C	-2.39726700	0.00000000	2.59294300
C	-1.20692000	0.00006700	3.35624500
C	-3.62239900	-0.00006900	3.26639800
C	-1.20960500	0.00006900	4.75171600
C	-3.64458000	-0.00006800	4.65390900
C	-2.44658500	0.00000000	5.39953300
S	-2.63770200	-0.00003100	7.17355300
C	-0.92644100	0.00029900	7.79203000
H	-1.01190900	0.00034700	8.87912600
H	-0.38720300	0.89599100	7.48054100
H	-0.38689100	-0.89524200	7.48064800
H	-4.55649600	-0.00011700	2.71523000
H	-4.59462900	-0.00011500	5.17611500
H	-0.27526700	0.00012500	5.29628300
C	-2.39726700	0.00000000	-2.59294300
C	-1.20692000	0.00006700	-3.35624500
C	-3.62239900	-0.00006900	-3.26639800
C	-3.64458000	-0.00006800	-4.65390900
C	-1.20960500	0.00006900	-4.75171600
H	-0.27526700	0.00012500	-5.29628300
C	-2.44658500	0.00000000	-5.39953300
H	-4.55649600	-0.00011700	-2.71523000
H	-4.59462900	-0.00011500	-5.17611500
S	-2.63770200	-0.00003100	-7.17355300
C	-0.92644100	0.00029900	-7.79203000
H	-1.01190900	0.00034700	-8.87912600
H	-0.38720300	0.89599100	-7.48054100
H	-0.38689100	-0.89524200	-7.48064800
C	1.26357400	0.00003600	-2.88954400
C	1.92227900	-1.22444000	-3.08303100
C	1.92261500	1.22441100	-3.08253200
C	3.26309900	-1.19946600	-3.47396900
C	3.26342700	1.19922900	-3.47347700
C	3.95107300	-0.00017400	-3.67098000
H	3.78061200	-2.14139900	-3.62913400
H	3.78119900	2.14108200	-3.62826100
C	1.26357400	0.00003600	2.88954400
C	1.92261500	1.22441100	3.08253200
C	1.92227900	-1.22444000	3.08303100
C	3.26342700	1.19922900	3.47347700
C	3.26309900	-1.19946600	3.47396900
C	3.95107300	-0.00017400	3.67098000
H	3.78119900	2.14108200	3.62826100
H	3.78061200	-2.14139900	3.62913400
C	1.19996400	-2.53354700	-2.87953700
C	1.20065600	2.53363200	-2.87849900
C	5.40997300	-0.00029900	-4.06004200
C	1.19996400	-2.53354700	2.87953700
C	1.20065600	2.53363200	2.87849900
C	5.40997300	-0.00029900	4.06004200
H	5.66621800	-0.88482600	4.64768300
H	6.05076800	-0.00068300	3.17142600
H	5.66651500	0.88450600	4.64713600
H	6.05076800	-0.00068300	-3.17142600
H	5.66621800	-0.88482600	-4.64768300
H	5.66651500	0.88450600	-4.64713600
H	1.86969600	3.37614100	3.06142500
H	0.81318000	2.61964900	1.85966400
H	0.34389700	2.62574100	3.55189500
H	0.34327300	-2.62521400	3.55307900
H	0.81232600	-2.61980200	1.86078500
H	1.86881400	-3.37616600	3.06265600
H	0.81318000	2.61964900	-1.85966400
H	1.86969600	3.37614100	-3.06142500
H	0.34389700	2.62574100	-3.55189500
H	0.81232600	-2.61980200	-1.86078500
H	0.34327300	-2.62521400	-3.55307900
H	1.86881400	-3.37616600	-3.06265600
N	-0.11191500	0.00016100	2.49306600
N	-0.11191500	0.00016100	-2.49306600

IFSN T

C	1.18464000	-0.56998800	0.00002700
C	1.22240800	-2.00775900	0.00010100
C	-0.01400300	-2.77028400	0.00014100
C	-1.21552100	-2.03124200	0.00010300
C	-1.18799800	-0.62091500	0.00003000
C	0.01937000	0.16378000	-0.00001000
H	-0.00079000	-3.85147700	0.00019700
H	0.00148300	1.24521300	-0.00006600
C	2.55948300	-2.38304900	0.00012000
C	3.36842800	-1.17490600	0.00005800

C	3.23918900	-3.64739200	0.00018400
C	4.74547400	-1.19418900	0.00005800
C	4.61008200	-3.66441000	0.00018500
C	5.38207400	-2.46145400	0.00012300
S	7.13203300	-2.68973500	0.00012700
C	7.80565200	-0.99924600	0.00007500
H	8.88930500	-1.11867000	0.00008500
H	7.50829500	-0.45289700	0.89568800
H	7.50830600	-0.45295500	-0.89557800
H	2.67641700	-4.57258800	0.00023300
H	5.13399000	-4.61447200	0.00023400
H	5.30698100	-0.27035600	0.00001000
C	-2.60890400	-2.40596900	0.00012300
C	-3.36913000	-1.20895000	0.00006100
C	-3.30142900	-3.62481500	0.00018500
C	-4.68889500	-3.62325700	0.00018600
C	-4.76172900	-1.18882600	0.00006100
H	-5.29288000	-0.24653100	0.00001300
C	-5.42618000	-2.41792000	0.00012400
H	-2.76454000	-4.56678000	0.00023400
H	-5.22313200	-4.56710500	0.00023500
S	-7.20387200	-2.59208400	0.00013400
C	-7.80108900	-0.87370000	0.00005000
H	-8.88924800	-0.94455600	0.00005500
H	-7.48274200	-0.33770600	0.89552500
H	-7.48274500	-0.33779400	-0.89547800
C	-2.85729700	1.25250800	-0.00006300
C	-3.04314700	1.91007400	-1.22562800
C	-3.04314700	1.91019600	1.22543600
C	-3.42167700	3.25439400	-1.19955300
C	-3.42167700	3.25451400	1.19922700
C	-3.61300100	3.94375000	-0.00019700
H	-3.57211600	3.77315900	-2.14145400
H	-3.57211600	3.77337200	2.14107700
C	2.89169100	1.28256900	-0.00006800
C	3.07929300	1.94130100	1.22472900
C	3.07928900	1.94117700	-1.22493200
C	3.46145900	3.28461400	1.19935000
C	3.46145600	3.28449200	-1.19969100
C	3.65433000	3.97342200	-0.00020600
H	3.61240500	3.80346300	2.14120700
H	3.61239900	3.80324600	-2.14160000
C	-2.83959000	1.18791000	-2.53466600
C	-2.83959000	1.18816300	2.53454700
C	-3.99039600	5.40563600	-0.00027000
C	2.87701900	1.21692600	-2.53295200
C	2.87702600	1.21718400	2.53282300
C	4.03367300	5.43488800	-0.00028000
H	4.61926600	5.69519200	-0.88497800
H	3.14077200	6.06968100	-0.00031000
H	4.61927000	5.69528100	0.88438900
H	-3.09659200	6.03913000	-0.00030200
H	-4.57567400	5.66658200	-0.88494400
H	-4.57567200	5.66667100	0.88438000
H	3.06291500	1.88410800	3.37636000
H	1.85709400	0.83220900	2.61910500
H	3.54905800	0.35883200	2.62079300
H	3.54905000	0.35856500	-2.62083600
H	1.85708700	0.83194400	-2.61919300
H	3.06290800	1.88376400	-3.37655800
H	-1.80843100	0.83865100	2.63848300
H	-3.06211200	1.84600900	3.37620400
H	-3.48200400	0.30665900	2.60915000
H	-1.80843000	0.83839200	-2.63857000
H	-3.48200100	0.30639700	-2.60918000
H	-3.06211600	1.84567200	-3.37638900
N	2.50462100	-0.09641000	0.00000200
N	-2.48034200	-0.13138600	0.00000600

DH-IFS-anti isomer

C	1.17573800	-0.29422200	0.19909000
C	1.18068400	-1.70582800	0.19317300
C	0.00000000	-2.42512400	-0.00000100
C	-1.18068500	-1.70582800	-0.19317400
C	-1.17573900	-0.29422200	-0.19909100
C	0.00000000	0.42195000	-0.00000100
H	0.00000000	-3.51005500	-0.00000100
H	-0.00000100	1.50741400	-0.00000100
C	2.55803400	-2.16507500	0.39646000
C	2.57387400	0.23673500	0.49964500
C	3.39717600	-1.04682400	0.52677000
C	3.10743600	-3.44826500	0.45083000
C	4.76781500	-1.18756500	0.70072000
C	4.47797700	-3.59685800	0.62485800
C	5.31810400	-2.47550000	0.74719000
S	7.05625000	-2.81552500	0.95193500
C	7.81077100	-1.16210700	1.05110400
H	8.88028000	-1.34052800	1.16643700
H	7.45019700	-0.60776400	1.91852200
H	7.64446900	-0.59017700	0.13743800
H	2.48186100	-4.32906800	0.35396100
H	4.90928200	-4.59135700	0.66185200
H	5.39181600	-0.30689200	0.78413200
C	-2.55803500	-2.16507500	-0.39646200
C	-2.57387500	0.23673600	-0.49964700
C	-3.39717700	-1.04682400	-0.52677100
C	-3.10743700	-3.44826500	-0.45083000
C	-4.47797900	-3.59685700	-0.62485800
C	-4.76781600	-1.18756400	-0.70072000
H	-5.39181700	-0.30689100	-0.78413100
C	-5.31810500	-2.47549900	-0.74718900
H	-2.48186200	-4.32906800	-0.35396200
H	-4.90928300	-4.59135700	-0.66185100
S	-7.05625200	-2.81552300	-0.95193300
C	-7.81077200	-1.16210500	-1.05110000
H	-8.88028100	-1.34052600	-1.16643200
H	-7.64447000	-0.59017600	-0.13743500
H	-7.45019900	-0.60776300	-1.91851800
C	-3.09002000	1.39245600	0.35243100
C	-3.39607000	2.62504400	-0.26195200
C	-3.27042300	1.25775900	1.74766000
C	-3.87086900	3.68639200	0.51640900
C	-3.74640600	2.34494700	2.48381100
C	-4.05191700	3.57067100	1.89217500

H -4.10454800 4.62825600 0.02882700
H -3.88287800 2.22581000 3.55491100
C 3.09002000 1.39245500 -0.35243200
C 3.39606800 2.62504300 0.26195100
C 3.27042500 1.25775800 -1.74766100
C 3.87086900 3.68639100 -0.51640900
C 3.74641100 2.34494600 -2.48381100
C 4.05192200 3.57067000 -1.89217500
H 4.10454800 4.62825500 -0.02882700
H 3.88288600 2.22580800 -3.55491000
C -3.23095700 2.85575600 -1.75122700
C -2.97052700 -0.02831000 2.48246000
C -4.53460200 4.73652100 2.72113600
C 2.97052900 -0.02831000 -2.48246200
C 3.23095100 2.85575500 1.75122500
C 4.53461000 4.73651800 -2.72113500
H 5.20309400 4.40763800 -3.52101800
H 3.69444000 5.25773700 -3.19324900
H 5.07081500 5.46577700 -2.10959800
H -3.69442900 5.25774400 3.19324100
H -5.07081500 5.46577600 2.10960300
H -5.20307700 4.40764000 3.52102700
H 3.52249400 3.87512200 2.01007500
H 2.19525500 2.72025100 2.07783100
H 3.85060300 2.17833500 2.34724700
H 3.59858600 -0.85071700 -2.13091800
H 1.93195800 -0.33928900 -2.34643500
H 3.14746700 0.09500700 -3.55251600
H -1.93195900 -0.33929600 2.34642400
H -3.14745500 0.09500900 3.55251500
H -3.59859300 -0.85071300 2.13092300
H -2.19525900 2.72025700 -2.07783400
H -3.85060500 2.17833100 -2.34724700
H -3.52250600 3.87512000 -2.01007700
H 2.54229600 0.58672900 1.53624800
H -2.54229600 0.58673000 -1.53625000

DH-IFS-syn isomer

C 1.19281700 -0.51022900 0.65141100
C 1.19635100 -1.85549700 0.22337300
C 0.00000100 -2.54146900 0.00734500
C -1.19634800 -1.85549800 0.22337300
C -1.19281600 -0.51023100 0.65141300
C 0.00000000 0.17233300 0.86814000
H 0.00000200 -3.57410000 -0.32539900
H 0.00000000 1.21253100 1.17858400
C 2.58805000 -2.28654200 0.06000900
C 2.62228800 -0.02409100 0.87216800
C 3.43783400 -1.21746400 0.38578200
C 3.13814100 -3.50070700 -0.35785400
C 4.81840300 -1.33712600 0.29419100
C 4.51862500 -3.62794400 -0.45183000
C 5.36816700 -2.55335700 -0.13272700
S 7.11616400 -2.85428700 -0.31341200
C 7.87893000 -1.27483900 0.17294900
H 8.95303200 -1.42935700 0.06608100
H 7.65992000 -1.02699600 1.21234200
H 7.57192600 -0.46133900 -0.48545900
H 2.50464200 -4.34311600 -0.61392000
H 4.94956600 -4.56741400 -0.78063100
H 5.44806500 -0.49114900 0.53859900
C -2.58804700 -2.28654400 0.06000700
C -2.62228700 -0.02409600 0.87217300
C -3.43783200 -1.21746700 0.38578200
C -3.13813700 -3.50070700 -0.35786000
C -4.51862100 -3.62794500 -0.45183900
C -4.81840000 -1.33712900 0.29418800
H -5.44806400 -0.49115300 0.53859700
C -5.36816400 -2.55335800 -0.13273500
H -2.50463700 -4.34311500 -0.61392800
H -4.94956100 -4.56741400 -0.78064400
S -7.11616000 -2.85428900 -0.31342400
C -7.87892800 -1.27484100 0.17293700
H -8.95302900 -1.42935900 0.06606500
H -7.65992100 -1.02700000 1.21233100
H -7.57192100 -0.46134000 -0.48547000
C -2.99496800 1.35477500 0.33579300
C -2.95538700 1.64453900 -1.04672000
C -3.39132500 2.36590300 1.23627400
C -3.30748400 2.92174900 -1.48826800
C -3.73586000 3.63079100 0.74756800
C -3.69896000 3.93313400 -0.61116000
H -3.27355400 3.12946400 -2.55396500
H -4.04091000 4.39829700 1.45280200
C 2.99496600 1.35477900 0.33578300
C 3.39132400 2.36590900 1.23626100
C 2.95538200 1.64453900 -1.04673000
C 3.73585600 3.63079700 0.74755000
C 3.30747700 2.92174800 -1.48828300
C 3.69895400 3.93313600 -0.61117800
H 4.04090700 4.39830500 1.45278200
H 3.27354500 3.12946100 -2.55398000
C -2.54925100 0.61715900 -2.07771600
C -3.46341600 2.14028000 2.73377000
C -4.04149400 5.31348400 -1.11759600
C 2.54924600 0.61715700 -2.07772500
C 3.46341800 2.14029000 2.73375700
C 4.04148700 5.31348500 -1.11761900
H 4.57533300 5.26784400 -2.07038700
H 3.13565900 5.90793400 -1.28042700
H 4.66623800 5.85629100 -0.40459700
H -3.13566700 5.90793900 -1.28038800
H -4.57532700 5.26784600 -2.07037100
H -4.66625700 5.85628300 -0.40457900
H 3.78646900 3.05266000 3.23809100
H 2.49387200 1.85982600 3.15696200
H 4.17388300 1.35149400 3.00066300
H 3.24151700 -0.22839800 -2.09972400
H 1.55563500 0.21108100 -1.87471300
H 2.53317700 1.06358200 -3.07366700
H -2.49386900 1.85981000 3.15697200
H -3.78646200 3.05264900 3.23810800
H -4.17388300 1.35148500 3.00067600
H -1.55564500 0.21107300 -1.87469800

H	-3.24152900	-0.22839100	-2.09972500
H	-2.53317100	1.06358800	-3.07365600
H	-2.76162600	0.00198900	1.95755600
H	2.76162600	0.00199800	1.95755100

10.3.2. Single Molecule Junctions

IFA-CS

Au	20.177234	9.984133	14.138018
Au	23.075862	9.803585	14.134212
Au	26.016060	9.951271	14.109525
Au	21.463792	12.596327	14.136519
Au	24.645911	12.557462	14.129734
Au	23.072092	15.011493	14.146246
Au	21.603788	10.818904	16.338620
Au	24.626950	10.742528	16.297705
Au	23.055561	13.341597	16.360855
Au	23.168664	11.624656	18.494130
C	20.876625	9.386768	25.824496
C	20.629004	10.747041	25.317546
C	19.800412	11.632321	25.993134
C	19.201216	11.187484	27.224837
C	19.453646	9.832435	27.746257
C	20.266764	8.938341	27.054996
H	19.601489	12.657399	25.622274
H	20.461463	7.911577	27.425974
C	18.354738	11.887941	28.111415
C	18.051043	10.990751	29.232820
C	17.253873	11.213832	30.381645
C	17.138229	10.161733	31.326317
C	17.816597	8.923183	31.131066
C	18.601533	8.702134	29.976500
C	18.719950	9.729709	29.018352
H	16.742683	12.185687	30.518174
H	17.726116	8.130828	31.901653
H	19.114801	7.730040	29.842306
S	16.070021	10.228441	32.765748
C	15.575190	11.980659	32.892189
H	16.455923	12.658876	32.876612
H	15.076138	12.061757	33.884291
H	14.853848	12.241140	32.087228
C	21.413720	10.894402	24.086914
C	21.764214	8.716768	24.949326
C	22.110136	9.651099	23.865700
C	21.571333	11.971452	23.194359
C	22.982522	9.499608	22.759891
C	22.432386	11.820248	22.085189
C	23.140937	10.602169	21.878573
S	24.329154	10.629499	20.534663
C	24.469580	8.886476	19.998539
H	24.972276	8.932171	19.005050
H	23.465122	8.426337	19.877834
H	25.097529	8.301516	20.706187
H	21.030954	12.926396	23.347791
H	22.558628	12.645377	21.355344
H	23.528693	8.548950	22.607755
C	17.871045	13.289306	27.944300
C	16.661775	13.545012	27.231485
C	18.631265	14.371651	28.479491
C	16.229225	14.883054	27.073359
C	18.160841	15.694287	28.299912
C	16.963042	15.973067	27.599411
H	15.288060	15.079100	26.520669
H	18.752479	16.532874	28.719826
C	19.931184	14.107127	29.212523
C	15.862071	12.399269	26.644174
C	16.495199	17.399899	27.392771
H	16.439526	11.871428	25.848933
H	15.613452	11.635252	27.418181
H	14.911313	12.761137	26.193912
H	20.361047	15.048226	29.621152
H	19.787790	13.392691	30.057457
H	20.688928	13.649737	28.533309
H	16.911464	17.823550	26.446878
H	15.385599	17.461063	27.320195
H	16.824546	18.063854	28.224024
C	22.269872	7.318892	25.096838
C	21.523370	6.230855	24.551664
C	23.491953	7.068411	25.790980
C	22.016734	4.913066	24.705877
C	23.949291	5.734434	25.920598
C	23.226217	4.640706	25.387786
H	21.434928	4.071920	24.277198
H	24.901419	5.546365	26.456623
C	20.224358	6.483575	23.813290
C	24.282615	8.214639	26.389224
C	23.717165	3.217289	25.563566
H	24.827259	3.169924	25.635691
H	23.306110	2.764829	26.497974
H	23.398613	2.567565	24.717144
H	24.539440	8.981284	25.620071
H	23.694575	8.738618	27.179265
H	25.229134	7.852562	26.848379
H	20.368205	7.198710	22.968877
H	19.807657	5.538372	23.400445
H	19.458111	6.934489	24.486759
Au	17.362383	10.018808	34.926029
Au	17.341325	8.274027	37.095557
Au	15.813065	10.868764	37.107256
Au	18.815817	10.807487	37.086049
Au	17.301574	6.617656	39.287971
Au	15.725288	9.066509	39.291115
Au	18.931874	9.053831	39.299904
Au	14.397140	11.671654	39.302448
Au	17.321086	11.823988	39.291839
Au	20.225051	11.672932	39.288002

IFS-OS

Au	20.147932	9.975067	14.136567
Au	23.056620	9.820637	14.129805
Au	25.975302	9.983222	14.137087
Au	21.468769	12.574423	14.134320
Au	24.658427	12.571142	14.144393
Au	23.081537	15.000214	14.141862
Au	21.565867	10.800899	16.349251
Au	24.531821	10.775938	16.338459

Au	23.048136	13.358987	16.353799
Au	22.961968	11.579799	18.538835
C	18.962386	12.824354	27.835923
C	19.417402	11.434770	27.843211
C	20.286155	10.958385	26.834606
C	20.700006	11.850998	25.818661
C	20.247824	13.241212	25.814222
C	19.386961	13.732166	26.829738
H	20.630627	9.904058	26.835822
H	19.054795	14.791560	26.837852
C	18.802473	10.800167	28.993906
C	18.085734	13.050290	28.961452
C	17.985293	11.803541	29.671335
C	18.837987	9.478051	29.513406
C	17.221395	11.477620	30.829234
C	18.073116	9.160973	30.648837
C	17.267614	10.153987	31.305961
S	16.343196	9.546322	32.720638
C	14.972988	10.739697	32.894339
H	14.377541	10.377664	33.761913
H	14.354523	10.735207	31.970110
H	15.343513	11.760582	33.125958
H	19.442540	8.687413	29.028121
H	18.070454	8.125774	31.041131
H	16.601151	12.260136	31.308719
C	21.541531	11.685569	24.648995
C	20.795179	13.925207	24.663630
C	21.592386	12.965989	23.946067
C	22.251794	10.577002	24.117654
C	22.994591	10.734747	22.935971
C	22.327556	13.109549	22.730068
H	22.326432	14.086522	22.207572
C	23.030894	11.992393	22.239024
H	22.237708	9.593724	24.627219
H	23.572751	9.882479	22.532595
S	24.067527	11.993488	20.773329
C	24.435589	13.758799	20.507751
H	25.125309	13.784214	19.634836
H	23.521847	14.335724	20.250205
H	24.936676	14.175076	21.409436
C	20.512265	15.326273	24.241859
C	21.421257	16.385114	24.548672
C	19.318564	15.597219	23.505388
C	21.112148	17.697955	24.116632
C	19.052943	16.923244	23.092751
C	19.934453	17.990148	23.387170
H	21.818593	18.517907	24.355497
H	18.130714	17.126213	22.512350
C	17.405359	14.317276	29.338494
C	16.236320	14.746536	28.637781
C	17.910130	15.089180	30.430011
C	15.597436	15.943306	29.040821
C	17.237765	16.278322	30.797985
C	16.077937	16.723269	30.119891
H	14.684335	16.268832	28.501807
H	17.634075	16.874605	31.644971
C	22.689043	16.109860	25.334181
C	18.364658	14.471970	23.156377
C	19.611728	19.408839	22.962837
C	19.165663	14.656211	31.162114
C	15.665224	13.916789	27.503571
C	15.393610	18.018463	30.509595
H	15.534525	18.248512	31.589904
H	15.809658	18.879173	29.930954
H	14.300167	17.981262	30.302678
H	19.027635	19.939315	23.753842
H	20.535377	20.003392	22.781734
H	19.000157	19.429134	22.032911
H	14.704610	14.345247	27.138792
H	16.367077	13.861006	26.637763
H	15.478458	12.865063	27.826302
H	19.020368	13.687720	31.696385
H	20.011479	14.501700	30.451621
H	19.475149	15.418292	31.910983
H	17.881683	14.051380	24.070014
H	17.560587	14.823652	22.473507
H	18.898920	13.626061	22.664090
H	22.455845	15.719000	26.352835
H	23.321752	15.337763	24.834926
H	23.296978	17.035065	25.449420
Au	17.461289	10.026413	34.910944
Au	17.360443	8.286781	37.104046
Au	15.856225	10.874548	37.076551
Au	18.835861	10.851910	37.080908
Au	17.317020	6.636022	39.295273
Au	15.734817	9.081424	39.283293
Au	18.932002	9.080022	39.298997
Au	14.421246	11.669042	39.286665
Au	17.334031	11.844664	39.287163
Au	20.243375	11.685878	39.292565

IFS-T

Au	20.151944	9.970463	14.129066
Au	23.058961	9.811329	14.131355
Au	25.977151	9.979957	14.132250
Au	21.464284	12.573308	14.133858
Au	24.659569	12.570553	14.141261
Au	23.083105	15.000554	14.138598
Au	21.567406	10.799780	16.344506
Au	24.532970	10.777226	16.329531
Au	23.051828	13.357907	16.352669
Au	22.957155	11.566216	18.538017
C	18.963647	12.830042	27.833696
C	19.420520	11.440993	27.841280
C	20.288993	10.963060	26.831894
C	20.697023	11.852208	25.811459
C	20.241581	13.241227	25.804366
C	19.384369	13.733428	26.824979
H	20.636168	9.910463	26.837462
H	19.054768	14.792518	26.831651
C	18.807803	10.804914	28.992898
C	18.087195	13.054391	28.961656
C	17.987887	11.804736	29.670146
C	18.852757	9.484422	29.514038

C	17.226480	11.474679	30.828235
C	18.100562	9.165684	30.657501
C	17.287089	10.153325	31.310825
S	16.359363	9.540808	32.717447
C	14.994833	10.737840	32.898967
H	14.405833	10.371303	33.769346
H	14.374233	10.735944	31.976023
H	15.367406	11.758589	33.130868
H	19.456315	8.695438	29.024526
H	18.112264	8.134037	31.059784
H	16.594990	12.248599	31.306567
C	21.537545	11.682686	24.639801
C	20.785870	13.921745	24.651933
C	21.586035	12.961059	23.934268
C	22.242543	10.572553	24.106647
C	22.976577	10.724409	22.918787
C	22.320294	13.103501	22.719267
H	22.325816	14.078487	22.195430
C	23.017563	11.982611	22.226002
H	22.231676	9.590638	24.617830
H	23.550657	9.870553	22.508452
S	24.061549	11.990044	20.765451
C	24.418934	13.757556	20.497110
H	25.104150	13.783199	19.620695
H	23.500918	14.330226	20.244008
H	24.919359	14.177049	21.397970
C	20.502221	15.322545	24.229758
C	21.415294	16.375940	24.538829
C	19.308580	15.600369	23.495610
C	21.113318	17.691475	24.111870
C	19.048220	16.929752	23.087346
C	19.935641	17.991261	23.386263
H	21.823720	18.508427	24.351907
H	18.127640	17.139723	22.506498
C	17.409850	14.323137	29.342068
C	16.239833	14.749266	28.641482
C	17.914389	15.097412	30.432449
C	15.597258	15.943963	29.044130
C	17.239178	16.285530	30.799700
C	16.078880	16.727611	30.119977
H	14.682706	16.266757	28.506155
H	17.634396	16.884997	31.644642
C	22.681228	16.090847	25.323898
C	18.345978	14.482320	23.144373
C	19.617697	19.412888	22.965193
C	19.168767	14.665570	31.167953
C	15.670155	13.914551	27.510225
C	15.391443	18.021247	30.509958
H	15.531493	18.250695	31.590667
H	15.806538	18.881873	29.931964
H	14.297665	17.981468	30.303183
H	19.033902	19.941702	23.757131
H	20.544107	20.005162	22.786971
H	19.007145	19.437169	22.033831
H	14.709456	14.339623	27.143088
H	16.371635	13.857356	26.644403
H	15.485974	12.863945	27.838044
H	19.022725	13.697553	31.703213
H	20.017102	14.511015	30.460290
H	19.476240	15.428516	31.917255
H	17.861011	14.060236	24.056926
H	17.542394	14.842318	22.464271
H	18.873564	13.635075	22.646314
H	22.445820	15.694223	26.339967
H	23.311791	15.320839	24.819517
H	23.292419	17.012739	25.448686
Au	17.470590	10.012885	34.914289
Au	17.356930	8.273245	37.109056
Au	15.856211	10.867911	37.083415
Au	18.833352	10.841000	37.086130
Au	17.314465	6.627413	39.298318
Au	15.738038	9.079917	39.286126
Au	18.934108	9.075675	39.299132
Au	14.416594	11.668058	39.287904
Au	17.332097	11.840433	39.288690
Au	20.242266	11.686226	39.298287

IFSN-CS

Au	20.153119	9.966171	14.128559
Au	23.073599	9.801360	14.121069
Au	25.996188	9.981111	14.135515
Au	21.483388	12.551786	14.136606
Au	24.673189	12.564351	14.145678
Au	23.079953	14.995223	14.134286
Au	21.590280	10.742502	16.339030
Au	24.539363	10.756804	16.338244
Au	23.061382	13.359512	16.345521
Au	22.959891	11.548861	18.527572
C	18.626412	13.437348	27.638828
C	19.138004	12.091345	27.794325
C	20.035744	11.555212	26.841428
C	20.414573	12.367596	25.747346
C	19.885662	13.711906	25.632027
C	18.985534	14.280299	26.562494
H	20.423622	10.524988	26.945540
H	18.585561	15.305657	26.449773
C	18.526943	11.563339	29.007967
C	17.677215	12.598526	29.529878
C	18.609176	10.333183	29.706128
C	16.906460	12.433261	30.704506
C	17.849604	10.147026	30.875563
C	17.000349	11.184348	31.365369
S	15.993181	10.731596	32.780302
C	15.233954	12.300082	33.313247
H	14.733250	12.065403	34.280925
H	14.476895	12.633996	32.569534
H	15.996704	13.089389	33.490118
H	19.265655	9.519622	29.342196
H	17.906352	9.190350	31.429618
H	16.254612	13.252487	31.059913
C	21.278432	12.169460	24.586369
C	21.231375	13.387515	23.826073
C	22.085817	11.108220	24.104546
C	22.808841	11.269750	22.906965

C	21.940451	13.568678	22.616822
H	21.866554	14.529122	22.075233
C	22.736214	12.488833	22.165357
H	22.165294	10.156953	24.665566
H	23.451645	10.448977	22.535365
S	23.786910	12.580971	20.710912
C	23.573805	14.298799	20.138011
H	24.185294	14.371137	19.210171
H	22.515707	14.510424	19.868535
H	23.949005	15.012696	20.904288
C	20.116095	15.633377	24.017597
C	20.939751	16.707675	24.460260
C	19.025902	15.839080	23.125610
C	20.643471	18.007874	23.985354
C	18.773600	17.158465	22.680475
C	19.566570	18.255277	23.098609
H	21.276462	18.854609	24.318555
H	17.929194	17.333752	21.984109
C	17.048119	14.954929	28.888034
C	15.754197	15.114607	28.316009
C	17.661849	15.988547	29.651429
C	15.080724	16.341489	28.526183
C	16.945714	17.196679	29.832012
C	15.656755	17.395503	29.277919
H	14.071844	16.478551	28.086910
H	17.411220	18.009022	30.425330
C	22.094494	16.451520	25.407428
C	18.173003	14.669275	22.678202
C	19.253339	19.662579	22.630750
C	19.041281	15.788098	30.244049
C	15.129768	13.996561	27.506254
C	14.926118	18.711977	29.460854
H	15.206649	19.206065	30.419375
H	15.178439	19.420820	28.635338
H	13.820639	18.571853	29.451468
H	18.541287	20.159828	23.332242
H	20.170706	20.292846	22.586921
H	18.780768	19.662872	21.622105
H	14.125313	14.291507	27.130485
H	15.766911	13.730054	26.629778
H	15.016430	13.069108	28.116327
H	19.057799	14.922808	30.948252
H	19.793325	15.570994	29.448353
H	19.372341	16.693940	30.797900
H	17.702072	14.157148	23.551078
H	17.364205	15.005580	21.992958
H	18.783879	13.901628	22.146157
H	21.740983	15.994712	26.362679
H	22.832104	15.740715	24.964610
H	22.626273	17.397963	25.650700
N	17.752405	13.718831	28.694924
N	20.398207	14.303928	24.473499
Au	17.287794	10.173303	34.902400
Au	17.294566	8.383624	37.053715
Au	15.746559	10.943769	37.105634
Au	18.784950	10.880472	37.083147
Au	17.299769	6.691614	39.272086
Au	15.701834	9.096980	39.282879
Au	18.902638	9.090305	39.289209
Au	14.363346	11.694930	39.313827
Au	17.302767	11.839187	39.297045
Au	20.219753	11.679668	39.294625

DH-IFS-anti isomer

Au	20.129814	9.943662	14.121226
Au	23.064757	9.778712	14.117270
Au	26.002899	9.968674	14.127995
Au	21.471788	12.526770	14.133172
Au	24.664365	12.540789	14.141431
Au	23.076127	14.968535	14.138941
Au	21.564688	10.697384	16.325399
Au	24.536869	10.719039	16.321648
Au	23.048808	13.305584	16.336405
Au	22.931690	11.453986	18.497034
C	19.882082	13.794191	25.408265
C	20.359669	12.443729	25.464954
C	19.926747	11.563609	26.487526
C	19.004894	12.061262	27.442718
C	18.518189	13.406746	27.360088
C	18.955354	14.284002	26.348159
H	20.300701	10.523678	26.542834
H	18.587859	15.327456	26.295790
C	21.283477	12.239512	24.327118
C	20.561344	14.557098	24.272855
C	21.366773	13.457222	23.589251
C	22.016433	11.108553	23.886043
C	22.142791	13.563959	22.421480
C	22.808419	11.206343	22.723942
C	22.874582	12.426654	21.990842
S	23.976608	12.432308	20.575593
C	23.915459	14.153689	19.972934
H	24.543885	14.154306	19.052210
H	24.337454	14.850541	20.730530
H	22.881495	14.442016	19.682964
H	21.983997	10.151397	24.441591
H	23.381780	10.326215	22.370198
H	22.160400	14.516114	21.857559
C	18.410109	11.436298	28.643775
C	17.496018	13.679215	28.463533
C	17.576700	12.396960	29.291997
C	18.557735	10.147271	29.216249
C	17.895584	9.843883	30.422974
C	16.928071	12.110928	30.509026
H	16.313791	12.891754	30.996154
C	17.088817	10.818908	31.079922
H	19.178889	9.372342	28.727509
H	17.999446	8.835826	30.869558
S	16.244115	10.266675	32.566903
C	15.194891	11.687227	33.020685
H	14.703753	11.381864	33.973146
H	15.797185	12.601246	33.214848
H	14.431340	11.868645	32.232340
C	17.598542	15.008304	29.213956
C	16.527693	15.952759	29.144274

C	18.757463	15.315535	29.994871
C	16.630073	17.172315	29.859313
C	18.813339	16.544404	30.693384
C	17.761045	17.489644	30.645015
H	15.792851	17.896614	29.801845
H	19.713284	16.768683	31.301836
C	19.691534	15.436077	23.372127
C	19.939519	16.842234	23.296385
C	18.641750	14.862959	22.585843
C	19.129553	17.644690	22.454852
C	17.860554	15.704942	21.759726
C	18.082273	17.100050	21.678490
H	19.329197	18.733398	22.403432
H	17.051248	15.249344	21.154290
C	15.273020	15.706053	28.322443
C	19.929674	14.360877	30.093396
C	17.865454	18.810466	31.382711
C	18.345232	13.376628	22.608635
C	21.047073	17.520158	24.088150
C	17.204463	17.983164	20.815486
H	16.918826	17.478336	19.864951
H	16.256345	18.243402	21.344832
H	17.710859	18.940081	20.558146
H	18.461416	19.550750	30.796389
H	16.864170	19.261295	31.564127
H	18.372351	18.690378	32.367221
H	21.053795	18.614059	23.882575
H	20.922521	17.395176	25.189810
H	22.060190	17.132990	23.824404
H	19.211307	12.779333	22.237128
H	18.124831	13.015907	23.640802
H	17.468571	13.139558	21.966340
H	20.352270	14.127006	29.088010
H	20.743043	14.794246	30.716332
H	19.631873	13.387777	30.549992
H	15.496112	15.575978	27.237065
H	14.712533	14.800303	28.654871
H	14.576882	16.570399	28.410934
H	21.317924	15.221320	24.760858
H	16.494477	13.649922	27.964164
Au	17.382390	10.055975	34.874806
Au	17.328120	8.319239	37.064736
Au	15.809325	10.877101	37.071342
Au	18.800079	10.850790	37.079510
Au	17.301178	6.659060	39.272905
Au	15.715630	9.079306	39.278590
Au	18.909245	9.078974	39.293295
Au	14.398825	11.671230	39.284225
Au	17.311273	11.836237	39.287021
Au	20.216133	11.681081	39.284015

DH-IFS-syn isomer

Au	20.155715	9.974557	14.131979
Au	23.069344	9.809162	14.126103
Au	25.976674	9.998937	14.141964
Au	21.480224	12.569457	14.133903
Au	24.669076	12.581243	14.143569
Au	23.082795	15.015255	14.129282
Au	21.584821	10.772643	16.341687
Au	24.516984	10.792104	16.347094
Au	23.057274	13.390234	16.338982
Au	22.936053	11.618254	18.526077
C	19.779402	13.786038	25.717790
C	20.317476	12.457597	25.707432
C	19.965780	11.526079	26.716164
C	19.067901	11.950115	27.727896
C	18.535871	13.280691	27.721914
C	18.880907	14.207153	26.717569
H	20.381778	10.498715	26.714575
H	18.450799	15.228413	26.708123
C	21.192568	12.332652	24.521396
C	20.364936	14.613770	24.575437
C	21.182913	13.573010	23.814936
C	21.960260	11.252706	24.015629
C	21.894583	13.746265	22.613584
C	22.692774	11.421112	22.822642
C	22.661261	12.658945	22.117056
S	23.714336	12.762750	20.666450
C	23.457318	14.463656	20.058346
H	24.046853	14.519322	19.115082
H	23.840234	15.203463	20.795694
H	22.388566	14.648055	19.815024
H	22.000702	10.281735	24.546996
H	23.295200	10.581456	22.423353
H	21.841359	14.714015	22.079828
C	18.509729	11.244434	28.902569
C	17.661709	13.519863	28.950871
C	17.654937	12.143868	29.609268
C	18.680222	9.923448	29.389421
C	17.999834	9.521917	30.557381
C	16.974410	11.754626	30.776696
H	16.316117	12.476822	31.294758
C	17.146863	10.428197	31.251382
H	19.326845	9.196957	28.859871
H	18.122356	8.489025	30.937925
S	16.292770	9.762103	32.683806
C	15.043312	11.034468	33.076847
H	14.508250	10.640038	33.970303
H	15.519323	11.999894	33.355160
H	14.337433	11.161325	32.225489
C	16.309792	14.207247	28.740881
C	15.302483	13.621112	27.910140
C	16.047694	15.455140	29.385971
C	14.069078	14.294717	27.741884
C	14.797442	16.090923	29.188673
C	13.792020	15.531741	28.369448
H	13.296126	13.830675	27.096199
H	14.605324	17.056799	29.696823
C	19.405524	15.484117	23.761691
C	19.566506	16.904036	23.749095
C	18.349699	14.888624	23.001430
C	18.676755	17.695785	22.981664
C	17.486028	15.720483	22.250578
C	17.628837	17.127707	22.223547

H	18.812198	18.795678	22.976124
H	16.674641	15.246627	21.662549
C	15.512487	12.294840	27.206246
C	17.067951	16.135627	30.284326
C	12.475683	16.247821	28.143591
C	18.131814	13.388662	22.972437
C	20.663691	17.608723	24.531892
C	16.669039	17.994243	21.434647
H	16.375359	17.512447	20.474268
H	15.729625	18.176677	22.010585
H	17.111613	18.988616	21.199919
H	12.494509	16.825877	27.188237
H	11.623218	15.533714	28.077237
H	12.256317	16.970304	28.962005
H	20.597802	18.709138	24.381237
H	20.589765	17.424799	25.630291
H	21.686985	17.297004	24.212901
H	19.003351	12.853647	22.527247
H	17.989391	12.973864	23.998061
H	17.231571	13.132581	22.371581
H	18.014932	16.378107	29.745399
H	16.660783	17.094169	30.675454
H	17.342293	15.512423	31.168674
H	16.410976	12.315804	26.545643
H	15.666754	11.462310	27.932352
H	14.631950	12.039373	26.575872
H	18.267248	14.175975	29.625041
H	21.115687	15.295382	25.049702
Au	17.509043	9.973819	34.899835
Au	17.378241	8.255172	37.093906
Au	15.876212	10.827434	37.071006
Au	18.857874	10.810312	37.085946
Au	17.309080	6.612096	39.291649
Au	15.743525	9.055772	39.281922
Au	18.935307	9.056137	39.304721
Au	14.437602	11.644552	39.278762
Au	17.344755	11.811777	39.288582
Au	20.247725	11.675075	39.289819

11. References

(S1) You, W.; Wang, L; , Wang, Q.; Yu, L. Synthesis and Structure/Property Correlation of Fully Functionalized Photorefractive Polymers. *Macromolecules* **2002**, *35*, 4636–4645.

(S2) Bonifacio, M. C.; Robertson, C. R.; Jung, J.-Y.; King, B. T. Polycyclic Aromatic Hydrocarbons by Ring-Closing Metathesis *J. Org. Chem.* **2005**, *70*, 8522–8526.

(S3) Sheldrick, G. M. SHELXT–Integrated space-group and crystal-structure determination. *Acta Cryst.* **2015**, *A71*, 3–8.

(S4) (a) Sheldrick, G. M. A short history of SHELX. *Acta Cryst.* **2008**, *A64*, 112–122; (b) Sheldrick, G. M. Crystal structure refinement with SHELXL. *Acta Cryst.* **2015**, *C71*, 3–8.

(S5) Farrugia, L. J. WinGX and ORTEP for Windows: an update. *J. Appl. Cryst.* **2012**, *45*, 849–854.

(S6) Elgrishi, N.; Rountree, K. J.; McCarthy, B. D.; Rountree, E. S.; Eisenhart, T. T.; Dempsey, J. L. A Practical Beginner's Guide to Cyclic Voltammetry. *J. Chem. Educ.* **2018**, 95, 197–206.

(S7) (a) Cabosart, D.; El Abassi, M.; Stefani, D.; Frisenda, R.; Calame M.; van der Zant, H. S. J.; Perrin, M. L. A Reference-Free Clustering Method for the Analysis of Molecular Break-Junction Measurements. *Appl. Phys. Lett.* **2019**, *114*, 143102; (b) El Abbassi, M.; Zwick, P.; Rates, A.; Stefani, D.; Prescimone, A.; Mayor, M.; van der Zant, H. S. J.; Dulić, D. Unravelling the Conductance Path through Single-Porphyrin Junctions. *Chem. Sci.* **2019**, *10*, 8299–8305. (c) Lin, L.; Tang, C.; Chen, Z.; Pan, Z.; Liu, J.; Yang, Y.; Shi, J.; Ji, R.; Hong, W. Spectral Clustering to Analyze the Hidden Events in Single-Molecule Break Junctions. *J. Phys. Chem. C* **2021**, *125*, 3623–3630.

(S8) Quek, S. Y.; Kamenetska, M.; Steigerwald, M. L.; Choi, H. J.; Louie, S. G.; Hybertsen, M. S.; Neaton, J. B.; Venkataraman, L. Mechanically Controlled Binary Conductance Switching of a Single-Molecule Junction. *Nat. Nanotechnol.* **2009**, *4*, 230–234.

(S9) Palomino-Ruiz, L.; Reiné, P.; Márquez, I. R.; Álvarez de Cienfuegos, L.; Agraït, N.; Cuerva, J. M.; Campaña, A. G.; Leary, E.; Miguel, D.; Millán, A.; Zotti, L. A.; González, M. T. Three-State Molecular Potentiometer Based on a Non-Symmetrically Positioned in-Backbone Linker. *J. Mater. Chem. C* **2021**, *9*, 16282–16289.

(S10) (a) Li, C.; Pobelov, I.; Wandlowski, T.; Bagrets, A.; Arnold, A.; Evers, F. Charge Transport in Single Au | Alkanedithiol | Au Junctions: Coordination Geometries and Conformational Degrees of Freedom. *J. Am. Chem. Soc.* **2008**, *130*, 318–326; (b) González, M. T.; Brunner, J.; Huber, R.; Wu, S.; Schönenberger, C.; Calame, M. Conductance Values of Alkanedithiol Molecular Junctions. *New J. Phys.* **2008**, *10* ,065018.

(S11) González, M. T.; Leary, E.; García, R.; Verma, P.; Herranz, M. Á.; Rubio-Bollinger, G.; Martín, N.; Agraït, N. Break-Junction Experiments on Acetyl-Protected Conjugated Dithiols under Different Environmental Conditions. *J. Phys. Chem. C* **2011**, *115*, 17973–17978.

(S12) (a) Leary, E.; Zotti, L. A.; Miguel, D.; Márquez, I. R.; Palomino-Ruiz, L.; Cuerva, J. M.; Rubio-Bollinger, G.; González, M. T.; Agrait, N. The Role of Oligomeric Gold–Thiolate Units in Single-Molecule Junctions of Thiol-Anchored Molecules. *J. Phys. Chem. C* **2018**, *122*, 3211–3218; (b) Vladyka, A.; Perrin, M. L.; Overbeck, J.; Ferradás, R. R; García-Suárez, V.; Gantenbein, M.; Brunner, J.; Mayor, M.; Ferrer, J.; Calame, M. In-Situ Formation of One-Dimensional Coordination Polymers in Molecular Junctions. *Nat. Commun.* **2019**, *10*, 262.

(S13) Lafferentz, L.; Ample, F.; Yu, H.; Hecht, S.; Joachim, C.; Grill, L. Conductance of a Single Conjugated Polymer as a Continuous Function of Its Length. *Science* **2009***, 323,* 1193–1197.

(S14) (a) Mishra, S.; Fatayer, S.; Fernández, S.; Kaiser, K.; Peña, D.; Gross, L. Nonbenzenoid High-Spin Polycyclic Hydrocarbons Generated by Atom Manipulation. *ACS Nano* **2022**, *16*, 3264–3271; (b) Wang, T.; Berdonces-Layunta, A.; Friedrich, N.; Vilas-Varela, M.; Calupitan, J. P.; Pascual, J. I.; Peña, D.; Casanova, D.; Corso, M.; de Oteyza, D. G. Aza-Triangulene: On-Surface Synthesis and Electronic and Magnetic Properties. *J. Am. Chem. Soc.* **2022**, *144*, 4522–4529.

(S15) Gaussian 16, Revision C.01, Frisch, M. J.; Trucks, G. W.; Schlegel, H. B.; Scuseria, G. E.; Robb, M. A.; Cheeseman, J. R.; Scalmani, G.; Barone, V.; Petersson, G. A.; Nakatsuji, H.; Li, X.; Caricato, M.; Marenich, A. V.; Bloino, J.; Janesko, B. G.; Gomperts, R.; Mennucci, B.; Hratchian, H. P.; Ortiz, J. V.; Izmaylov, A. F.; Sonnenberg, J. L.; Williams-Young, D.; Ding, F.; Lipparini, F.; Egidi, F.; Goings, J.; Peng, B.; Petrone, A.; Henderson, T.; Ranasinghe, D.; Zakrzewski, V. G.; Gao, J.; Rega, N.; Zheng, G.; Liang, W.; Hada, M.; Ehara, M.; Toyota, K.; Fukuda, R.; Hasegawa, J.; Ishida, M.; Nakajima, T.; Honda, Y.; Kitao, O.; Nakai, H.; Vreven, T.; Throssell, K.; Montgomery, J. A., Jr.; Peralta, J. E.; Ogliaro, F.; Bearpark, M. J.; Heyd, J. J.; Brothers, E. N.; Kudin, K. N.; Staroverov, V. N.; Keith, T. A.; Kobayashi, R.; Normand, J.; Raghavachari, K.; Rendell, A. P.; Burant, J. C.; Iyengar, S. S.; Tomasi, J.; Cossi, M.; Millam, J. M.; Klene, M.; Adamo, C.; Cammi, R.; Ochterski, J. W.; Martin, R. L.; Morokuma, K.; Farkas, O.; Foresman, J. B.; Fox, D. J. Gaussian, Inc., Wallingford CT, 2016.

(S16) GaussView, Version 6.1, Dennington, R.; Keith, T. A.; Millam, J. M. Semichem Inc., Shawnee Mission, KS, 2016.

(S17) (a) Soler, J. M.; Artacho, E.; Gale, J. D.; Garcia, A.; Junquera, J.; Ordejon, P.; Sanchez-Portal, D. The SIESTA Method for Ab Initio Order-*N* Materials Simulation. *J. Phys.: Condens. Matter.* **2002**, *14*, 2745; (b) Brandbyge, M.; Mozos, J.-L.; Ordejón, P.; Taylor, J.; Stokbro, K. Density-Functional Method for Nonequilibrium Electron Transport. *Phys. Rev. B* **2002**, *65*, 165401; (c) Papior, N; Lorente, N.; Frederiksen, T.; García, A.; Brandbyge, M. Improvements on Non-

Equilibrium and Transport Green Function Techniques: The Next-Generation TRANSIESTA. *Comput. Phys. Commun.* **2017**, *212*, 8–24.

(S18) Kokalj, A. Computer Graphics and Graphical User Interfaces as Tools in Simulations of Matter at the Atomic Scale. *Comp. Mater. Sci.* **2003**, *28*, 155–168.

(S19) Nakano, M. Electronic Structure of Open-Shell Singlet Molecules: Diradical Character Viewpoint. *Top. Curr. Chem.* **2017**, *375*, 47.

(S20) Casares, R.; Martínez-Pinel, Á.; Rodríguez-González, S.; Márquez, I. R.; Lezama, L.; González, M. T.; Leary, E.; Blanco, V.; Fallaque, J. G.; Díaz, C.; Martín, F.; Cuerva, J. M.; Millán, A. Engineering the HOMO–LUMO Gap of Indeno[1,2-*b*]fluorene. *J. Mater. Chem. C* **2022**, *10*, 11775–11782.

(S21) (a) Perdew, J. P.; Burke, K.; Ernzerhof, M. Generalized Gradient Approximation Made Simple. *Phys. Rev. Lett.* **1996**, *77*, 3865; (b) Perdew, J. P.; Burke, K.; Ernzerhof, M. Generalized Gradient Approximation Made Simple. *Phys. Rev. Lett.* **1997**, *78*, 1396; (c) Zhang, Y.; Yang, W. Comment on "Generalized Gradient Approximation Made Simple". *Phys. Rev. Lett.* **1998**, *80*, 890.

(S22) (a) Lambert, C. J. Basic Concepts of Quantum Interference and Electron Transport in Single-Molecule Electronics*, Chem. Soc. Rev.* **2015**, *44*, 875—888; (b) O`Driscoll, L. J.; Bryce, M. R. Extended Curly Arrow Rules to Rationalize and Predict Structural Effects on Quantum Interference in Molecular Junctions. *Nanoscale* **2021**, 13, 1103–1123; (c) Zotti, L. A.; Leary, E. Taming Quantum Interference in Single Molecule Junctions: Induction and Resonance are Key. *Phys.Chem.Chem.Phys*. **2020**, *22*, 5638—5646; (d) O'Driscoll, L. J.; Sangtarash, S.; Xu, W.; Daaoub, A.; Hong, W.; Sadeghi, H.; Bryce, M. R. Heteroatom Effects on Quantum Interference in Molecular Junctions: Modulating Antiresonances by Molecular Design, *J. Phys. Chem. C* **2021**, *125*, 17385−17391.

(S23) (a) Zheng, Y.; Duan, P.; Zhou, Y.; Li, C.; Zhou, D.; Wang, Y.; Chen, L.-C.; Zhu, Z.; Li, X.; Bai, J.; Qu, K.; Gao, T.; Shi, J.; Liu, J.; Zhang, Q.-C.; Chen, Z.-N.; Hong, W. Fano Resonance in Single-Molecule Junctions. *Angew. Chem., Int. Ed.* **2022**, *61*, e202210097; (b) Zou, Q.; Chen, X.; Zhou, Y.; Jin, X.; Zhang, Z.; Qiu, J.; Wang, R.; Hong, W.; Su, J.; Qu, D.-H.; Tian, H. Photoconductance from the Bent-to-Planar Photocycle between Ground and Excited States in Single-Molecule Junctions *J. Am. Chem. Soc.* **2022**, *144*, 10042–10052; (c) Prindle, C. R.; Shi, W.; Li, L.; Jensen, J. D.; Laursen, B. W.; Steigerwald, M. L.; Nuckolls, C.; Venkatamaran, L. Effective Gating in Single-Molecule Junctions through Fano Resonances. *J. Am. Chem. Soc*. **2024**, 146, 3646-3650.

(S24) Yin, X.; Zang, Y.; Xhu, L.; Low, J. Z.; Liu, X.-F.; Cui, J.; Neaton, J. B.; Venkataraman, L.; Campos, L. M. A Reversible Single-Molecule Switch Based on Activated Antiaromaticity. *Sci Adv.* **2017**, *3*, eaao2615.
